# N6-methyladenosine-modified *circPLPP4* sustains cisplatin resistance in ovarian cancer cells via PIK3R1 upregulation

**DOI:** 10.1186/s12943-023-01917-5

**Published:** 2024-01-06

**Authors:** Han Li, Run Lin, Yanna Zhang, Yanni Zhu, Shuting Huang, Jing Lan, Nian Lu, Chuanmiao Xie, Shanyang He, Weijing Zhang

**Affiliations:** 1 Department of Gynecology, Guangdong Provincial People’s Hospital (Guangdong Academy of Medical Sciences), Southern Medical University, Guangzhou, Guangdong China; 2 Department of Radiology, The First Affiliated Hospital, Sun Yat-Sen University, Guangzhou, 510080 Guangdong China; 3 Department of Gynecology, State Key Laboratory of Oncology in South China, Guangdong Provincial Clinical Research Center for Cancer, Sun Yat-sen University Cancer Center, No. 651 Dongfeng Road East, Guangzhou, Guangdong 510060 China; 4 Department of Radiology, State Key Laboratory of Oncology in South China, Guangdong Provincial Clinical Research Center for Cancer, Sun Yat-sen University Cancer Center, No. 651 Dongfeng Road East, Guangzhou, Guangdong 510060 China

**Keywords:** CDDP resistance, Ovarian cancer, Circular RNAs, PIK3R1, m^6^A

## Abstract

**Background:**

Cisplatin (CDDP) is the first-line chemotherapeutic strategy to treat patients with ovarian cancer (OC). The development of CDDP resistance remains an unsurmountable obstacle in OC treatment and frequently induces tumor recurrence. Circular RNAs (circRNAs) are noncoding RNAs with important functions in cancer progression. Whether circRNAs function in CDDP resistance of OC is unclear.

**Methods:**

Platinum-resistant circRNAs were screened via circRNA deep sequencing and examined using in situ hybridization (ISH) in OC. The role of circPLPP4 in CDDP resistance was assessed by clone formation and Annexin V assays in vitro, and by OC patient-derived xenografts and intraperitoneal tumor models in vivo*.* The mechanism underlying circPLPP4-mediated activation of miR-136/PIK3R1 signaling was examined by luciferase reporter assay, RNA pull-down, RIP, MeRIP and ISH.

**Results:**

circPLPP4 was remarkably upregulated in platinum resistant OC. circPLPP4 overexpression significantly enhanced, whereas circPLPP4 silencing reduced, OC cell chemoresistance. Mechanistically, circPLPP4 acts as a microRNA sponge to sequester miR-136, thus competitively upregulating PIK3R1 expression and conferring CDDP resistance. The increased circPLPP4 level in CDDP-resistant cells was caused by increased RNA stability, mediated by increased N6-methyladenosine (m^6^A) modification of circPLPP4*. *In vivo delivery of an antisense oligonucleotide targeting circPLPP4 significantly enhanced CDDP efficacy in a tumor model.

**Conclusions:**

Our study reveals a plausible mechanism by which the m^6^A -induced circPLPP4/ miR-136/ PIK3R1 axis mediated CDDP resistance in OC, suggesting that circPLPP4 may serve as a promising therapeutic target against CDDP resistant OC. A circPLPP4-targeted drug in combination with CDDP might represent a rational regimen in OC.

**Supplementary Information:**

The online version contains supplementary material available at 10.1186/s12943-023-01917-5.

## Background

Ovarian cancer (OC) is the main cause of mortality among gynecological cancers, and its mortality rate is predicted to rise remarkably [1]. Despite advances in screening and targeted therapy, the overall survival rate of OC has remained poor at nearly 35%. The poor prognosis of patients with OC is predominately caused by delayed symptom onset and Cisplatin (CDDP)-based chemotherapy resistance [2, 3]. Nearly 25% of patients with OC experience recurrence within six months after CDDP-based chemotherapy, and over half suffer relapse and become resistant to chemotherapy in three years, resulting in a significantly lower five-year survival than in other gynecological cancers [4, 5]. Therefore, to identify novel biomarkers to predict CDDP resistance and targeted therapy for advanced OC is clinically imperative. Various mechanisms contribute to CDDP resistance, including escape from CDDP-mediated apoptosis, interaction with the tumor environment, epigenetic modification, drug accumulation disorders, and enhanced DNA adduct repair. These factors usually coexist in the same tumor mass [6–8]. Recently, a study indicated that phosphoinositide-3-kinase regulatory subunit 1(PIK3R1)-activated PI3K/protein kinase B (AKT) signaling reduced sensitivity to CDDP in gastric cancer cells by inhibiting cell apoptosis and promoting cell survival [9]. PI3K is a heterodimer containing two subunits, an SH2-containing regulatory subunit (p85) and a p110 subunit functioning in catalysis [10]. The regulatory subunit is encoded by *PIK3R1*, *PIK3R2*, and *PIK3R3*, with *PIK3R1* encoding the major regulatory subunit, p85α. Additionally, *PIK3R1* is upregulated in several cancers and is involved in tumor metastasis, chemotherapy resistance, and progression [10]. However, the role of PIK3R1 in cisplatin resistance in OC remains unreported.

circRNAs are a category of noncoding RNAs (ncRNAs) generated from exons of protein-coding genes and are vital regulators of numerous biological processes [11, 12]. Their unique circular structure makes circRNAs more stable than linear RNA and are resistant to degradation by RNA exonucleases, thus circRNAs have clinical advantages as biomarkers [11]. Evidence suggested that aberrant circRNA expression promotes the proliferation, metastasis, and progression of several cancers, including OC [13, 14]. CircRNAs exert their biological functions by acting as microRNA sponges, RNA binding protein (RBP) sponges, protein/peptide translation templates, and via RNA splicing regulation [15]. Among them, miRNA sponging is the most prevalent method employed by circRNAs in tumor development [16]. Despite several circRNAs being reported in OC, few CDDP resistance-related circRNAs and their functions and underlying mechanisms have been explored clearly.

N6-methyladenosine (m^6^A) is the most abundant modification of mRNAs and ncRNAs, which controls almost all steps regulating RNA fate, including stability, splicing export, and translation [17]. For example, *circFBXW7* encodes a novel 21 kDa protein, FBXW7-185aa, which inhibits the proliferation and cell cycle acceleration of glioma cells. The translation process was driven by m^6^A modification [18]. Moreover, the m^6^A modification facilitates circRNA degradation. The m^6^A modification of *circNDUFB2* enhanced the formation of TRIM25/*circNDUFB2*/IGF2BPs ternary complexes, ultimately promoting IGF2BP ubiquitination and degradation, which inhibits lung cancer cell proliferation and metastasis [19]. Additionally, m^6^A modification of *circNSUN2* contributes to colorectal cancer liver metastasis by inducing its cytoplasmic export and the formation a circNSUN2/IF2BP2/HMGA2 complex that stabilizes HMGA2 [20]. However, the regulatory role of m^6^A in the biogenesis and function of circRNAs remains to be addressed. Therefore, the role of m^6^A-modified circRNAs in OC CDDP resistance requires further exploration.

The present study aimed to identify CDDP resistance-related circRNAs in OC tissues (chemo-resistant vs. chemo-sensitive tissues), and to investigate their mechanism and association with survival.

## Methods

### Cell lines

The human OC cell line SKOV3 was obtained from the American Type Culture Collection (Manassas, VA, USA), A2780 cells were obtained from the National Institutes for Food and Drug Control (Dongcheng District, Beijing, China). A2780-CDDP and SKOV3-CDDP were a kind gift from Prof. Jun Li. All these cells were authenticated using short tandem repeat profiling. The cell lines were grown in Dulbecco’s modified Eagle’s medium (DMEM) (Gibco, Grand Island, NY, USA) with 10% fetal bovine serum (Gibco) following the manufacturer’s instructors.

### Clinical samples

Clinical samples were obtained from SYSUCC (Guangzhou, China), comprising 166 paraffin-embedded OC samples that were histopathologically and clinically diagnosed at SYSUCC from 2001 to 2013. After surgery, the patients received regular follow-up. In addition, 40 OC tissues (including 20 OC patients with CDDP resistance and 20 OC patients with CDDP sensitivity) were obtained from SYSUCC and Guangdong Provincial People’s Hospital. According to National Comprehensive Cancer Network (NCCN) guidelines, in our study, platinum resistant ovarian cancer was defined as: complete remission and relapse < 6 months after completing surgery and chemotherapy. According to NCCN guidelines , in our study, platinum sensitive ovarian cancer was defined as: complete remission and relapse **≥** 6 months after completing prior chemotherapy. Clinical information of the OC patients is summarized in Table S1. This study was approved by the institutional research ethics committee of Sun Yat-sen University Cancer Center and Guangdong Provincial People’s Hospital (B2021-396 and KY-Q-2021-097-02).

### RNA sequencing of circRNA extracted from human OC tissues

Total RNA was extracted from five OC tissues with CDDP resistance and five OC tissues without CDDP resistance using the TRIzol reagent (Takara, Dalian, China) and further purified by rRNA depletion, followed by cDNA synthesis, and RNA amplification, following the manufacturer’s instructions. The RNA-seq libraries were constructed and sequenced using the Illumina HiSeq2500 platform (Illumina, San Diego, CA, USA).

## Results

### *CircPLPP4* is upregulated in CDDP-resistant OC cells and tissues

**Fig. 1:**
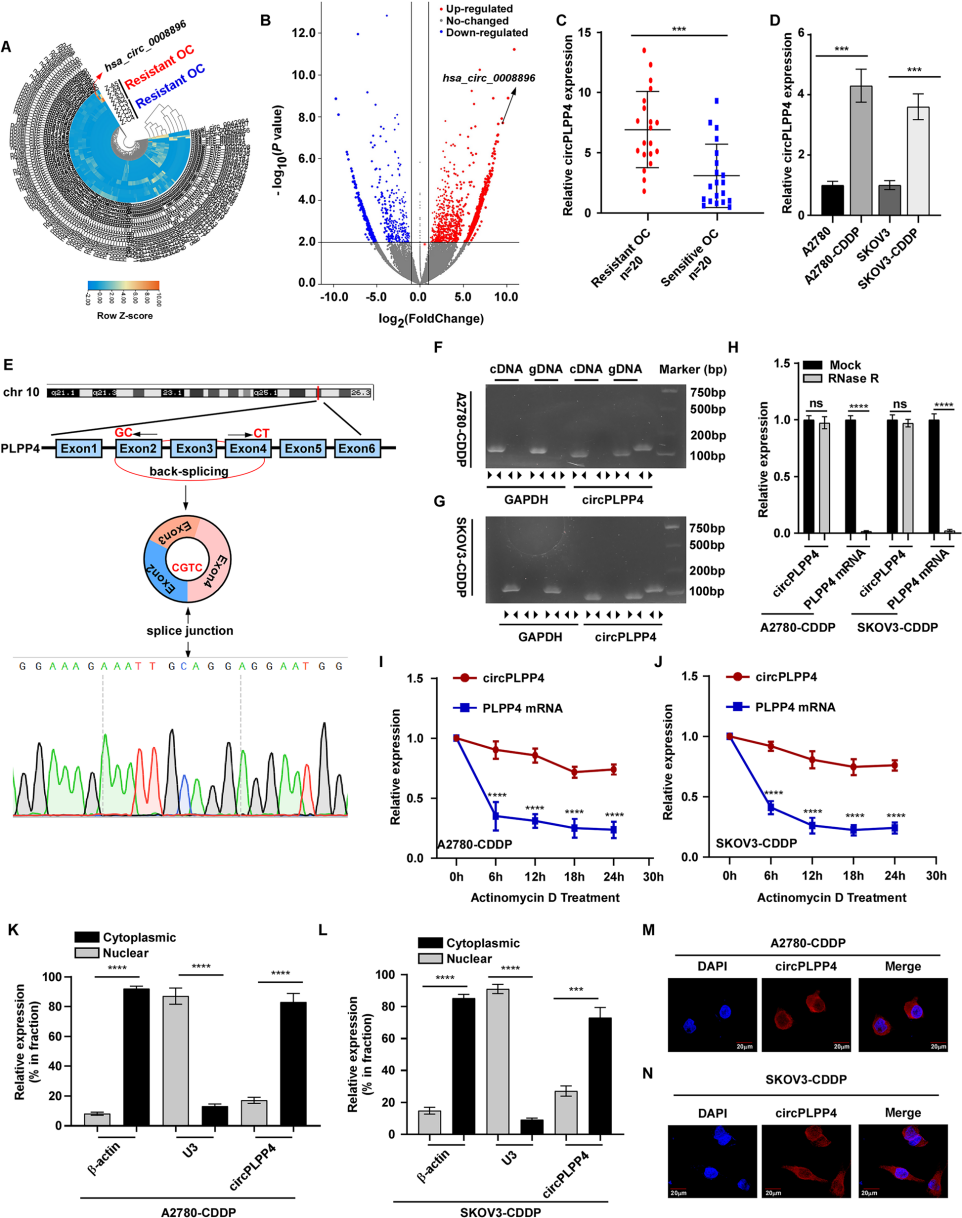
circPLPP4 expression is increased in CDDP-resistant OC cells and tissues. **A**, **B** Unsupervised hierarchical clustering and volcano plot analysis of the circRNAs differentially expressed in Platinum resistant OC tissues and Platinum sensitive OC tissues. **C** qRT-PCR analysis of *circPLPP4* expression in a 20-case cohort of freshly collected human OC samples with Platinum resistance and 20-case cohort of Platinum sensitive OC samples. The nonparametric Mann–Whitney U-test was used. **D** qRT-PCR analysis of *circPLPP4* in two CDDP resistant OC cells and its corresponding parent cell. **E** Schematic illustration demonstrated the circularization of exons 2–4 of *PLPP4* forms *circPLPP4* by “head-to-tail” junction and the upper black arrow represents the splicing sites. **F**, **G** *circPLPP4* expression in A2780-CDDP and SKOV3-CDDP cells examined by RT-PCR. Agarose gel electrophoresis demonstrated that divergent primers amplified *circPLPP4* in cDNA rather than genomic DNA (gDNA). GAPDH acted as a negative control. **H** RT–qPCR analysis for the expression of *circPLPP4* and *PLPP4* mRNA after treatment with RNase R in A2780-CDDP and SKOV3-CDDP cells. Data represent mean ± S.D. from three independent experiments; The P value was determined by a two-tailed unpaired Student’s t test. **I**, **J** qRT–PCR analysis for the expression of *circPLPP4* and *PLPP4* mRNAs after Actinomycin D treatment at the indicated time in A2780-CDDP and SKOV3-CDDP cells. Data represent mean ± S.D. from three independent experiments; The P value was evaluated by a two-way ANOVA. **K**, **L** Cytoplasmic and Nuclear mRNA Fractionation experiment indicating that *circPLPP4* mainly localized in the cytoplasm. β-actin and U3 were applied as positive controls in the cytoplasm and nucleus, respectively. Data represent mean ± S.D. from three independent experiments; The P value was evaluated by a two-tailed unpaired Student’s t test. **M**, **N**. FISH for *circPLPP4*. Nuclei were stained with DAPI. * *P* < 0.05, ** *P* < 0.01, *** *P* < 0.001, **** *P* < 0.0001, ns indicates no significance. Each error bar represents the mean ± SD of three independent experiments

To identify the crucial circRNAs that induce CDDP resistance of OC, RNA-Seq analysis was conducted in five CDDP-resistant and five CDDP-sensitive OC tissues. Unsupervised hierarchical clustering and volcano plot showed that 3218 upregulated and 1891 downregulated circRNAs in CDDP-resistant OC tissues compared with CDDP-sensitive tissues according to the Log ^(fold−change)^ ≥ 2 and* P*-value < 0.05 (Fig. 1A, B). Consistent with the RNA-Seq results, qRT-PCR identified *hsa_circ_0008896* as markedly upregulated in CDDP resistant OC cells and OC tissues identified *hsa_circ_0008896* as markedly upregulated in CDDP resistant OC cells and OC tissues (Fig. 1C, D). Moreover, there was no significant difference in the expression level of *PLPP4* mRNA in the patients with CDDP resistant ovarian cancer compared with the patients with CDDP-sensitive ovarian cancer (Supplemental Fig. 1A). qRT-PCR analysis also showed that circPLPP4 significantly upregulated in two CDDP resistant OC cells and its corresponding parent cells (Fig. 1D). Additionally, we further detected circPLPP4 expression in paired biopsies obtained before and after platinum-based chemotherapy. As shown in Supplemental Fig. 2A-B, the expression of circPLPP4 was significantly higher in these after cisplatin-based chemotherapy OC biopsies tissues compared to that before cisplatin-based chemotherapy in cisplatin-resistant ovarian cancer. However, the expression of circPLPP4 was no statistically significant difference in the paired biopsies obtained before and after platinum-based chemotherapy of cisplatin-sensitive ovarian cancer. According to circBase annotation [21], *hsa_circ_0008896* is generated from exon 2 to 4 of the PLPP4 transcript (named as circPLPP4) by back-splicing, which was validated by Sanger sequencing (Fig. 1E).

**Fig. 2:**
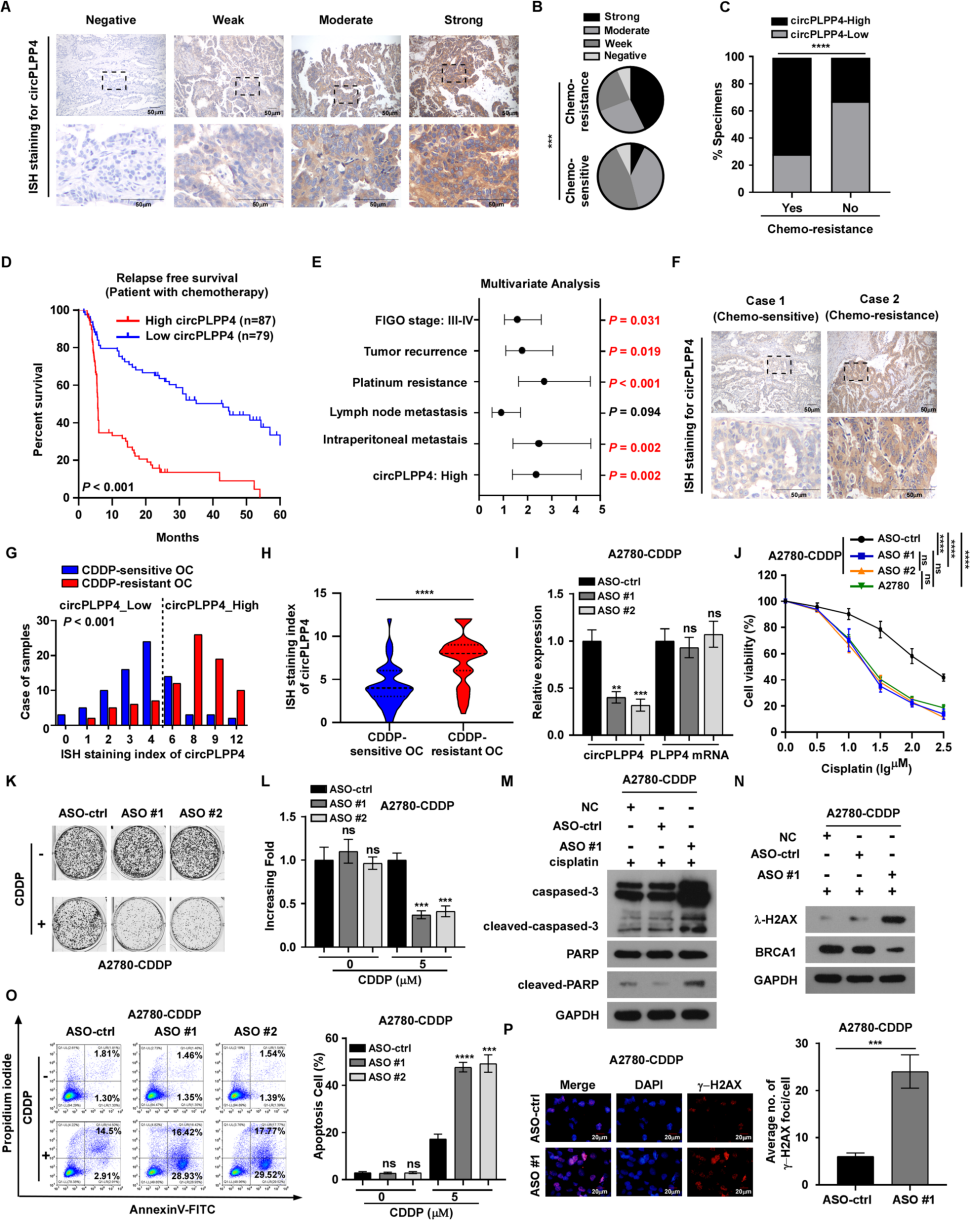
CircPLPP4 expression level is associated with poor prognosis in OC patients and silencing of circPLPP4 facilitate CDDP sensitivity of OC cells. **A** Representative sections of *circPLPP4* in 166 OC tissues using in situ hybridization. **B** The distribution of *circPLPP4* in Platinum resistant and Platinum sensitive patient specimens detected by in situ hybridization (ISH). χ2 test was used. **C** *circPLPP4* is remarkably relevant with the response status of chemotherapy. χ2 test was used. **D** Kaplan–Meier analysis of 5-year Relapse-free survival (RFS) in OC patients stratifed by low and high *circPLPP4* levels (*n* = 166, log-rank test). HR, hazard ratio. **E** Multivariate Cox regression analysis to assess the significance of the correlation between *circPLPP4* expression signature and OS in the presence of other important clinical variables. **G**, **H** ISH analysis of *circPLPP4* expression in OC tissues (92 Platinum sensitive OC patients and 72 Platinum resistant OC patients). **I** Staining index of *circPLPP4* in 166 OC tissues (92 Platinum sensitive OC patients and 72 Platinum resistant OC patients). **J** qRT-PCR analysis of *circPLPP4* and linear PLPP4 expression in the indicated OC cells. GAPDH act as a control. **K** MTT cell viability assay of CDDP in the indicated cells. **K**, **L** Representative images and quantification of colony number of the indicated cells. (M) Western blot analysis shows apoptotic proteins in the indicated cells (GAPDH was selected as the loading control). **N** Western blotting analysis of level of γ-H2AX and BRCA1 in the indicated cells. (GAPDH was acted as the loading control). **O** FACS analysis of Annexin V / PI staining (left) and quantification (right) of indicated cells treated with vehicle or CDDP (5 μM) after 24 h. **P** Representative images (left) and quantification (right) of γ-H2AX in the indicated OC cells treated with CDDP (5 μM) after 24 h. * *P* < 0.05, ** *P* < 0.01, *** *P* < 0.001, **** *P* < 0.0001, ns indicates no significance. Each error bar represents the mean ± SD of three independent experiments

qRT-PCR analyses using divergent and convergent primers confirmed that circPLPP4 was formed by back-splicing rather than genomic rearrangements or trans-splicing. RT–PCR revealed that circPLPP4 could only be detected in cDNA, whereas *PLPP4* was amplified from both cDNA and gDNA, which also excluded the possibility of circPLPP4 formation from genomic rearrangements or trans-splicing (Fig. 1F, G). Moreover, the linear form of PLPP4, but not circPLPP4, was easily digested by RNase R (Fig. 1H). Actinomycin D was used to inhibit transcription and examine the half-life of *circPLPP4* in A2780 CDDP and SKOV3 CDDP cells, which showed that that *circPLPP4* was more stable than *PLPP4* mRNA (Fig. 1I, J). Subsequently, qRT-PCR assays showed that *circPLPP4* is located mainly in the cytoplasm of OC cells (Fig. 1K, L). FISH analysis also demonstrated that *circPLPP4* is predominately distributed in the cytoplasm of A2780 CDDP and SKOV3 CDDP cells (Fig. 1M, N).

### *CircPLPP4* expression correlates with poor prognosis in patients with OC

The clinical significance of circPLPP4 expression was further evaluated in 166 OC specimens (Table S1). ISH staining revealing that circPLPP4 expression was upregulated significantly in OC specimens (Fig. 2A, B). Moreover, correlation analysis showed that high circPLPP4 expression was markedly associated with CDDP resistance and patient vital status (Fig. 2C, Supplemental Fig. 1B). Importantly, patients with OC with high circPLPP4 expression experienced significantly shorter overall/relapse-free survival among patients receiving platinum-based treatment (Fig. 2E. Supplemental Fig. 1C). High circPLPP4 expression could serve as an independent prognostic factor for the prognosis of tumor CDDP resistance and patient survival (Fig. 2F). Notably, ISH staining showed that circPLPP4 expression was increased significantly in patients with CDDP resistant OC (Fig. 2G-H).

### Downregulation of *circPLPP4* enhances CDDP sensitivity of CDDP-resistant OC cells in vitro

Next, w**e** assessed the effect of silencing circPLPP4 using a antisense oligonucleotides (ASOs) targeting the junction site of circPLPP4 on CDDP resistant cells (A2780 CDDP and SKOV3 CDDP cells) with high circPLPP4 expression. qRT-PCR showed that circPLPP4-ASO treatment markedly reduced circPLPP4 expression, but not *PLPP4* mRNA expression (Fig. 2I, Supplemental Fig. 3A). CircPLPP4 inhibition decreased the viability of A2780 CDDP and SKOV3 CDDP cells under cisplatin treatment (Fig. 2J, Supplemental Fig. 3B). Additionally, circPLPP4 silencing reduced the number of cell colonies and increased the proportion of apoptotic cells after cisplatin treatment significantly (Fig. 2K, L, O; Supplemental Fig. 3C-E). Western blotting was used to explore the underlying mechanisms of these functions. Under cisplatin treatment, circPLPP4 silencing in A2780 CDDP and SKOV3 CDDP cells enhanced the protein level of cleaved caspase-3 and cleaved PARP (activated form) (Fig. 2M, Supplemental Fig. 3F). Cisplatin induces DNA crosslinking and promotes H2AX phosphorylation. γ-H2AX is used as a sensitive marker of DNA damage and is known to bind to the BRCT (BRCA1 car-boxyl-terminal) domain of the *BRCA1* gene, which predicts sensitivity to cisplatin treatment [22, 23]. Importantly, circPLPP4-ASO treatment markedly increased the protein level of γ-H2AX compared with that in ASO-control cells (Fig. 2N, P, Supplemental Fig. 3G, H). Knockdown of circPLPP4 in A2780 CDDP and SKOV3 CDDP cells decreased BRCA1 expression (Fig. 2N, Supplemental Fig. 3G). Moreover, circPLPP4 overexpression in OC cells had the opposite effects (Supplemental Fig. 4A-K).

**Fig. 3:**
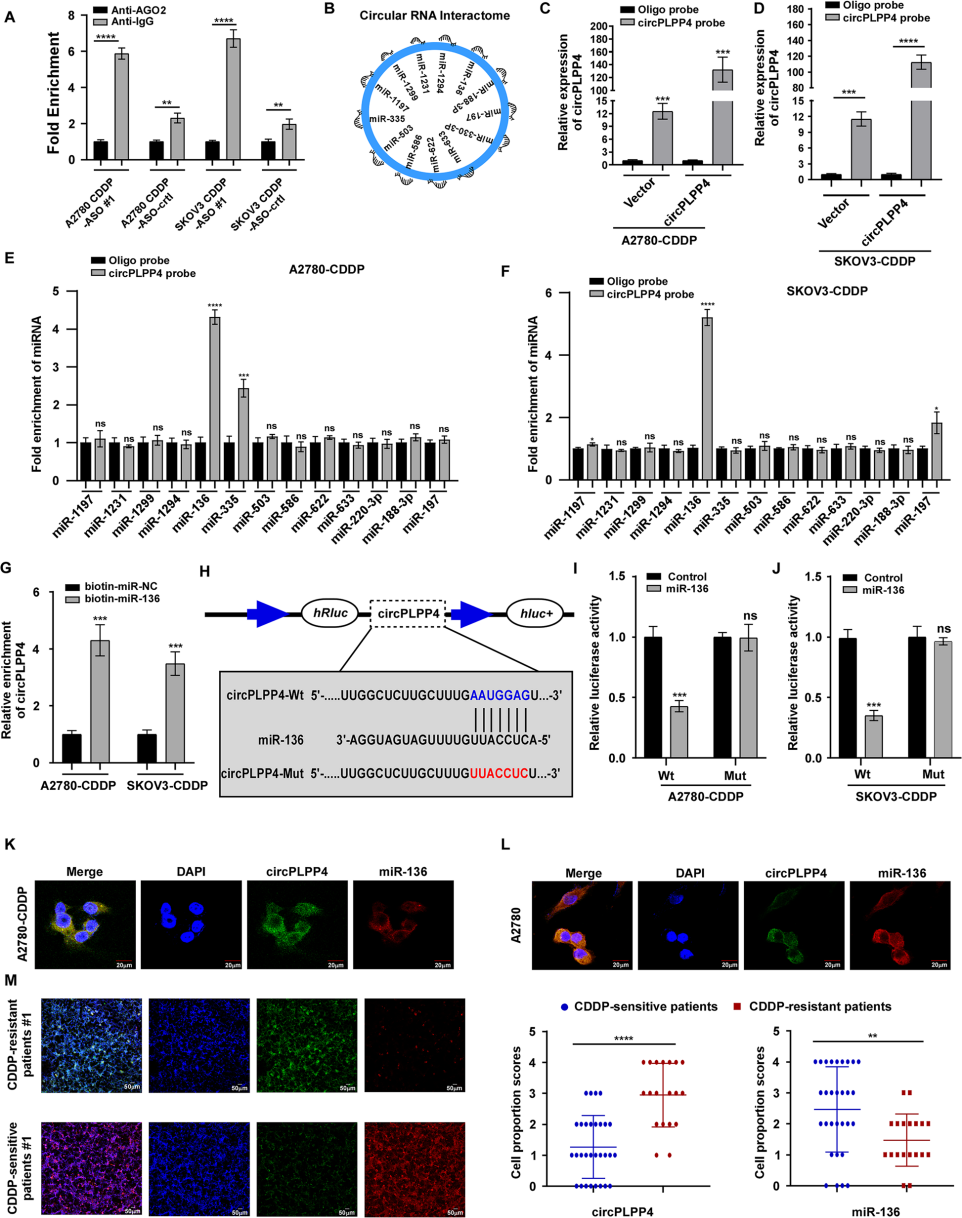
circPLPP4 exerts its function by sponging miR-136. **A** Ago2-RNA RIP assay for *circPLPP4* expression in the indicated cells. **B** Schematic illustration indicating potential target miRNAs of *circPLPP4* as predicted by CircInteractome. **C**, **D** Gel electrophoresis and qRT-PCR were used to validated the specificity and efficiency of the *circPLPP4* probe in A2780-CDDP and SKOV3-CDDP cells. **E**, **F** qRT-PCR analysis of the expression of thirteen potential target miRNAs in A2780-CDDP and SKOV3-CDDP cells. MiR-136 was stably pulled down by *circPLPP4* in both A2780-CDDP and SKOV3-CDDP cells. **G** Biotinylated miRNA pull-down (WT or mut) and qRT-PCR assays indicating the levels of *circPLPP4* in the indicated OC cells. GAPDH was acted as the negative control. **H** Schematic illustration of *circPLPP4*-wt and *circPLPP4*-mut luciferase reporter vectors; the binding ability between *circPLPP4* and MiR-136 was measured by dual-luciferase reporter assay in A2780-CDDP and SKOV3-CDDP cells. **I**, **J** The luciferase activities of the *circPLPP4* luciferase reporter vector (WT or mut) assessed after transfection with miR-136 mimics or mimic NC into A2780-CDDP and SKOV3-CDDP cells. **K**, **L**. FISH showing the expression of *circPLPP4* and miR-136 in A2780-CDDP and A2780 cells. Nuclei were stained with DAPI. (M) Platinum-resistant or Platinum-sensitive OC tissues from patients. FISH scores of *circPLPP4* and miR-136 were further evaluated in 30 Platinum-resistant and 19 Platinum-sensitive patient tissues. Nuclei were stained with DAPI. * *P* < 0.05, ** *P* < 0.01, *** *P* < 0.001, **** *P* < 0.0001, ns indicates no significance. Each error bar represents the mean ± SD of three independent experiments

**Fig. 4:**
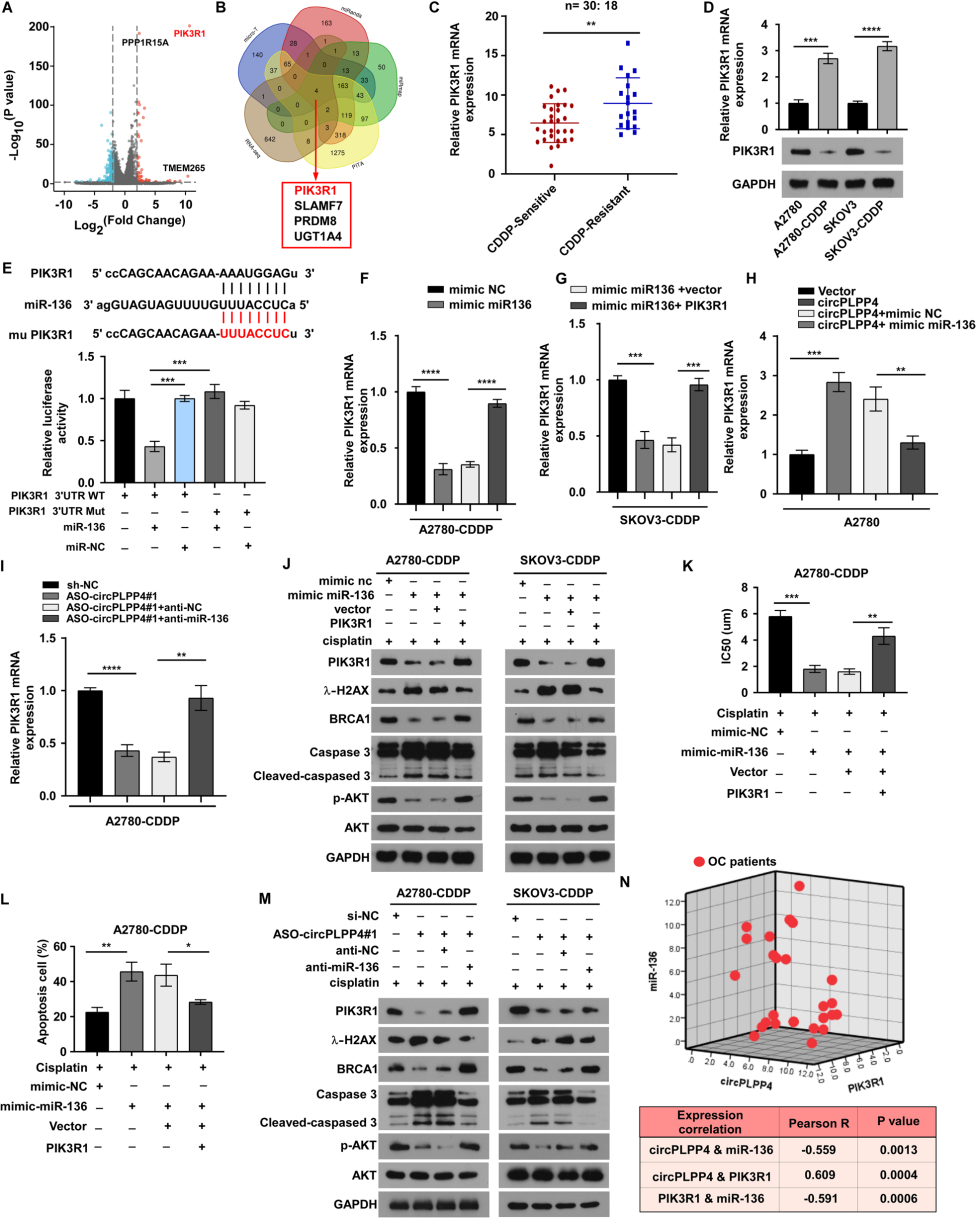
circPLPP4 enhanced PIK3R1 expression by sponging miR-136 in EOC cells. **A** RNA-seq data of the top 20 up-regulated mRNAs in A2780 CDDP cells and A2780 CDDP-ASO#1 cells are showed as heatmaps. **B** Venn diagram demonstrated 4 genes that are putative miR-136 targets computationally predicted by four algorithms (miRanda, RNAhybrid, miRWalk and TargetScan) within the top 20 upregulated genes. **C** The expression of PIK3R1 was analyzed using RT-qPCR and western blot in A2780 CDDP, SKOV3 CDDP, A2780 and SKOV3 cells. **D** The expression of PIK3R1 was assessed using RT-qPCR and western blot in A2780 CDDP, SKOV3 CDDP, A2780 and SKOV3 cells. **E** Schematic of PIK3R1 3’UTR wild-type (WT) and mutant (Mut) luciferase reporter vectors is displayed (Top). The relative luciferase activities were examined in A2780CDDP cells co-transfected with miR-136 mimics or miR-NC and luciferase reporter vectors PIK3R1 3’UTR (WT) or PIK3R1 3’UTR (Mut) (Bottom). **F**, **G** The expression of PIK3R1 was detected by RT-qPCR in A2780 CDDP and SKOV3 CDDP cells were transfected with miR-126 mimic or co-transfected with the indicated vectors. **H** The expression levels of PIK3R1 were detected using RT-qPCR. A2780 cells were transfected with the indicated vectors and miR-136 mimics. **I** The expression levels of PIK3R1 were analyzed using RT-qPCR. A2780 CDDP cells were transfected with ASO-circPLPP4#1 alone or co-transfected the inhibitors. (J)The expression of PIK3R1, γ-H2AX, BRCA1, p-AKT, AKT and cleaved caspased 3 was examined by western blot. A2780 CDDP and SKOV3 CDDP cells were transfected with miR-136 mimic or co-transfected with the indicated vectors. **K** The IC50 was examined by the MTT assay. A2780CDDP cells were transfected with miR-136 mimic alone or cotransfected with the indicated vectors with CDDP treatment (5 μM) for 48 h. **L** The apoptosis percentages of A2780 CDDP cells transfected with miR-136 mimic alone or cotransfected with the indicated vectors upon CDDP exposure (5 μM) for 48 h. **M** The levels of PIK3R1 and apoptosis markers, γ-H2AX, BRCA1 and cleaved-caspased 3 were detected using western blotting in A2780 CDDP cells and SKOV3 CDDP cells transfected with ASO-circPLPP4#1alone or co-transfected with the inhibitor after CDDP treatment (5 μM). **N** Three-dimensional scatter plot of circPLPP4, miR-136 and PIK3R1 levels in 25 OC tissues. The results are displayed as the mean ± SEM. * *P* < 0.05, ** *P* < 0.01, *** *P* < 0.001, **** *P* < 0.0001, ns indicates no significance. Each error bar represents the mean ± SD of three independent experiments

### circPLPP4 exerts its function by sponging miR-136

One of the methods by which circRNAs exert their function is by sponging miRNAs. Given that circPLPP4 locates mainly in the cytoplasm, we hypothesized that circPLPP4 promotes cisplatin resistance by binding miRNAs. First, we used an RIP assay using antibodies against argonaute 2 (AGO2) in A2780 CDDP and SKOV3 CDDP cells (Fig. 3A). Then, Circular RNA Interactome algorithm [24] was used to predict circRNA-miRNA interactions. This revealed that hsa-miR-1197, hsa-miR-1231, hsa-miR-1294, hsa-miR-1299, hsa-miR-136, hsa-miR-188-3p, hsa-miR-197, hsa-miR-330-3p, hsa-miR-335, hsa-miR-503, hsa-miR-586, hsa-miR-622, hsa-miR-633 might be associated with circPLPP4 (Fig. 3B). Next, we explored that whether these candidate miRNAs could bind directly to circPLPP4. A biotin-labeled circPLPP4 probe was verified to pull down circPLPP4 in A2780 CDDP and SKOV3 CDDP cells, and the pulldown efficiency increased in cell lines stably overexpressing circPLPP4 (Fig. 3C, D). Furthermore, qRT-PCR was performed to examine whether the 13 candidate miRNAs were pulled down by the circPLPP4 probe. Only miR-136 was abundantly pulled down in both A2780 CDDP and SKOV3 CDDP cells (Fig. 3E, F). Additionally, biotin-labeled miR-136 mimics were used to verify the direct binding of miRNA and *circPLPP4*: The biotin-labeled miR-136 captured more *circPLPP4* than the biotin-labeled negative control (Fig. 3G). A luciferase assay in A2780 CDDP and SKOV3 CDDP cells showed that the overexpression of miR-136 decreased the activity of the wild-type Luc-*circPLPP4* reporter gene. By contrast, overexpressed miR-136 had no effect on the activity of a Luc- circPLPP4-mutant reporter gene (Fig. 3H-J).

FISH showed colocalization of *circPLPP4* and miR-136 in A2780 CDDP (CDDP-resistant) and A2780 cells (CDDP-sensitive). The expression of circPLPP4 was markedly higher in A2780 CDDP cells than CDDP sensitive A2780 cells; whereas miR-136 expression showed the opposite results (Fig. 3K, L). Consistently, we found that *circPLPP4* was significantly higher, whereas miR-136 levels were obviously lower, in CDDP-resistant OC tissues than in CDDP-sensitive OC tissues (Fig. 3M).

### *circPLPP4* enhances PIK3R1 expression by sponging miR-136 in OC cells

In cancer cells, miRNAs mainly exert their function through regulating target genes. RNA-seq was performed on A2780 CDDP cells and A2780 CDDP circPLPP4 -ASO#1 cells to identify the targets gene(s) of miR-136 (Fig. 4A). Next, several algorithms (miRanda, RNAhybrid, miRWalk, and TargetScan) were used to predict the target genes of miR-136 (Fig. 4B). We considered these oncogenes or tumor suppressor genes as miR-136 targets if they met the following three criteria: (1) upregulated in A2780 CDDP cells compared with A2780 cells (Fig. 4D, Supplemental Fig. 5A-C); (2) elevated in CDDP-resistant OC tissues compared with CDDP sensitive tissues (Fig. 4C); (3) predicted as the potential miR-136 target genes by the four algorithms (Fig. 4B). Furthermore, we conducted luciferase reporter assays to determine whether miR-136 directly targets these selected genes in A2780 and SKOV3 cells (Fig. 4E, Supplemental Fig. 6A). In A2780 CDDP and SKOV3 CDDP cells co transfected with miR-136 mimics, reporter constructs including wild-type miR-136 binding sites from the *PIK3R1* 3′untranslated region (UTR) showed obviously reduced luciferase activity compared with that of constructs with mutated binding sites (Fig. 4E). Thus, we identified *PIK3R1* as the target gene of miR-136. Next, we validated the results by further functional examinations. We found that miR-136 mimics remarkably reduced the RNA levels of PIK3R1 and that ectopic *PIK3R1* expression repressed the effect caused by miR-136 overexpression (Fig. 4F-H, Supplemental Fig. 6B). Additionally, co-transfection of circPLPP4-ASO#1 and anti-miR-136 inhibited the circPLPP4-ASO#1-induced decreased in *PIK3R1* expression in A2780 CDDP and SKOV3 CDDP cells (Fig. 4I, Supplemental Fig. 6C). Notably, co-transfection of *circPLPP4* and miR-136 decreased the expression of *PIK3R1* compared with transfection of *circPLPP4* alone in A2780 and SKOV3 cells (Supplemental Fig. 6D, E). Furthermore, we found that miR-136 mimics markedly decreased the levels of PIK3R, BRCA1, and canonical PI3K/AKT signaling molecules and increased the CDDP response-related molecules, such as cleaved caspase3 and γ-H2AX (Fig. 4J). Ectopic *PIK3R1* expression reduced the effects of miR-136 upregulation (Fig. 4J). In addition, elevation of miR-136 expression repressed cell viability and induced apoptosis in A2780 CDDP and SKOV3 CDDP cells (Fig. 4K, L, Supplemental Fig. 6F, G). However, co-transfection of *PIK3R1* and miR-136 abrogated these effects (Fig. 4K, L, Supplemental Fig. 6F, G). Moreover, transfection of circPLPP4-ASO#1 significantly decreased PIK3R1, BRCA1 and p-AKT levels and upregulated the levels of cleaved caspase3 and γ-H2AX. Downregulating of both *circPLPP4* and miR-136 abrogated these effects in A2780 CDDP and SKOV3 CDDP cells (Fig. 4M). Furthermore, correlation analysis was performed between circPLPP4 and miR-136 expression levels and PIK3R1 protein levels in 25 OC tissue samples (Fig. 4N, top), Pearson R was used to analyze the correlation between the indicated group (Fig. 4N, bottom).

**Fig. 5:**
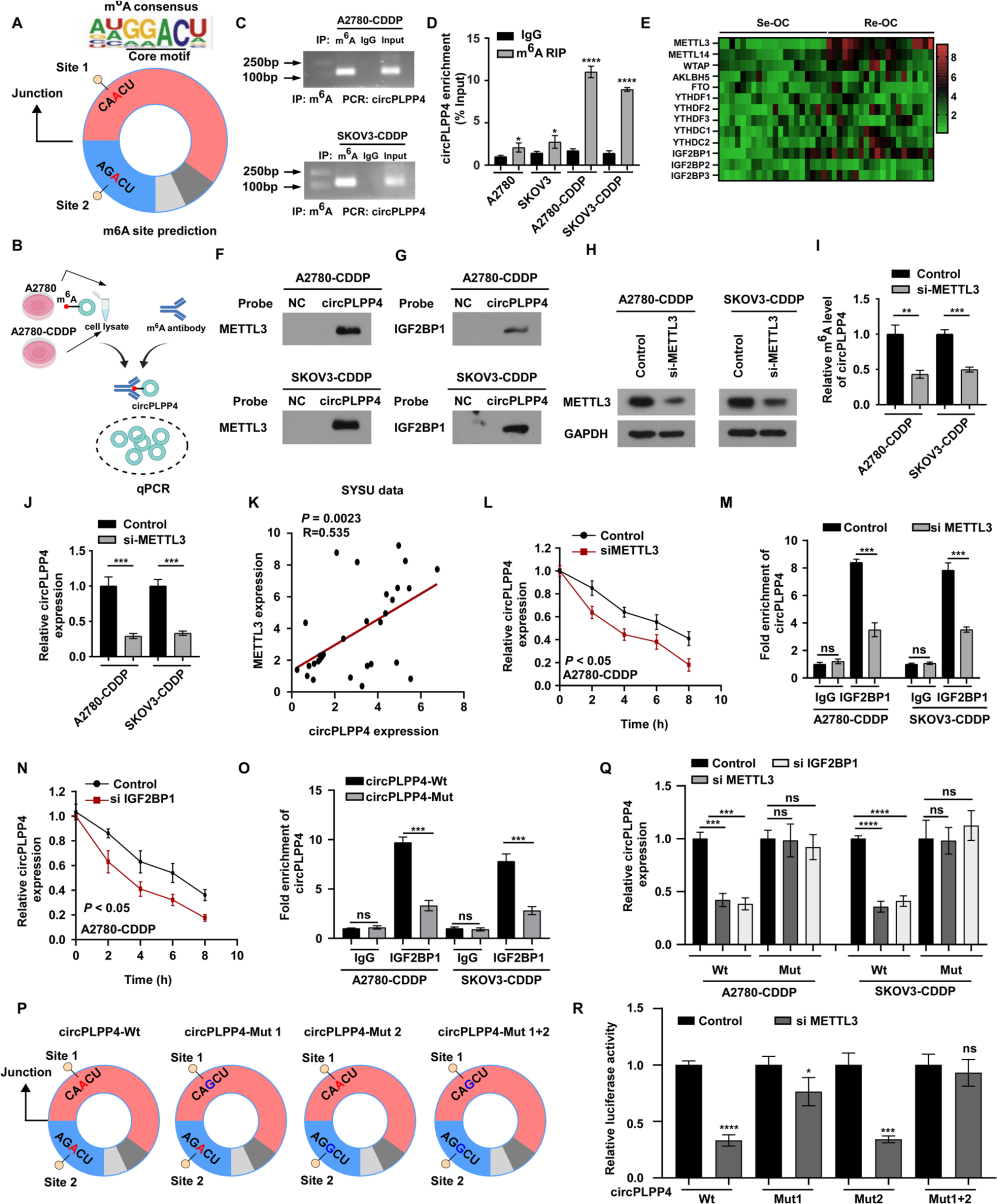
circPLPP4 is modulated by m^6^A methylation. **A** Predicted m^6^A sites in *circPLPP4* from a N^6^-methyladenosine (m^6^A) modification predictor (SRAMP), which based on sequence. **B** Flow diagram of m^6^A-specific immunoprecipitation (MeRIP) assays. **C** MeRIP assays for detecting m^6^A-modified circPLPP4 in A2780 CDDP cells and SKOV3 CDDP cells. **D** m^6^A RIP-qPCR analysis of circPLPP4 in A2780, SKOV3, A2780CDDP and SKOV3CDDP cells. Error bars represent the mean ± SD of three experiments. **E** Heat map profiling the expression of m^6^A WERs in 40 OC tissues (including 20 platinum resistant OC tissues and 20 platinum sensitive OC tissues). **F**, **G** METTL3 and IGF2BP1 were examined by RNA pulldown assays and western blotting using *circPLPP4* probe. **H** METTL3 expression was evaluated by western blotting in the indicated OC cells. GAPDH acted as the loading control. **I** m^6^A RIP-qPCR analysis of the m^6^A level in *circPLPP4* in the indicated cells. Error bars represent the mean ± SD of triplicate experiments. **J** qRT-PCR analysis of *circPLPP4* expression in the indicated cells. Error bars represent the mean ± SD of triplicate. **K** Correlation analysis demonstrated the correlation between *METTL3* and *circPLPP4* in OC tissues obtained from SYSUCC. Statistical analyses were performed by Spearman correlation coefficient. **L**, **N** Control or *METTL3*-knockdown or *IGF2BP1*-knockdown A2780CDDP cells were treated with actinomycin D (5 mg/mL) for the indicated times. Total RNA was extracted and then analyzed using RT-qPCR to assess the half-lives of *circPLPP4*. Error bars represent the mean ± SD of three experiment. **M**, **P** RIP analysis indicating that the enrichment of *circPLPP4* on *IGF2BP1* in the indicated cells. Error bars represent the mean ± SD of triplicate experiment. **P** The putative wild- type m6A sites and designed mutant m6A sites in circPLPP4. **Q** qRT-PCR analysis of *circPLPP4* expression in the *circPLPP4* Wt or circPLPP4-Mut A2780CDDP cells with or without METTL3 or IGF2BP1 silencing. Error bars represent the mean ± SD of triplicate experiments. **R** The luciferase activities of different mutated *circPLPP4* reporter in the indicated groups. Error bars represent the mean ± SD of three experiments. * *P* < 0.05, ** *P* < 0.01, *** *P* < 0.001, **** *P* < 0.0001, ns indicates no significance

**Fig. 6:**
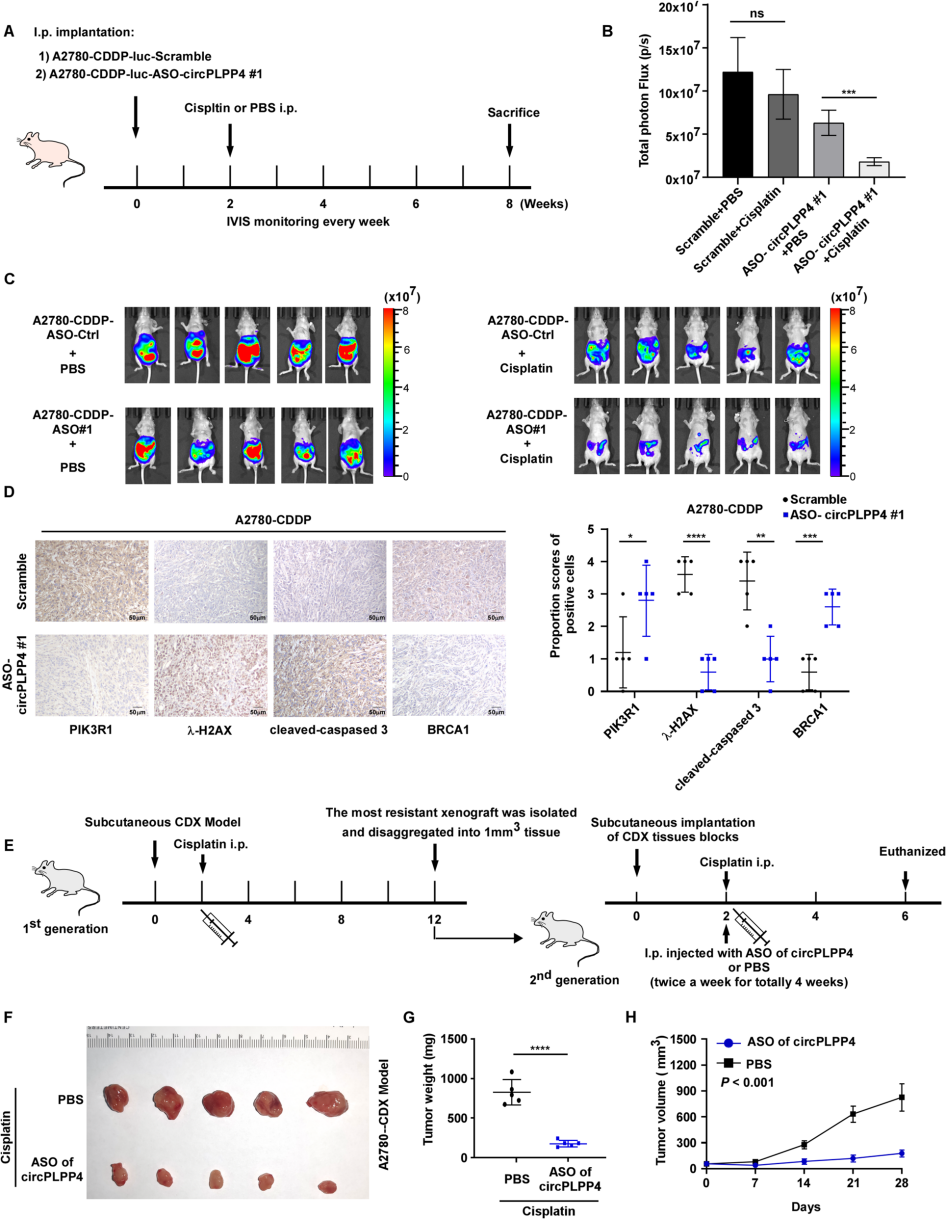
Targeting circPLPP4 in vivo retards CDDP resistant OC. **A**-**C** In vivo luminescent imaging of intraperitoneally implanted A2780 CDDP -luci or A2780 CDDP-luci-ASO-*circPLPP4#*1 cells in 4–6 weeks-old female BALB/c nude mice treated with PBS or CDDP (5 mg/kg) for three times per week upon the luminescence signal reached 2 × 10^7^ p/sec/cm^2^/sr. Luminescence intensity ranges from low (blue) to high (red). Tumor burdens were quantified by total photon flux (p/s). **D** PIK3R1, γH2AX, cleaved caspase-3 and BRCA1 expression levels are examined in representative xenograft tumors by IHC (Left). Quantification of the IHC scores of PIK3R1, γH2AX, cleaved caspase-3 and BRCA1 expression levels (Right). **E**–**H** Flow Chart of A2780-CDX-CR (CDDP resistant) model construction. The first CDX generation was constructed in 4–6 weeks-old female BALB/c nude mice and treated with CDDP (5 mg/kg, three times per week). Twelve weeks later, the most resistant xenograft was disaggregated and implanted subcutaneously into 4–6 weeks-old female BALB/c nude mice as the second CR -CDX. Four weeks after implantation, the second CR -CDX mice were treated with CDDP (5 mg/kg, three times per week) and injected via tail vein with ASOs-targetting *circPLPP4* or its negative control twice a week. Mice were euthanized when the experiments were finished. The subcutaneous tumor size was measured and recorded every 3 days using the Vernier caliper as follows: tumor volume (mm^3^) = (L × W^2^)/2, where L is the long axis and W the short axis. * *P* < 0.05, ** *P* < 0.01, *** *P* < 0.001, **** *P* < 0.0001, ns indicates no significance

These results indicated that *circPLPP4* functions as a competing endogenous RNA (ceRNA) via targeting miR-136 to regulate *PIK3R1* expression and thus promote CDDP resistance in OC.

### ***circPLPP4*** is modulated by m^6^A methylation

We showed that circPLPP4 is likely formed from pre-mRNA back-splicing of exons 2–4 of the PLPP4 transcript. However, the mechanism controlling circPLPP4 generation is unclear. Recently studies suggested that epigenetic mechanisms are frequently involved in the dysregulation of noncoding RNAs; therefore, we wondered whether epigenetic regulation is responsible for circPLPP4 upregulation in OC cisplatin resistance. Firstly, treatment of OC cells with a DNA methyltransferase inhibitor had no effect on circPLPP4 expression (Supplemental Fig. 7A), suggesting that DNA methylation does not have a major role in circPLPP4 regulation. Next, we investigated whether histone acetylation participates in circPLPP4 regulation. Treatment of OC cell with broad-spectrum HDAC inhibitors (SAHA and NaB) did not affect circPLPP4 expression (Supplemental Fig. 7B), indicating that histone acetylation modification does not participate in circPLPP4 regulation.

**Fig. 7:**
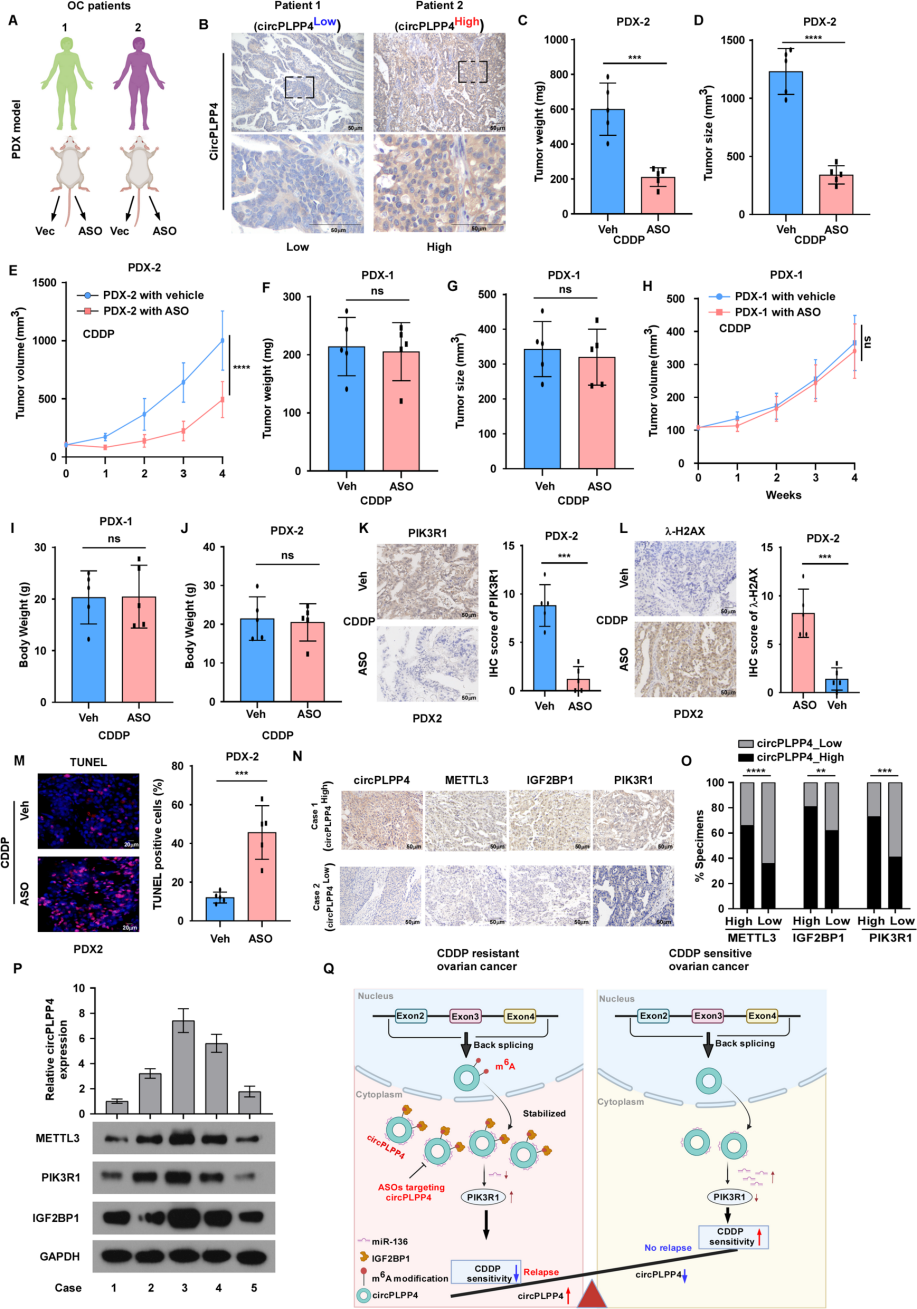
circPLPP4 acts as a therapy target in EOC pre-clinical models. **A** Schematic treatment administration (in vivo-optimized *circPLPP4* inhibitor or control) in PDX models. **B** Representative images of *circPLPP4* ISH analysis of OC tissue samples from OC patients. **C**, **D** Tumor weight and volume were examined in PDX-2 after 4-week treatment. **E** Tumor volumes were measured at the indicated time points in PDX-2. **F**, **G** Tumor weight and volume were assessed in PDX-1 after 4-week treatment. **H** Tumor volumes were measured at the indicated time points in PDX-1. **I**, **J** Body weights of tumor-bearing mice (PDX-1 and PDX-2) treated with CDDP combined with in vivo-optimized *circPLPP4* inhibitor or control. **K**-**M** Decreased PIK3R1 (**K**), increased γ-H2AX level (**L**) and apoptosis (**M**) were showed in PDX-2 tumors after CDDP combined with in vivo-optimized *circPLPP4* inhibitor treatment. Left, representative images of indicated staining. Right, quantification result according to its corresponding criteria . **N** Representative images showing high or low expression of *circPLPP4*, METTL3, IGF2BP1 and PIK3R1 in OC tumor specimens. **O** Correlation between *circPLPP4* and METTL3 IGF2BP1or PIK3R1 in 166 OC tumor specimens. **P** qRT-PCR and western blotting analysis of *circPLPP4* and METTL3, IGF2BP1 or PIK3R1 expression in five OC tumor specimens. *circPLPP4* levels were normalized to that *circPLPP4* expression of case 1. GAPDH was acted as loading controls. **Q** Graphical abstract for *circPLPP4* function in CDDP resistance in OC. METTL3-mediated m^6^A modification for *circPLPP4* acts as a sponge for miR-136 to promote OC CDDP resistance via regulating PIK3R1 signaling. * *P* < 0.05, ** *P* < 0.01, *** *P* < 0.001, **** *P* < 0.0001, ns indicates no significance

Studies suggest that m^6^A is the most prevalent modification of mRNAs and noncoding RNAs; therefore, it might have an important role in circRNA biogenesis [25, 26]. The m^6^A modification occurs mainly on the consensus motif ‘RRm^6^ACH’ (R = G or A; H = A, C or U) [27]. SRAMP algorithm [28] predicted an m^6^A modification site close to the junction region of circPLPP4 (Fig. 5A). m^6^A-specific immunoprecipitation assays demonstrated an increased m^6^A level on circPLPP4 in A2780 CDDP and SKOV3 CDDP cells compared with that in their parental cells (Fig. 5B-D), suggesting that m^6^A modification might be involved in circPLPP4 upregulation. To further identify the molecule that induces m^6^A modification of circPLPP4, we examined m^6^A-related gene expression in OC in our OC cohort (20 platinum resistant OC tissues and 20 platinum sensitive OC tissues; 20 OC tissues and 20 normal ovary tissues) using qRT-PCR. The results showed that several m^6^A-related genes were dysregulated in OC (Fig. 5E, Supplemental Fig. 7C). We validated that *METTL3* was markedly upregulated in OC tissues; whereas, we found no significant difference among other m^6^A-related genes (Supplemental Fig. 7C). Notably, an RNA pulldown assay revealed that circPLPP4 interacts with the key m^6^A methyltransferase, METTL3, and a classical m^6^A reader, IGF2BP1 (Fig. 5F, G). These results encouraged us to explore the role of METTL3 in regulating m^6^A modification of circPLPP4. We treated A2780 CDDP and SKOV3 CDDP cells with an siRNA targeting *METTL3*, which showed that *METTL3* knockdown markedly decreased the m^6^A level and the level of circPLPP4 (Fig. 5H-J). Interestingly, *METTL3* and circPLPP4 expression were positively associated in our OC cohort (Fig. 5K), indicating that positive regulation by METTL3 on circPLPP4. Additionally, the circPLPP4 level was reduced when ALKBH5 (an N6-demethylase) was overexpressed (Supplemental Fig. 7D, E). These results suggested that the m^6^A modification upregulates the circPLPP4 level. Next, explored the detailed mechanism of m^6^A-mediated upregulation of circPLPP4 in OC cells. Silencing of *METTL3* affect the stability and decreased the half-life of circPLPP4 significantly (Fig. 5L), suggesting that METTL3 regulates the circPLPP4 level by modulating its stability. The m^6^A-mediated regulation of circPLPP4 needs an m^6^A reader to recognize the m^6^A modification, we investigated which m^6^A reader regulates circPLPP4. An RIP assay showed that circPLPP4 was markedly immunoprecipitated by IGF2BP1 rather than other m^6^A readers (Fig. 5M, Supplemental Fig. 7F). Moreover, IGF2BP1 inhibition in OC cells decreased the level and half-life of circPLPP4 significantly (Fig. 5N, Supplemental Fig. 7G). A nucleus-cytoplasm fractionation analysis suggested that *METTL3* silencing did not affect the localization of circPLPP4 in OC cells (Supplemental Fig. 7H). Furthermore, when we mutated the putative m^6^A site in circPLPP4 (Fig. 5O), the direct binding between circPLPP4 and IGF2BP1 was impaired, as assessed using an RIP assay (Fig. 5P). Moreover, METTL3- or IGF2BP1-induced regulation of circPLPP4 was abolished when the m^6^A sites were mutated (Fig. 5Q). In addition, METTL3 or IGF2BP1-mediated m^6^A modification of circPLPP4 was repressed upon mutation of both m^6^A sites (Fig. 5R).

### Targeting circPLPP4 in vivo retards CDDP-resistant OC

To further verify the *in vitro* results and to discover potential clinical therapy targets, we constructed intraperitoneal implantation models to explore the role of circPLPP4 in CDDP resistance *in vivo*. Intraperitoneal implantation of A2780 CDDP cells transfected with ASO#1 resulted in tumors that were significantly more sensitive to cisplatin than those formed from the control cells, while the two groups showed a similar response to PBS treatment (Fig. 6A-C). IHC analysis and western blot analysis of tumor xenograft samples also showed that the levels of γH2AX and cleaved caspase-3 were obviously upregulated, whereas PIK3R1 and BRCA1 levels were decreased upon circPLPP4 inhibition (Fig. 6D, Supplemental Fig. 8A). Moreover, tail vein injection of the *in vivo*-optimized circPLPP4 inhibitor (three dose of ASOs as follows: the ASO-H group, 10 nmol in 100 μl PBS per mouse injection; the ASO-L1 group, 2 nmol in 100 μl PBS for each mouse; the ASO-L2 group, 5 nmol in 100 μl PBS for each mouse) in the both A2780-CDX and SKOV3-CDX models obviously enhanced the sensitivity of OC cells to CDDP treatment (Fig. 6E-H, Supplemental Fig. 9A-F).

### *circPLPP4* is a therapeutic target in OC pre-clinical models

Next, we assessed the therapy efficacy of the *in vivo*-optimized circPLPP4 inhibitor (circPLPP4-ASO#1) in three OC Patient-derivedxenografts (PDX) models (Fig. 7A). Based on the ISH scores of patient tumor sections, one PDX (named as PDX-1) was defined as a circPLPP4^Low^ patient, while PDX-2 and PDX-3 were regarded as a circPLPP4^High^ patient (Fig. 7B, Supplemental Fig. 10A). When the circPLPP4-ASO#1 was used in the both PDX-2 and PDX-3 model, tumor growth was inhibited significantly compared with that in the controls (Fig. 7C-E, Supplemental Fig. 10B-D). As expected, circPLPP4-ASO#1 failed to repress tumor growth in the PDX-1 model (Fig. 7F-H). In addition, mouse body weights showed no significant change the PDX-1, PDX-2 and PDX-3 models, with or without circPLPP4-ASO#1 treatment (Fig. 7I, J, Supplemental Fig. 10E). An IHC assay indicated that PIK3R1 levels were decreased and γ-H2AX levels were increased, accompanied by tumor growth repression, after circPLPP4-ASO#1 application in the PDX-2 model (Fig. 7K, L). Moreover, the apoptotic response (TUNEL positive cells) increased markedly in the PDX-2 models treated with circPLPP4-ASO#1 (Fig. 7M). These results suggested that circPLPP4 is a promising therapeutic target.

### Clinical relevance of the m^6^A/***circPLPP4***/PIK3R1 axis in OC

To further explore the clinical relevance of the m^6^A/circPLPP4/PIK3R1 axis in OC, we detected the expression of circPLPP4, METTL3, IGF2BP1, and PIK3R1 in OC tissues from the SYSUCC cohort via ISH and IHC assays. The results revealed that circPLPP4 was positively associated with METTL3, IGF2BP1, and PIK3R1 expression (Fig. 7N, O). Moreover, correlation analysis indicated a positive association between circPLPP4 expression and METTL3, IGF2BP1, or PIK3R1 expression, as detected using qRT-PCR (Supplemental Fig. 11A-C). Notably, western blotting and qRT-PCR analysis confirmed the positive association between *circPLPP4* expression and METTL3, IGF2BP1, or PIK3R1 in five OC tissues (Fig. 7P). In summary, METTL3-mediated m^6^A modification increases circPLPP4 level in an IGF2BP1-recognized manner, which activates the circPLPP4/miR-136/PIK3R1 axis, which subsequently contributes to CDDP resistance in OC (Fig. 7Q).

## Discussion

CDDP treatment is one of the first line chemotherapeutic strategies for patients with OC. CDDP resistance frequently induces poor prognosis and a decline of life quality of patients with OC [3, 29]. Thus, exploring the precise mechanisms underlying CDDP resistance and developing novel therapeutic targets are essential. Studies have demonstrated that circRNAs are involved in tumor development [9, 11–13]. However, the biological function and mechanism of circRNAs in OC CDDP resistance is unclear. In this study, we conducted next-generation sequencing to discover differentially expressed circRNAs between OC tissues with CDDP resistance and CDDP sensitive OC tissues. We selected circPLPP4, an overexpressed circRNA with a high-fold change and a significant *P* value in the CDDP resistant OC tissues. CircPLPP4 was upregulated in OC with CDDP resistance and predicted poor prognosis. Moreover, circPLPP4 knockdown markedly inhibited CDDP resistance of OC cells *in vitro and in vivo,* as well as in the PDX model. Mechanistically, m^6^A-modified circPLPP4 is more stable and thus shows an apparently increased level. Moreover, circPLPP4 acts as a microRNA sponge to sequester miR-136, thus competitively upregulating PIK3R1 expression and conferring CDDP resistance. The “circPLAPP4- miR-136- PIK3R1” axis as a regulatory whole co-regulates CDDP resistance in ovarian cancer.

NcRNAs participate in numerous cellular biological processes and pathological disease, especially in cancer [15]. CircRNAs exert their crucial roles by acting as ceRNAs to regulate downstream genes; as scaffolds to promote protein degradation, decreased the stability of mRNA, or induce related gene transcription; or interact with RBPs to regulate other gene expression [30–33]. Functioning as a ceRNA is the most frequently reported method. For example, *circSETD3* inhibits the growth of hepatocellular carcinoma via the circSETD3/miR-421/MAPK14 axis [34]. *CircPSMC3* functions as an miR-136 sponge to increase *PTEN* (phosphatase and tensin homolog) expression, and further suppresses the proliferation and metastasis of gastric cancer [35]. Here, we showed that circPLPP4 promotes PIK3R1 expression in OC. Moreover, circPLPP4 and PIK3R1 share microRNA response elements (MREs) for miR-136, implying the formation of a circPLPP4/miR-136/PIK3R1 axis. Luciferase reporter and RIP assays verified the direct interaction between circPLPP4 and miR-136. Moreover, loss and gain function assays and luciferase reporter assays indicated that miR-136 inhibits PIK3R1 translation directly by binding to the 3′-UTR of PIK3R1 mRNA. We detected that miR-136 was decreased in CDDP resistant OC tissues, was negatively associated with clinicopathological features and unfavorable prognosis, and inhibited the CDDP resistance of OC. These findings suggested that miR-136 might function as a tumor suppressor. Furthermore, miR-136 overexpression inhibited the ability of circPLPP4 to promote *PIK3R1* expression and CDDP resistance, while overexpression of PIK3R1 reversed the ability of miR-136 to suppress CDDP resistance. Therefore, we concluded that circPLPP4 enhances the CDDP resistance of OC cells via acting as an miR-136 sponge and relieving its abolition of PIK3R1 expression.

PIK3R1 encodes PI3K regulatory subunit (named as p85α) [10]. Several studies have reported that the p85α regulatory subunit activates PI3K signaling in CDDP resistance. PI3K signaling is pivotal in tumor progression, and its inhibition is regarded as a valuable strategy for cancer therapy [36]. PI3K signaling is also involved in platinum resistance [37]. For example, CDDP-induced apoptosis related pathway inhibition is mediated by AKT activation [37]. Moreover, PI3K activation caused the BRCA1 overexpression and re-sensitized cells to CDDP treatment in breast cancer [38]. However, the underlying mechanism by which PIK3R1 confers resistance to CDDP in OC remains to be explored.

M^6^A is the most prevalent posttranscriptional modification of RNAs, including circRNAs, which mediates RNA metabolism at numerous levels [36]. m^6^A modification is mediated by m^6^A writers (including METTL3, METTL14, KIAA1429, WTAP, RBM15 and ZC3H13), readers (containing YTHDC1, YTHDC2, YTHDF1, YTHDF2 and HNRNPC), and erasers (FTO and ALKBH5) [39]. Research suggests that m^6^A modification of RNAs have important functions in cancer progression [27, 40]. m^6^A modification of *CircE7*, an oncoprotein-encoding circRNA, significantly promoted tumor growth of cervical cancer in vitro and in vivo. Notably, m^6^A modification is essential for the protein-coding ability of *CircE7* [41]. In addition, m^6^A modification influences the function of circRNA by regulating the methylation state of downstream genes. For instance, YAP is the main effector of Hippo signaling, and is associated significantly with the development of numerous tumors. m^6^A modification of YAP causes an interaction with miR-382-5p, inhibiting YAP expression [42]. Similarly, the promoting effect of circRNA_104075 is repressed inn hepatocellular carcinoma [43]. However, the roles of m^6^A -modified circRNAs in OC and CDDP resistance were unknown. Our data showed that the m^6^A modification increased the level of *circPLPP4* in CDDP resistant cells and tissues, and *circPLPP4* levels decreased upon repression of m^6^A modification.

Studies have shown that ncRNAs are potential therapeutic targets to treat cancers [44]. Antisense technology has developed as a novel and promising therapeutic strategy in cancer. A series of key regulatory genes involved in pathological processes, including proliferation, metastasis, invasion, and angiogenesis, are regarded as design targets for antisense therapy [45]. Recently, ASOs have been developed that can hybridize to the complementary RNA to form a DNA-RNA hetero-duplex that promotes RNase H-induced RNA degradation [45]. For example, ASOs targeting *MALAT1* accompanied by nanostructure system, were used to inhibit cancer metastasis [46]. In addition, an ASO targeting long non-coding RNA *TUG1* inhibit the progression of glioma significantly [16]. Moreover, clinical trials have begun to investigate the efficacy of ASO drugs. ASO drugs targeting *BCL2* induced apoptosis in solid tumor cells and increased the sensitivity of tumor cells to chemotherapy [47]. AZD9150, an ASO drug targeting *STAT3*, was validated as effective in patients with diffuse large B-cell lymphoma assessed in a phase 1b clinical trial [48]. In the present study, we found that *circPLPP4* inhibition by an ASO suppressed CDDP resistance in OC, suggesting the application of small molecules targeting circPLPP4 as a novel therapeutic strategy against CDDP resistance in OC.

## Conclusion

Taken together, through a human circRNAs-sequencing assay and bioinformatics analyses, we discover that circPLPP4 is significantly upregulated in cisplatin resistant ovarian cancer tissues and that this high circPLPP4 expression promoted ovarian cancer platinum resistance through miR-136/ PIK3R1 signaling axis. The m^6^A modification of circPLPP4 promoted the binding ability of circPLPP4 to miR-136 and further enhanced PIK3R1 expression (Fig. 7Q). Based on our findings, targeting of circPLPP4 could represent a valuable therapeutic target for OC patients with platinum resistance.

### Supplementary Information

**Additional file 1: Table 1.** Clinicopathological characteristics and expression of circPLPP4 in ovarian cancer.

**Additional file 2: Table 2.** Correlation between circPLPP4 expression and the clinicopathological features of ovarian cancer.

**Additional file 3: Table 3.** Cox regression univariate and multivariate analyses of prognostic factors in ovarian cancer

**Additional file 4: Table S4.** qRT-PCR primers

**Additional file 5: Supplementary Table 5.** siRNA, ASO & shRNA sequence.

**Additional file 6: Supplemental Figure 1.** CircPLPP4 expression level is relevant with poor prognosis in OC patients. (A) .qRT-PCR analysis of *PLPP4* mRNA expression in a 20-case cohort of freshly collected human OC samples with Platinum resistance and 20-case cohort of Platinum sensitive OC samples. (B). Correlation analysis between *circPLPP4* expression and patient vital status. (C). Kaplan–Meier analysis of Overall survival (OS) in OC patients stratifed by low and high *circPLPP4* levels (*n*= 166, log-rank test). HR, hazard ratio. * *P* < 0.05, ** *P* < 0.01, *** *P* < 0.001, **** *P* < 0.0001, ns indicates no significance.

**Additional file 7: Supplemental Figure 2.** (A) RT-qPCR analysis of *circPLPP4* expression in the eight paired cisplatin-resistant ovarian cancer biopsies obtained before and after platinum-based chemotherapy. (B) RT-qPCR analysis of *circPLPP4* expression in the eight paired cisplatin-sensitive ovarian cancer biopsies obtained before and after platinum-based chemotherapy.* *P* < 0.05, ** *P* < 0.01, *** *P* < 0.001, **** *P* < 0.0001, ns indicates no significance. Each error bar represents the mean ± SD of three independent experiments.

**Additional file 8: Supplemental Figure 3.** circPLPP4 knockdown increased CDDP sensitivity of CDDP-resistant OC cells. (A) RT-qPCR analysis of *circPLPP4* and PLPP4 expression in the ASO-ctrl or *circPLPP4*-ASO#1, *circPLPP4*-ASO#2 cells. (B) MTT cell viability assay of CDDP in the indicated OC cells. (C, D) Quantification of colony number of the indicated cells. (E) FACS analysis of Annexin V/PI staining of indicated cells treated with vehicle or CDDP (5μM) after 24 hours. (F) Western blotting analysis of level of caspased 3, cleaved-caspased 3, PARP and cleaved-PARP in the indicated cells. (G) Western blotting analysis of level of γ-H2AX and BRCA1 in the indicated cells. (H) Immunofluorescence staining and quantification of of γ-H2AX in the indicated cells treated with CDDP (5μM) after 24 hours. * *P* < 0.05, ** *P* < 0.01, *** *P* < 0.001, **** *P* < 0.0001, ns indicates no significance. Each error bar represents the mean ± SD of three independent experiments.

**Additional file 9: Supplemental Figure 4.** circPLPP4 confers CDDP resisitance of CDDP in OC cells. (A) qRT-PCR analysis of circPLPP4 and PLPP4 expression in the indicated OC cells. (B, C) MTT cell viability assay of CDDP in the indicated cells. (D, E) Quantification of colony number of the indicated cells. (F) Western blotting analysis of level of caspased 3, cleaved-caspased 3, PARP and cleaved-PARP in the indicated cells. (G) Western blotting analysis of level of γ-H2AX and BRCA1 in the indicated cells. (H, I) FACS analysis of Annexin V/PI staining of indicated cells with vehicle or CDDP (5μM) treatment after 24 hours. (J, K) Quantification of γ-H2AX in the indicated OC cells with CDDP (5μM) treatment after 24 hours. * *P* < 0.05, ** *P* < 0.01, *** *P* < 0.001, **** *P* < 0.0001, ns indicates no significance. Each error bar represents the mean ± SD of three independent experiments.

**Additional file 10: Supplemental Figure 5.** (A-C) The expression of SLAMF7, UGT1A4 and PRDM8 were analyzed using qRT-PCR in A2780 CDDP, SKOV3 CDDP, A2780 and SKOV3 cells. Error bars represent the mean ± SD of three experiments. Statistical analyses were performed by unpaired Student’s t-test. * *P* < 0.05, ** *P* < 0.01, *** *P* < 0.001, **** *P* < 0.0001, ns indicates no significance. Each error bar represents the mean ± SD of three independent experiments.

**Additional file 11: Supplemental Figure 6.** circPLPP4 enhanced PIK3R1 expression by sponging miR-136 in OC cells. (A) The expression levels of PIK3R1 were examined using qRT-PCR in the indicated cells. (B) The expression levels of PIK3R1 were analyzed using qRT-PCR. SKOV3 cells were transfected with the indicated vectors and miR-136 mimics. (C) The expression levels of PIK3R1 were analyzed using qRT-PCR in the indicated cells. (D, E). The proteins levels of PIK3R1, apoptosis markers, γH2AX, BRCA1 were detected using western blotting in A2780 and SKOV3 cells transfected with the indicated vectors and miR-136 mimics after CDDP treatment (5μM). (F) The IC50 was detected by the MTT assay. SKOV3 CDDP cells were transfected with miR-136 mimic alone or co-transfected with the indicated vectors upon CDDP exposure (5 μM) for 48 h. (G) The apoptosis rates of SKOV3CDDP cells transfected with miR-136 mimic alone or co-transfected with the indicated vectors upon CDDP treatment (5 μM) for 48 h. The results are presented as the mean ± SEM. * *P* < 0.05, ** *P* < 0.01, *** *P* < 0.001, **** *P* < 0.0001, ns indicates no significance. Each error bar represents the mean ± SD of three independent experiments.

**Additional file 12: Supplemental Figure 7.** m6A modification contributes to the upregulation of circPLPP4 in OC. (A) qRT-PCR analysis of circPLPP4 expression in the indicated cells with or without treatment of 5-zaz-dC. (B) RT-qPCR analysis of circPLPP4 expression in the indicated cells with or without treatment of SAHA or NaB. (C) qRT-PCR analysis of mRNA expression in 20 OC tissues and 20 normal ovary tissues. (D) Western blotting of ALKBH5 expression in the indicated cells. GAPDH served as the loading control. (E). qRT-PCR analysis of circPLPP4 in the indicated cells. (F) RIP analysis showing the enrichment of circPLPP4 on several proteins in the indicated cells. (G) qRT-PCR analysis of circPLPP4 expression in the indicated cells. (H) Nuclear–cytoplasmic fractionation assays revealing circPLPP4 expression in cytoplasm and nucleus of control or METTL3-knockdown the indicated OC cells. U3 and GAPDH were used as positive controls in the nucleus and cytoplasm, respectively. * *P* < 0.05,** *P* < 0.01, *** *P* < 0.001, **** *P* < 0.0001, ns indicates no significance. Each error bar represents the mean ± SD of three independent experiments.

**Additional file 13: Supplemental Figure 8.** (A) Western blotting of PIK3R1, γ-H2AX, caspase 3, cleaved caspase 3 and BRCA1 expression in the indicated A2780-CDDP tumor. GAPDH served as the loading control.

**Additional file 14: Supplemental Figure 9.** (A) representative images of tumors with different treatment. (B, C)Tumor weight and tumor volume of A2780 CDX model treated with different dose of ASOs or PBS control. *n* = 5 for each group. Tumor volume (mm^3^)= (L × W^2^)/2, where L is the long axis and W the short axis. (D) representative images of tumors with different treatment. (E, F)Tumor weight and tumor volume of SKOV3 CDX model treated with different dose of ASOs or PBS control. *n* = 5 for each group. Tumor volume (mm^3^) = (L × W^2^)/2, where L is the long axis and W the short axis. * *P* < 0.05, ** *P* < 0.01, *** *P* < 0.001, **** *P* < 0.0001, ns indicates no significance.

**Additional file 15: Supplemental Figure 10.** (A) Representative images of *circPLPP4* ISH analysis of OC tissue samples from OC patients. (B, C) Tumor weight and volume were examined in PDX-3 after 4-week treatment. (D) Tumor volumes were measured at the indicated time points in PDX-3. (E) Body weights of tumor-bearing mice (PDX-3) treated with CDDP combined with in vivo-optimized *circPLPP4* inhibitor or control. (F, G) The expression of miR-136 and IGF2BP1 were analyzed using qRT-PCR in three PDX models. Statistical analyses were performed by unpaired Student’s t-test. * *P*< 0.05, ** *P* < 0.01, *** *P* < 0.001, **** *P* < 0.0001, ns indicates no significance. Each error bar represents the mean ± SD of three independent experiments.

**Additional file 16: Supplemental Figure 11.** Clinical relevance of m6A/ circPLPP4 /PIK3R1 axis in OC. (A-C) Correlation analysis showing the correlation between circPLPP4 and METTL3 (A), IGF2BP1 (B) or PIK3R1 (C) in OC specimens. Statistical analyses were performed by Spearman correlation coefficient.

**Additional file 17. **

**Additional file 18. **

## Data Availability

The datasets used during the current study are available from the corresponding author on reasonable request.

## Abbreviations

circRNAs: Circular RNAs
OC: Ovarian cancer
CDDP: Cisplatin
ISH: In situ hybridization
m^6^A: N6-methyladenosine
MeRIP: Methylated RNA immunoprecipitation
ceRNA: Competing endogenous RNA
MREs: MicroRNA response elements
PIK3R1: Phosphoinositide-3-kinase regulatory subunit 1
PTEN: Phosphatase and tensin homolog
PDX: Patient-derived xenografts
RBP: RNA binding protein
AGO2: Argonaute 2
ASOs: Antisense oligonucleotides
UTR: Untranslated region
IF: Immunofluorescence
BSA: Bovine serum albumin
MTT: 3-(4,5-Dimethylthiazol-2-yl)-2,5-diphenyltetrazolium bromide
DMSO: Dimethyl sulphoxide
TUNEL: Terminal deoxynucleotidyl transferase-mediated dUTP Nick end labeling
SD: Standard deviation
