# Supplementary figures and images for: N6-methyladenosine-modified circPLPP4 sustains cisplatin resistance in ovarian cancer cells via PIK3R1 upregulation
Source: Mol Cancer. 2024 Jan 6;23:5. doi: 10.1186/s12943-023-01917-5 (PMC10770956; doi:10.1186/s12943-023-01917-5)

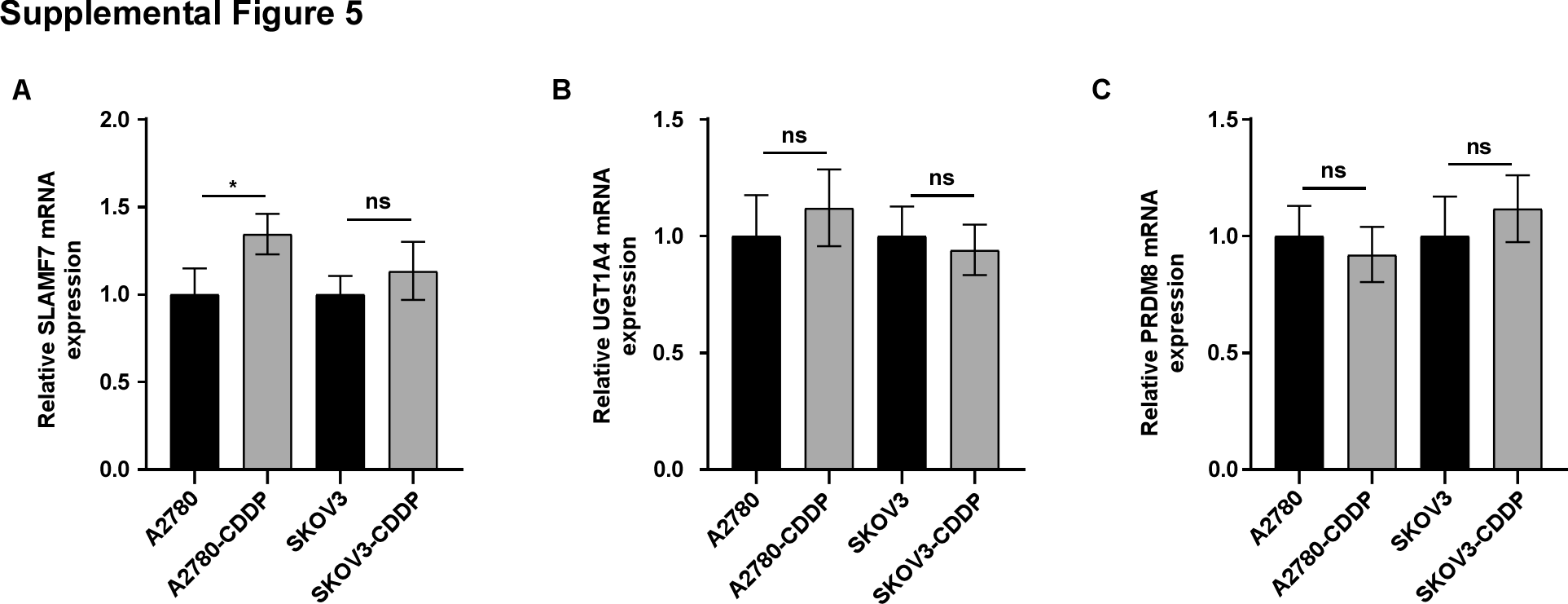

Supplement: Supplementary file 1 — Additional file 1: Table 1. Clinicopathological characteristics and expression of circPLPP4 in ovarian cancer. [file 12943_2023_1917_MOESM1_ESM.tif]

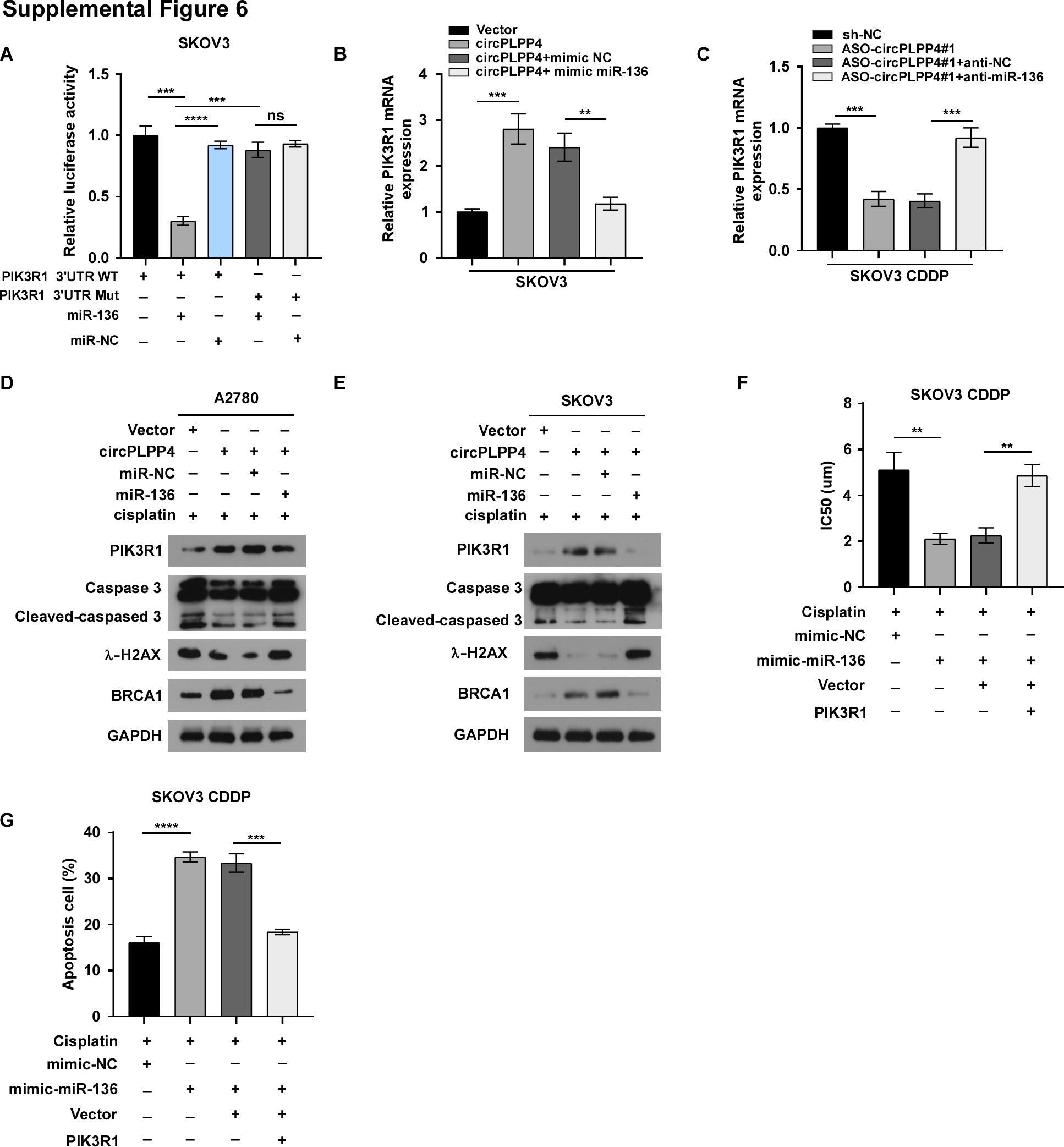

Supplement: Supplementary file 2 — Additional file 2: Table 2. Correlation between circPLPP4 expression and the clinicopathological features of ovarian cancer. [file 12943_2023_1917_MOESM2_ESM.tif]

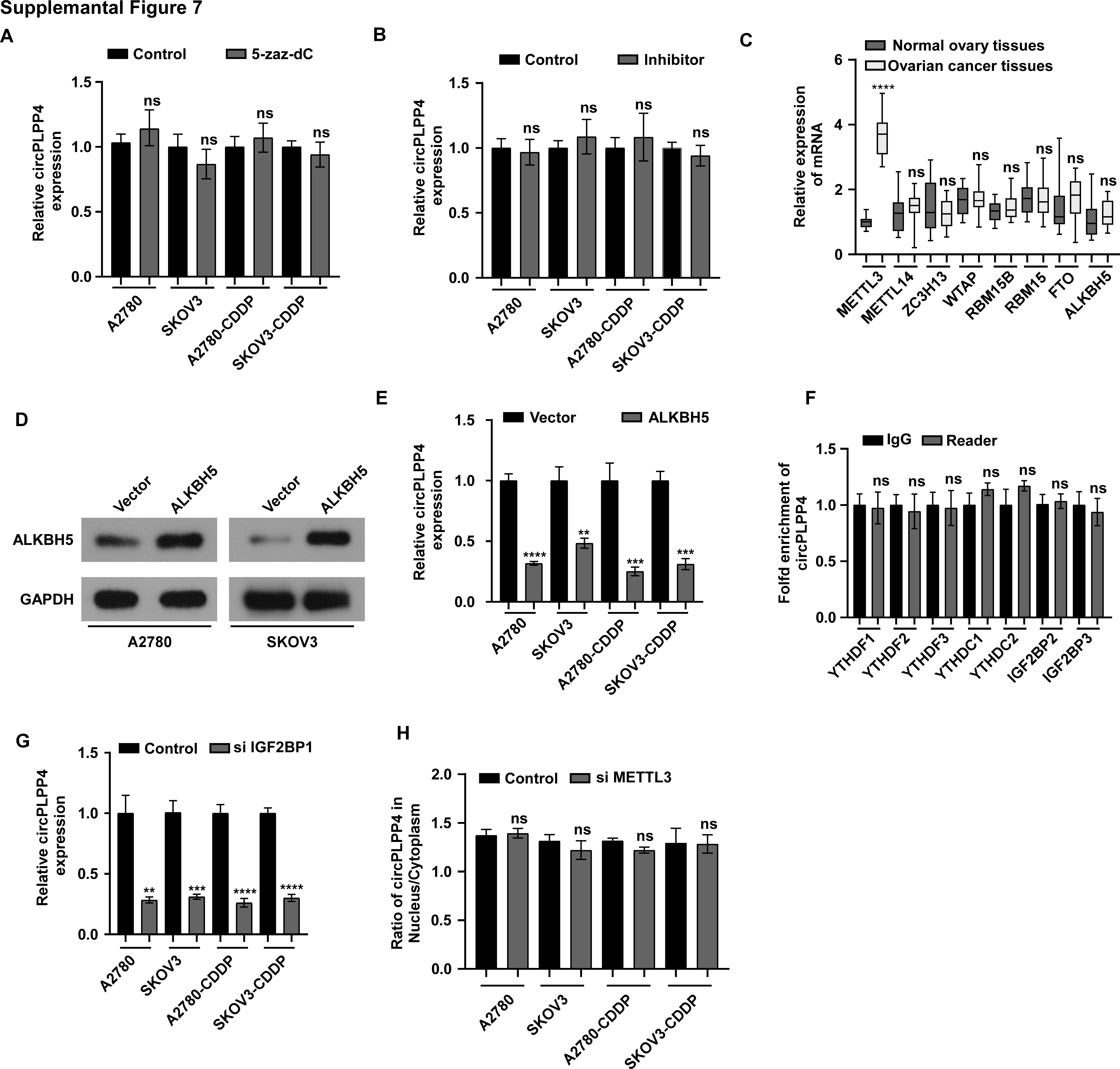

Supplement: Supplementary file 3 — Additional file 3: Table 3. Cox regression univariate and multivariate analyses of prognostic factors in ovarian cancer [file 12943_2023_1917_MOESM3_ESM.tif]

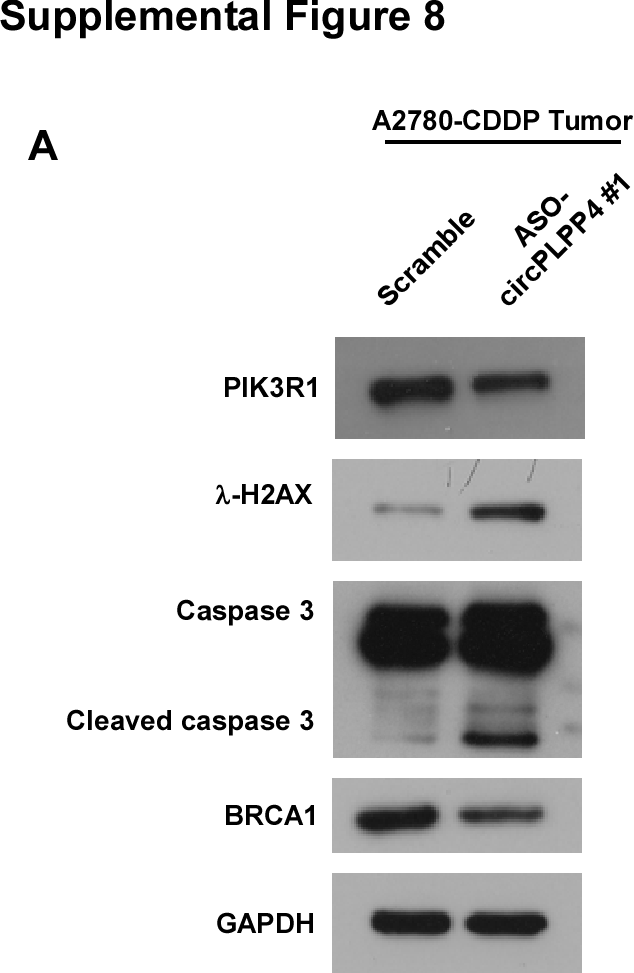

Supplement: Supplementary file 4 — Additional file 4: Table S4. qRT-PCR primers [file 12943_2023_1917_MOESM4_ESM.tif]

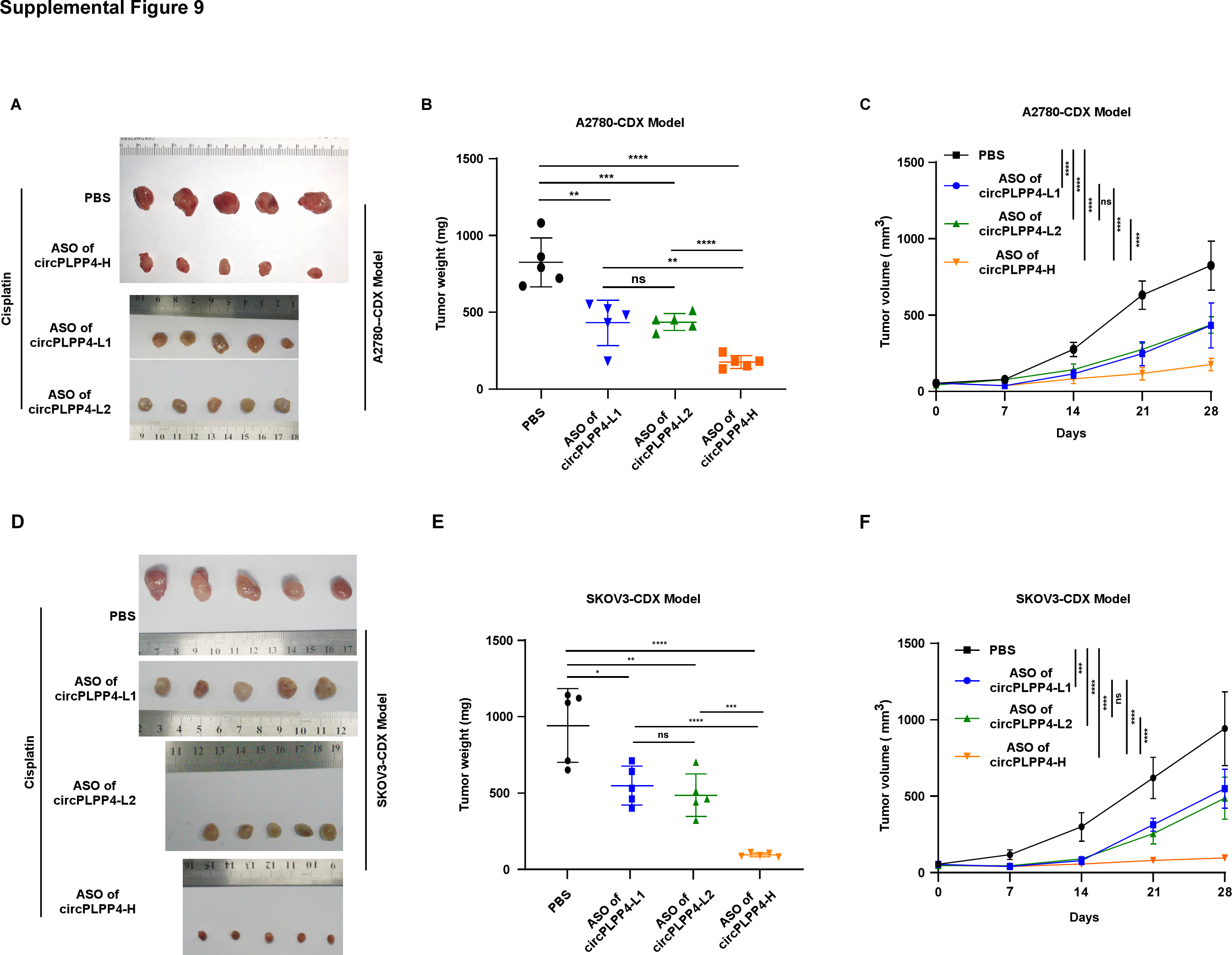

Supplement: Supplementary file 5 — Additional file 5: Supplementary Table 5. siRNA, ASO & shRNA sequence. [file 12943_2023_1917_MOESM5_ESM.tif]

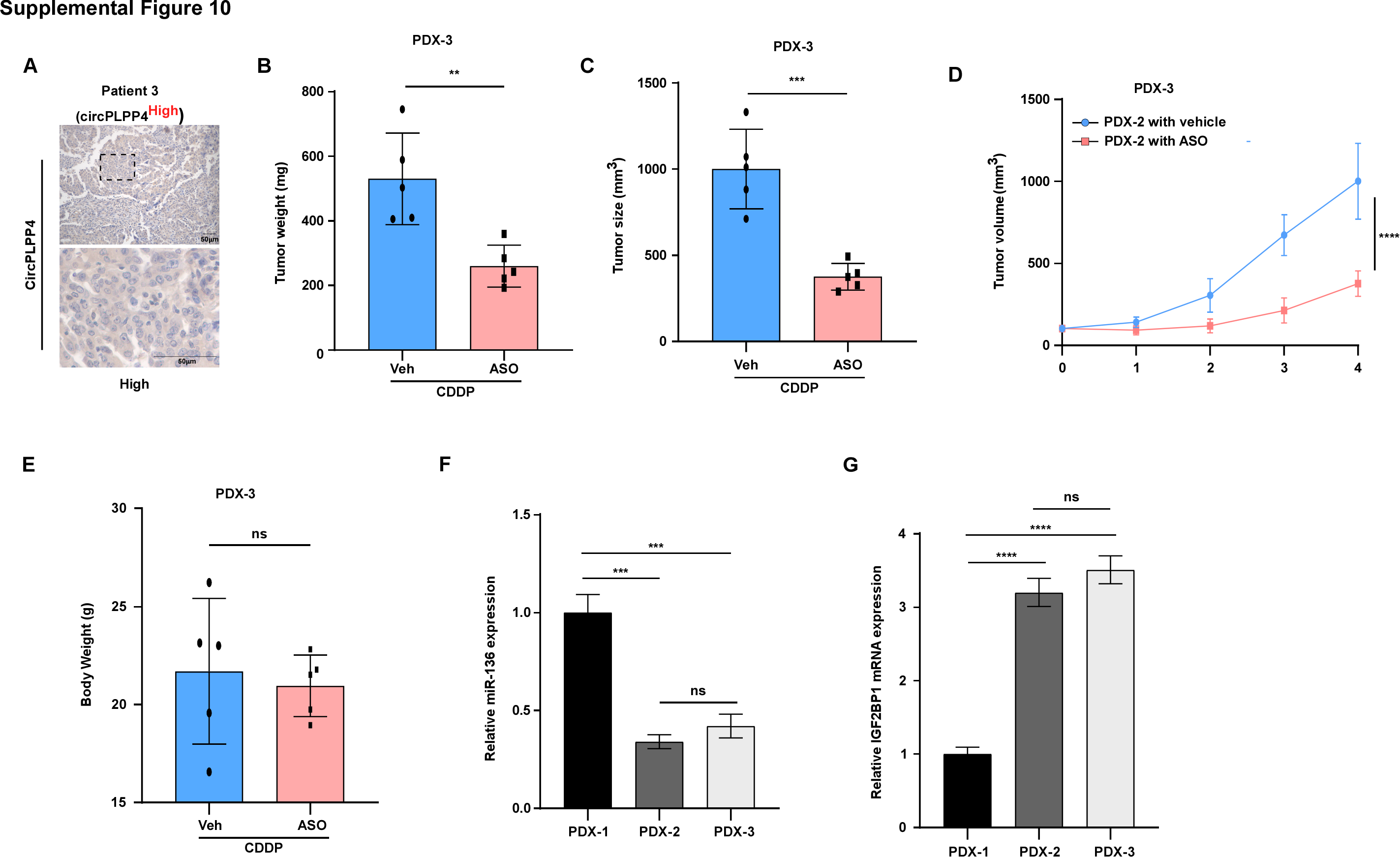

Supplement: Supplementary file 6 — Additional file 6: Supplemental Figure 1. CircPLPP4 expression level is relevant with poor prognosis in OC patients. (A) .qRT-PCR analysis of PLPP4 mRNA expression in a 20-case cohort of freshly collected human OC samples with Platinum resistance and 20-case cohort of Platinum sensitive OC samples. (B). Correlation analysis between circPLPP4 expression and patient vital status. (C). Kaplan–Meier analysis of Overall survival (OS) in OC patients stratifed by low and high circPLPP4 levels (n= 166, log-rank test). HR, hazard ratio. * P < 0.05, ** P < 0.01, *** P < 0.001, **** P < 0.0001, ns indicates no significance. [file 12943_2023_1917_MOESM6_ESM.tif]

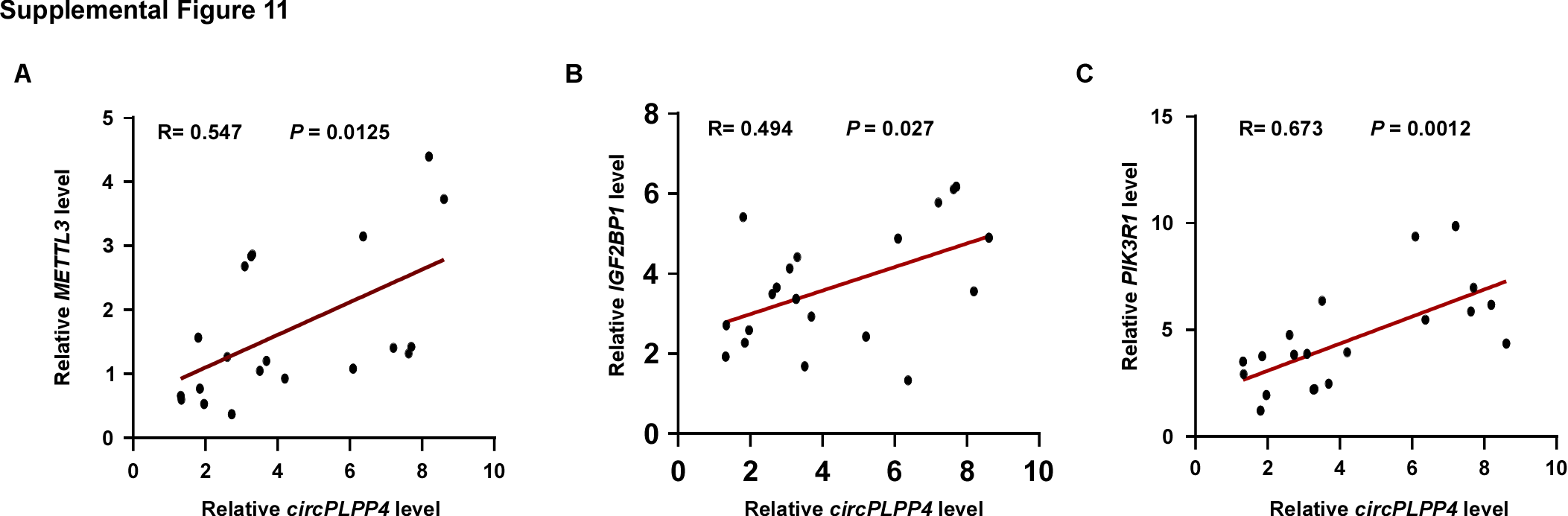

Supplement: Supplementary file 7 — Additional file 7: Supplemental Figure 2. (A) RT-qPCR analysis of circPLPP4 expression in the eight paired cisplatin-resistant ovarian cancer biopsies obtained before and after platinum-based chemotherapy. (B) RT-qPCR analysis of circPLPP4 expression in the eight paired cisplatin-sensitive ovarian cancer biopsies obtained before and after platinum-based chemotherapy.* P < 0.05, ** P < 0.01, *** P < 0.001, **** P < 0.0001, ns indicates no significance. Each error bar represents the mean ± SD of three independent experiments. [file 12943_2023_1917_MOESM7_ESM.tif]

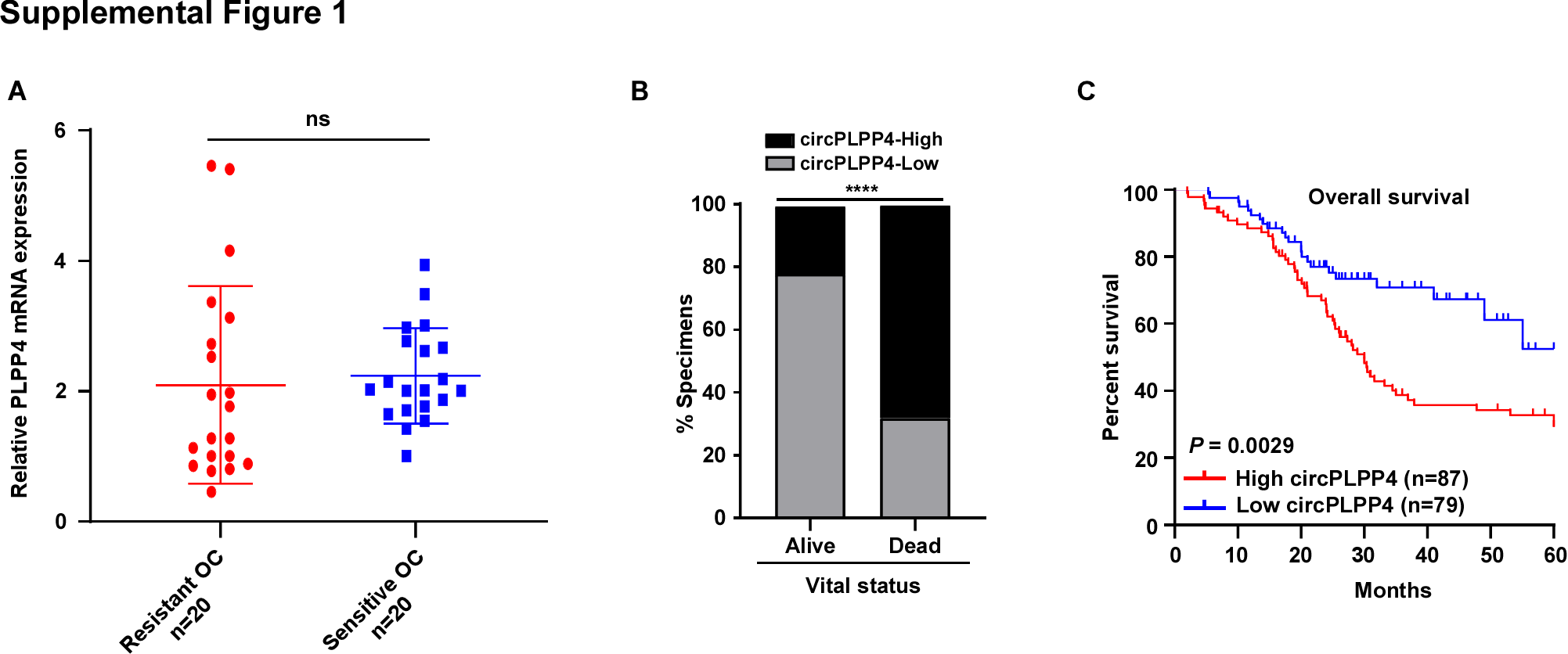

Supplement: Supplementary file 15 — Additional file 15: Supplemental Figure 10. (A) Representative images of circPLPP4 ISH analysis of OC tissue samples from OC patients. (B, C) Tumor weight and volume were examined in PDX-3 after 4-week treatment. (D) Tumor volumes were measured at the indicated time points in PDX-3. (E) Body weights of tumor-bearing mice (PDX-3) treated with CDDP combined with in vivo-optimized circPLPP4 inhibitor or control. (F, G) The expression of miR-136 and IGF2BP1 were analyzed using qRT-PCR in three PDX models. Statistical analyses were performed by unpaired Student’s t-test. * P< 0.05, ** P < 0.01, *** P < 0.001, **** P < 0.0001, ns indicates no significance. Each error bar represents the mean ± SD of three independent experiments. [file 12943_2023_1917_MOESM15_ESM.tif]

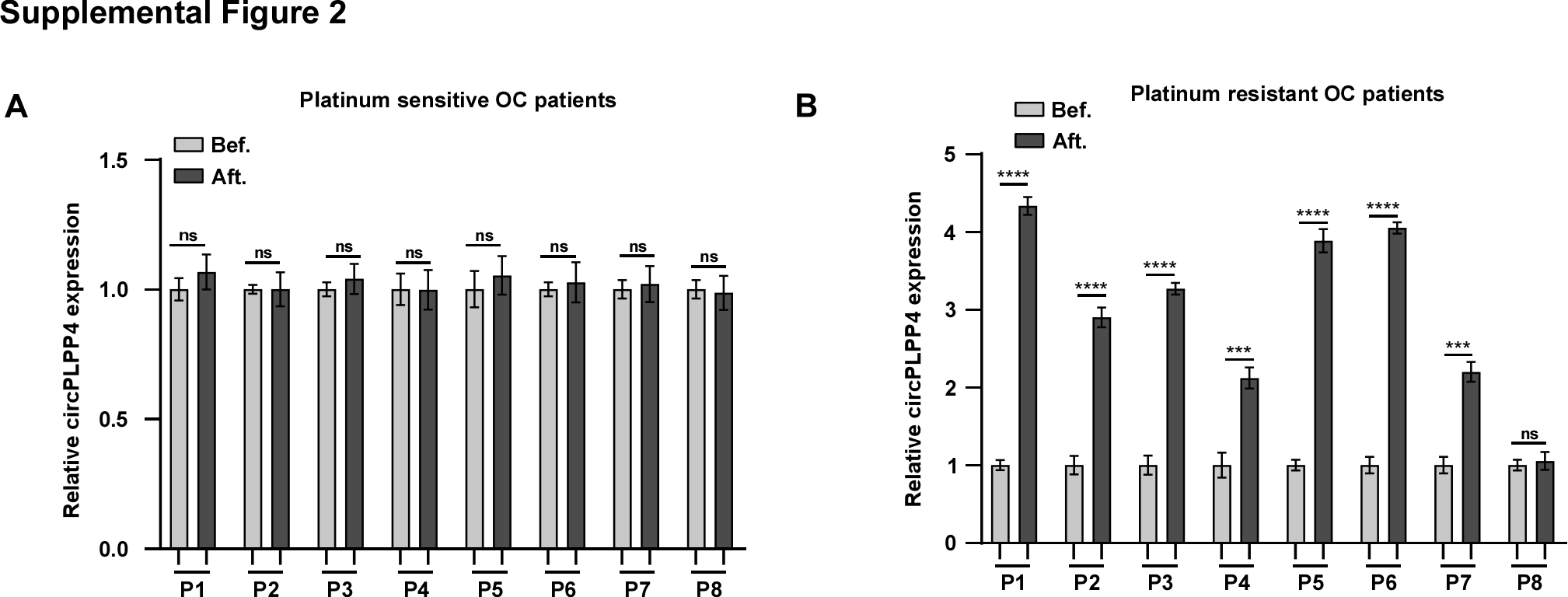

Supplement: Supplementary file 16 — Additional file 16: Supplemental Figure 11. Clinical relevance of m6A/ circPLPP4 /PIK3R1 axis in OC. (A-C) Correlation analysis showing the correlation between circPLPP4 and METTL3 (A), IGF2BP1 (B) or PIK3R1 (C) in OC specimens. Statistical analyses were performed by Spearman correlation coefficient. [file 12943_2023_1917_MOESM16_ESM.tif]

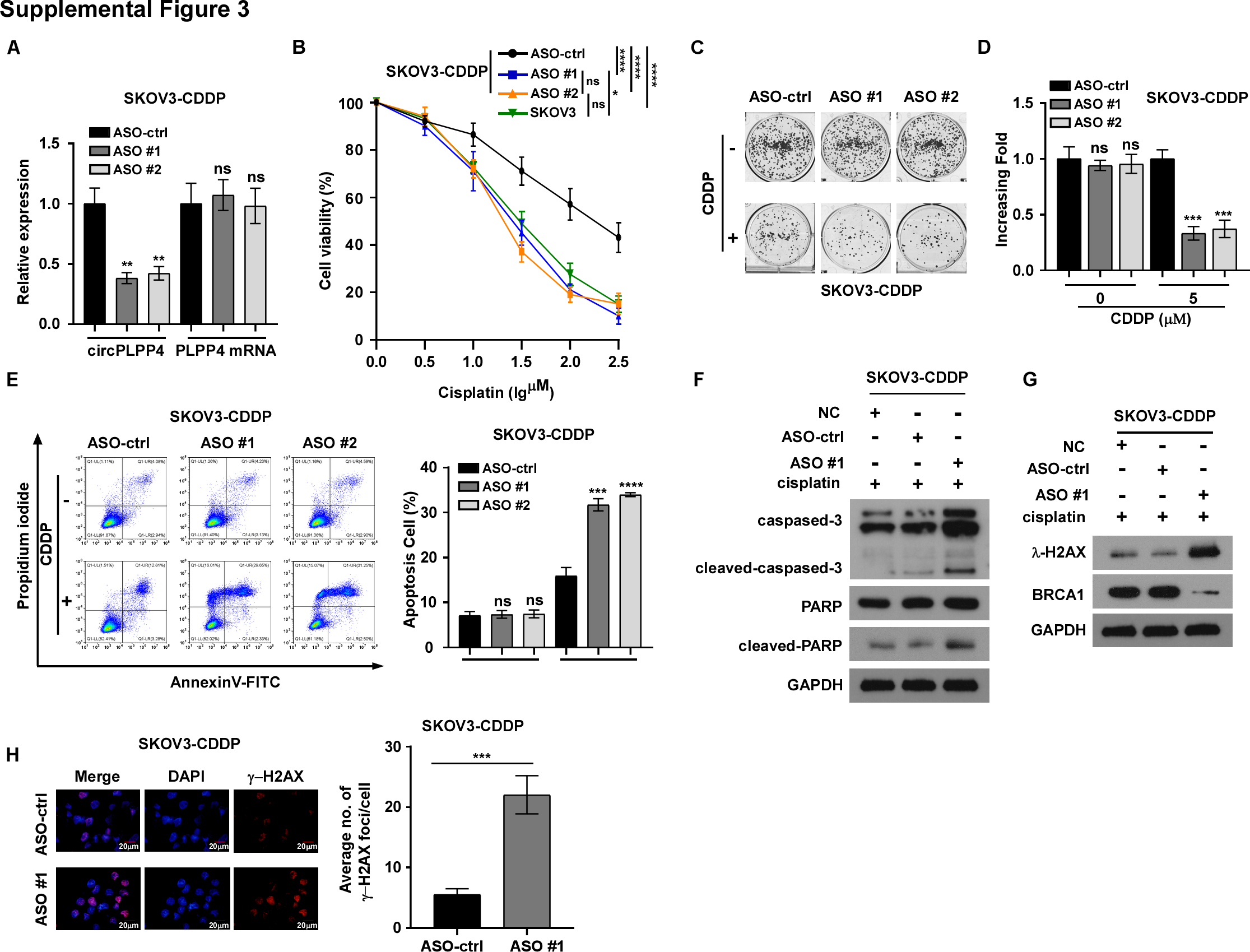

Supplement: Supplementary file 17 — Additional file 17. [file 12943_2023_1917_MOESM17_ESM.tif]

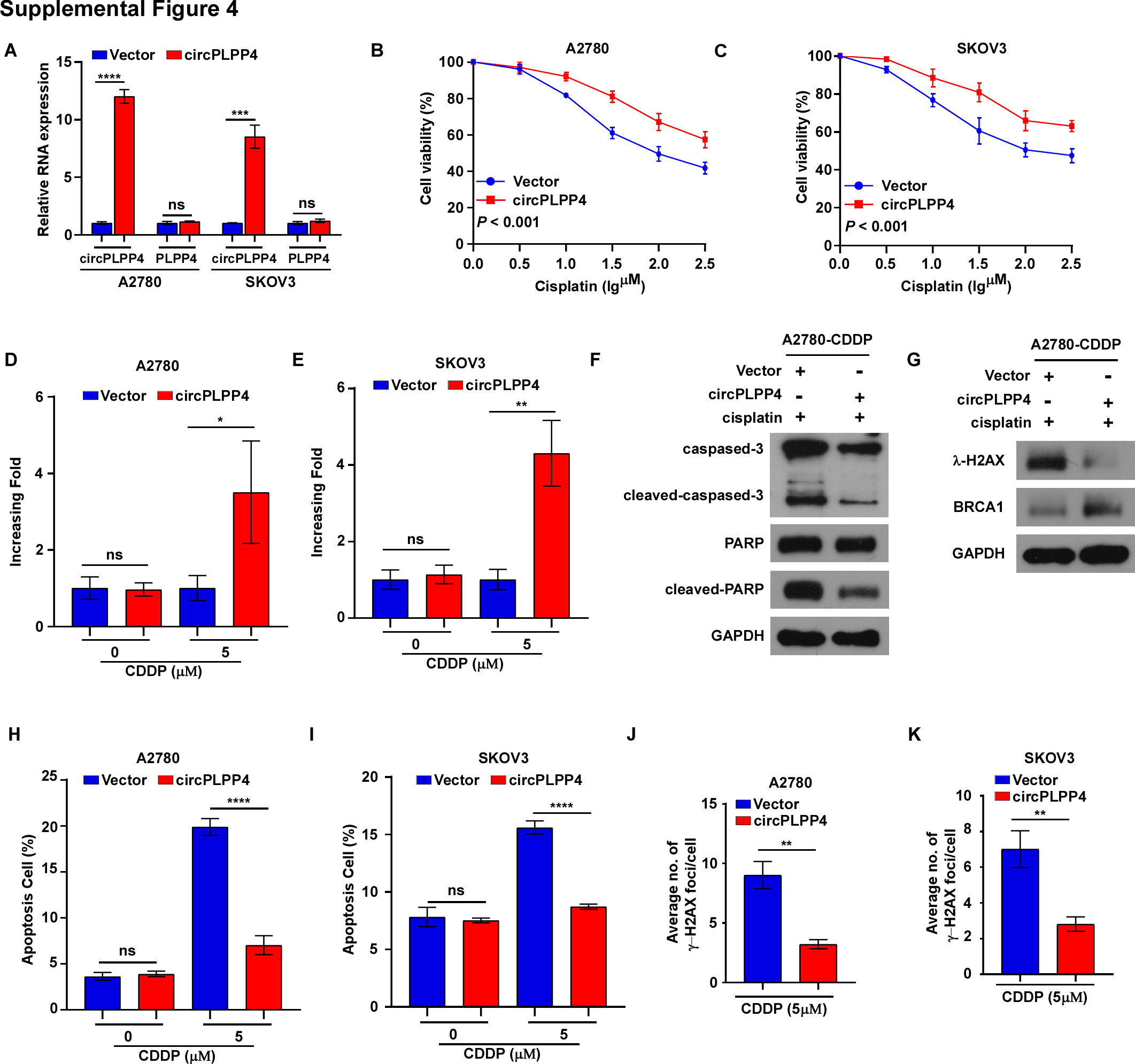

Supplement: Supplementary file 18 — Additional file 18. [file 12943_2023_1917_MOESM18_ESM.tif]
