# Supplementary material for: N6-methyladenosine-modified circPLPP4 sustains cisplatin resistance in ovarian cancer cells via PIK3R1 upregulation
Source: Mol Cancer. 2024 Jan 6;23:5. doi: 10.1186/s12943-023-01917-5 (PMC10770956; doi:10.1186/s12943-023-01917-5)
Supplement: Supplementary file 8 — Additional file 8: Supplemental Figure 3. circPLPP4 knockdown increased CDDP sensitivity of CDDP-resistant OC cells. (A) RT-qPCR analysis of circPLPP4 and PLPP4 expression in the ASO-ctrl or circPLPP4-ASO#1, circPLPP4-ASO#2 cells. (B) MTT cell viability assay of CDDP in the indicated OC cells. (C, D) Quantification of colony number of the indicated cells. (E) FACS analysis of Annexin V/PI staining of indicated cells treated with vehicle or CDDP (5μM) after 24 hours. (F) Western blotting analysis of level of caspased 3, cleaved-caspased 3, PARP and cleaved-PARP in the indicated cells. (G) Western blotting analysis of level of γ-H2AX and BRCA1 in the indicated cells. (H) Immunofluorescence staining and quantification of of γ-H2AX in the indicated cells treated with CDDP (5μM) after 24 hours. * P < 0.05, ** P < 0.01, *** P < 0.001, **** P < 0.0001, ns indicates no significance. Each error bar represents the mean ± SD of three independent experiments. [file 12943_2023_1917_MOESM8_ESM.docx]

**Supplemental Material**

**Methods**

**Immunoblotting and IHC analysis**

Immunoblotting was performed according to a previous study[1] using antibodies recognizing the following proteins: caspase 3 (#9662, Cell Signaling Technology (CST), Danvers, MA, USA), cleaved-caspase 3 (#9661, CST), PARP (#9532, CST), cleaved-PARP (#5625, CST), γ-H2AX (#80312, CST), BRCA1 (22362-1-AP, Proteintech, Wuhan, China), GAPDH (D16H11, CST), PIK3R1(60225-1-Ig, Proteintech, Wuhan, China), METTL3 (15073-1-AP, Proteintech), IGF2BP1 (22803-1-AP, Proteintech), and ALKBH5 (16837-1-AP, Proteintech). IHC was conducted on formalin-fixed, paraffin-embedded human tissue sections according to a previously published method [1]. IHC analysis was performed to determine altered protein levels in paraffin-embedded normal ovarian tissues and ovarian cancer tissues using anti‑PIK3R1(60225-1-Ig, Proteintech), anti-γ-H2AX (#80312, CST), anti‑cleaved‑caspase 3 (#9661, CST), anti- BRCA1 (22362-1-AP, Proteintech), anti‑METTL3 (15073-1-AP, Proteintech), and anti-IGF2BP1 (22803-1-AP, Proteintech) overnight at 4 ℃. The degree of immunostaining of the sections was examined and scored separately by two independent pathologists who were blinded to the histopathological features and patient data. The scores were determined by combining the proportion of positively-stained tumor cells and the intensity of staining, as reported previously [1] . Briefly, cell proportion scores were assigned as follows: 0, no positive cells; 1, <10% positive cells; 2, 10–35% positive cells; 3, 35–75% positive cells; and 4, >75% positive cells. Staining intensity was graded according to the following scale: 0, Negative (no staining); 1, Weak (light yellow) staining; 2, Moderate (yellow- brown) staining; and 3, Strong (brown) staining. The staining index (SI) was calculated as the product of the cell proportion and staining intensity scores of the positive cancer cells. Protein expression in OC tissue specimens was assessed according to SIs determined with the above method, with possible scores including 0, 1, 2, 3, 4, 6, 8, 9 and 12. Specimens with SI values ≥6 was defined as having high expression, while samples with SI values<6 was defined as having low expression.

**RNA extraction, and Quantitative Real-time Reverse Transcription PCR (qRT‑PCR)**

Total RNA was extracted from the indicated cells using the Trizol reagent (Life Technologies, Carlsbad, CA, USA) according to the manufacturer’s guidelines. cDNA was then synthesized using a Prime Script RT Master Mix Kit (RR036A, Takara), which served as the template for the quantitative real-time PCR (qPCR) step of the qRT-PCR protocol using GoTaq qPCR Master Mix (A6001, Promega, Madison, WI, USA) according to the manufacturer’s instructions. All data were analyzed and normalized to *GAPDH*. Primers and other reagents for mature miRNA assays were obtained from RiboBio (Guangzhou, China). Other primers for qPCR are listed in Table S4.

**Chemical reagents**

Actinomycin D (Act D, S8964) was purchased from Selleck Chemicals (Houston, TX, USA). RNase R (Cat#RNR07250; UW-Madison, USA) was purchased from Epicentre® (an Illumina company).

**RNase R treatment**

Two micrograms of total RNA were incubated for 30 min at 37℃ with or without 5U/μg RNase R (Epicentre Technlogies, USA). Subsequently, RNA was incubated at 70 °C for 10 min to inactivate RNase R and then reverse-transcribed for RT-PCR detection. The expression of *circPLPP4* and its linear counterpart mRNA *PLPP4* (encoding phospholipid phosphatase 4) were analyzed using qRT-PCR.

**Actinomycin D assay**

OC cells were transferred into six-well plates at 9 ×10^5^ cells per well and treated with actinomycin D (5 μg/ml); cells were collected at the indicated times. The expression of *circPLPP4* and its linear counterpart mRNA *PLPP4* (encoding phospholipid phosphatase 4) were analyzed using qRT-PCR.

**Colony formation assays**

Indicated OC Cells were seeded in 6-well plates (1×10^3^ cells per plate) and cultured for two weeks. The colonies were stained with 1% crystal violet for 30 minutes after fixation with 4% formaldehyde for 30 min. The number of colonies (defined as cell clusters consisting of at least 50 cells) was quantified using Analysis software (Olympus Biosystems, Tokyo, Japan).

**RNA fluorescence *in situ* hybridization**

Cy3-labelled *circPLPP4* probes were designed and synthesized by RiboBio. A fluorescence *in situ* hybridization (FISH) kit (RiboBio) was used to detect the probe signals in OC cells according to the manufacturer’s protocols. Nuclei were stained with 4,6-diamidino-2-phenylindole (DAPI). Images were acquired under a confocal microscope (Olympus FV1000).

**Patient-derived xenograft (PDX) models and the Tumor model**

To establish PDX models, fresh OC tumor samples from patients were inoculated immediately and subcutaneously into both flanks of NOD/ShiLtJGpt-Prkdc ^em26^Il2rg ^em26^/Gpt mice (GemPharmatech Co., Ltd., GuangDong, China). When the successfully established PDX tumors (P1) reached ~500 mm^3^, the tumors were transplanted into other mice (P2). Eventually, the mice bearing P3 grafts were used to evaluate the therapeutic effects of *circPLPP4* inhibitor (antisense oligonucleotides (ASOs) targeting *circPLPP4*). Three weeks after transplantation, tail vein injection of scrambled or *in vivo*-optimized *circPLPP4* ASO (Three dose of ASOs as follows: the ASO-H group,10 nmol per injection; the ASO-L1 group, 2 nmol per injection; the ASO-L2 group, 5 nmol per injection) was performed twice a week. The tumor volume and weight were measured after the mice were anesthetized at the end of the study. All tissues from the cell-based xenografts or PDXs underwent further pathological analysis. The subcutaneous tumor size was measured and recorded every 2 days using Vernier calipers as follows: tumor volume (mm^3^) = (L×W^2^)/2, where L is the long axis and W the short axis.

For the intraperitoneal tumor model, the indicated luciferase expressing cells (1 ×10^6^) were injected intraperitoneally into female nude mice. When the luminescence signal reached 2 × 10^7^ p/sec/cm^2^/sr, mice were treated intraperitoneally with vehicle (control), CDDP (5 mg/kg), or a combination of CDDP (5 mg/kg) three times every week (as per cycle) and the *circPLPP4* inhibitor (10 nmol per injection) twice a week. Mice were sacrificed when moribund, as determined by an observer blinded to the treatment, and tumors were excised and paraffin-embedded. For the subcutaneous cell derived xenograft (CDX) model, subcutaneous injections of 5 × 10^6^ A2780 cells/100μL in phosphate-buffered saline (PBS) and Matrigel (1:1) were performed into the axilla of 4–6 week-old female NOD/ShiLtJGpt-Prkdc ^em26^Il2rg ^em26^/Gpt mice (GemPharmatech Co.,Ltd, GuangDong, China). When the xenografts reached approximately 100 mm^3^, all the mice were treated with CDDP (5 mg/kg/ mice; three times per week). Twelve weeks later, the most resistant xenografts were isolated and mechanically disaggregated into approximately 1 mm^3^ tissue blocks to sub-transplant into the axilla of 4–6 week-old female NOD/ShiLtJGpt-Prkdc ^em26^Il2rg ^em26^/Gpt mice for the second CDX generation. When the xenografts reached approximately 100 mm^3^, the mice were randomized into two groups (five mice in each group): 1) negative control and 2) ASOs-targetting *circPLPP4*. All the mice were treated with CDDP (5 mg/kg/mouse; three times per week) and each tumor was injected via tail vein with ASOs-targeting *circPLPP4* or its negative control twice a week. Mice were euthanized when the study was finished and tumors were isolated for further study. All the animal procedures were approved by the Sun Yat-sen University Animal Care Committee (L102012021100F).

**Plasmids, Lentiviral infection, and transfection**

Small interfering RNAs (siRNAs) targeting *METTL3* (targeting sequences: #1: GCACTTGGATCTACGGAAT, #2: CGACTACAGTAGCTGCCTT) and *IGF2BP1* (targeting sequences: #1: GCTGGCTCAGTATGGTACAGT, #2: GGCTCAGTATGGTACAGTAGA) were synthesized by RiboBio. Lipofectamine 3000 (Thermo Fisher, Waltham, MA, USA) was used for transfection following the manufacturer’s instructions. Stable cell lines expressing *circPLPP4* were generated via Lentiviral infection and selected for 10 days using 0.5 µg/ml puromycin at 48 h after infection.

**Immunofluorescence (IF) staining**

OC cells were seeded on confocal dishes (Corning Inc., Corning, NY, USA), fixed with 4% paraformaldehyde solution, and permeabilized with or without 0.1% Triton X-100 on ice. After washing the cells with PBS three times, the cells were blocked with 5% bovine serum albumin (BSA) at 37°C for 30 minutes, and then incubated with primary antibodies overnight at 4 °C. After washing with PBS, the cells were incubated with the appropriate secondary antibody conjugated with a fluorescent dye for 1 h and then stained with antifade Mountant with DAPI (Thermo Fisher Scientific) for nuclear staining. Images were obtained via confocal microscopy (Olympus FV1000).

***In Situ* Hybridization (ISH)**

A B-D circRNA *in situ* hybridization Detection Kit (Exon Biotechnology, Guangzhou, China) was used for ISH staining, following the manufacturer’s guidelines. A biotin-labeled *circPLPP4* probe (sequence: 5’‑TGTGAGGAAAGAAATTGCAGGAGGAATGG-3’; Exon Biotechnology) was used to examine *circPLPP4* expression by ISH staining in paraffin-embedded and frozen OC tissues. The slides were stained with 3,3'‑diaminobenzidine (DAB) Enhanced Liquid Substrate System (Sigma, St. Louis, MO, USA). As previous published method[2, 3], the criteria for ISH staining as follows: the ISH scores of OC specimens were determined by two experienced pathologists. Tumor cell proportion scores were assigned as follows: 0, no positive cells; 1, <10% positive cells; 2, 10–35% positive cells; 3, 35–75% positive cells; and 4, >75% positive cells. The staining intensity was graded according to the following scale: 0, Negative staining (no staining); 1, Weak staining (light yellow); 2, Moderate staining (yellow- brown); and 3, Strong staining (brown). The staining index (SI) was calculated as the product of the cell proportion and staining intensity scores of the positive tumor cells. CircPLPP4 expression in OC tissue specimens was assessed according to SIs determined with the above method, with possible scores including 0, 1, 2, 3, 4, 6, 8, 9 and 12. Samples with SI values ≥6 was defined as having high expression, while samples with SI values<6 was defined as having low expression.

**Methylated RNA Immunoprecipitation (MeRIP) assays**

RNA immunoprecipitation was conducted using Protein A/G Agarose Beads (Santa Cruz Biotechnology, Santa Cruz, CA, USA) following the manufacturer’s instructions. Briefly, Protein A/G Agarose Beads coated with 5 mg of normal antibodies against rabbit immunoglobulin G (Thermo Scientific) or m^6^A (Abcam, Cambridge, MA, USA) were incubated with pre-frozen cell lysates or nuclear extracts overnight at 4 ℃. Associated RNA-protein complexes were collected and washed six times and then subjected to RNA extraction using TRIzol. The relative interaction between the protein and RNA was determined by qRT-PCR and normalized to the input.

**Dual-Luciferase reporter assay**

According to a previous published method[4, 5], the dual-luciferase reporter system (Promega) was employed for gene detection. The circPLPP4 and PIK3R1 3’UTR wild-type and mutant vectors were constructed using psiCheck and pmirGLO, respectively. CircPLPP4 or PIK3R1 3’UTR wild-type or mutant vectors and miR-136 mimic were co-transfected into A2780-CDDP and SKOV3-CDDP cells using Lipofectamine^TM^3000 reagent (Invitrogen, USA) according to the manufacturer’s protocol. Then the above transfected cells were seeded into 96-well plates. PLB (Passive Lysis buffer) cell lysis solution was added to each well after 48 h culture. Cells were fully lysed and the supernatant transferred into new tubes. PLB sample (20 μL) and LAR (100 μL) were added to each well of the 96-well plate, and Relative Light Units measured. After the addition of 100 μL Stop&Glo® Reagent into the same well, RLU values were re-analyzed.

**RNA pulldown assay**

Biotin-labeled *circPLPP4* and oligonucleotide probes were purchased from Exon Biotechnology. The probes were incubated with magnetic beads for 2 h at 25 °C. The indicated OC cell lysates were incubated overnight with the magnetic bead mixture at 4 °C. After purification, the enriched *circPLPP4* and miRNAs were quantified using qRT‑PCR. The *circPLPP4* pull down Probe was Biotin 5’‑TGTGAGGAAAGAAATTGCAGGAGGAATGG-3’.

**Annexin V Assay**

To quantify apoptotic cells, an Annexin V/propidium iodide (PI) Apoptosis Detection Kit (BD biosciences, San Jose, CA, USA) was used, following the manufacturer’s guidelines. Briefly, indicated OC cells were washed twice with PBS and suspended in the binding solution, to which was added 5 μl of an Annexin-V antibody and 5 μl of PI (for 1 × 10^6^ cells) in binding buffer and incubated for 15 min in the dark. Apoptotic cells were examined using flow cytometry[1].

**3-(4,5-dimethylthiazol-2-yl)-2,5-diphenyltetrazolium bromide (MTT) cell viability assay**

The MTT assay was conducted according to a previous study [1]. Briefly, the indicated OC cells (2 × 10^3^) were treated for 24 h with CDDP (5 μM), stained with sterile MTT dye (0.5 mg/ml, Sigma) for 4 h at 37 °C, after which the culture medium was removed and dimethyl sulphoxide (DMSO) added (Sigma-Aldrich). The absorbance was measured at 570 nm, with 655 nm as the reference wavelength. Dose‑response curves were plotted on a semilog scale as the percentage of the control cell number, which was obtained from the sample with no drug treatment. The IC50 values were calculated using GraphPad Prism® 8 (Version 8.01, GraphPad Inc., La Jolla, CA, USA).

**Terminal deoxynucleotidyl transferase-mediated dUTP Nick end labeling (TUNEL) staining**

A One Step TUNEL Apoptosis Assay Kit (Beyotime C1090, Shanghai, China) was used to detect the TUNEL+ cells according to the manufacturer’s guidelines. Briefly, the indicated OC cells were fixed and permeabilized after CDDP treatment. Then, the apoptotic cells were observed and photographed under a fluorescence microscope. Finally, TUNEL+ cells and total cells were analyzed using ImageJ software (NIH, Bethesda, MD, USA).

**ASO‑mediated knockdown and ASO *in vivo* treatment**

The ASOs targeting *circPLPP4* were obtained from RiboBio (Guangzhou, China). Transfections with ASOs (50 nM) were performed following the manufacturer’s guidelines. After transfection, we collected RNA and protein for further experiments. For the *in vivo* experiment, 10 nmol ASOs were delivered into the mice through tail vein injection twice a week.

**Statistical analysis**

Statistical analysis was conducted using the Student’s two-tailed t-test and One-way analysis of variance (ANOVA). Bivariate correlations between study variables were calculated using Spearman’s rank correlation coefficients. Survival curves were plotted using the Kaplan–Meier method and compared using the log-rank test. The significance of survival-related variables was analyzed using univariate and multivariate Cox regression analyses. P-values < 0.05 was considered statistically significant. Data are presented as the mean ± standard deviation (SD). Statistical analysis was performed using GraphPad 19.0 statistical software. In the figures, the P-values are shown as *P < 0.05, **P < 0.01, ***P < 0.001, and ns: not significant.

**References**

[1] H. Li, W. Zhang, C. Niu, C. Lin, X. Wu, Y. Jian, Y. Li, L. Ye, Y. Dai, Y. Ouyang, J. Chen, J. Qiu, L. Song, Y. Zhang, Nuclear orphan receptor NR2F6 confers cisplatin resistance in epithelial ovarian cancer cells by activating the Notch3 signaling pathway, Int J Cancer, (2019).

[2] H. Li, F. Luo, X. Jiang, W. Zhang, T. Xiang, Q. Pan, L. Cai, J. Zhao, D. Weng, Y. Li, Y. Dai, F. Sun, C. Yang, Y. Huang, J. Yang, Y. Tang, Y. Han, M. He, Y. Zhang, L. Song, J.C. Xia, CircITGB6 promotes ovarian cancer cisplatin resistance by resetting tumor-associated macrophage polarization toward the M2 phenotype, J Immunother Cancer, 10 (2022).

[3] J. Chen, A. Liu, Z. Wang, B. Wang, X. Chai, W. Lu, T. Cao, R. Li, M. Wu, Z. Lu, W. Pang, L. Xiao, X. Chen, Y. Zheng, Q. Chen, J. Zeng, J. Li, X. Zhang, D. Ren, Y. Huang, LINC00173.v1 promotes angiogenesis and progression of lung squamous cell carcinoma by sponging miR-511-5p to regulate VEGFA expression, Mol Cancer, 19 (2020) 98.

[4] L. Chen, A. Nan, N. Zhang, Y. Jia, X. Li, Y. Ling, J. Dai, S. Zhang, Q. Yang, Y. Yi, Y. Jiang, Circular RNA 100146 functions as an oncogene through direct binding to miR-361-3p and miR-615-5p in non-small cell lung cancer, Mol Cancer, 18 (2019) 13.

[5] C. Hu, R. Xia, X. Zhang, T. Li, Y. Ye, G. Li, R. He, Z. Li, Q. Lin, S. Zheng, R. Chen, circFARP1 enables cancer-associated fibroblasts to promote gemcitabine resistance in pancreatic cancer via the LIF/STAT3 axis, Mol Cancer, 21 (2022) 24.
