# Supplementary material for: N6-methyladenosine-modified circPLPP4 sustains cisplatin resistance in ovarian cancer cells via PIK3R1 upregulation
Source: Mol Cancer. 2024 Jan 6;23:5. doi: 10.1186/s12943-023-01917-5 (PMC10770956; doi:10.1186/s12943-023-01917-5)
Supplement: Supplementary file 10 — Additional file 10: Supplemental Figure 5. (A-C) The expression of SLAMF7, UGT1A4 and PRDM8 were analyzed using qRT-PCR in A2780 CDDP, SKOV3 CDDP, A2780 and SKOV3 cells. Error bars represent the mean ± SD of three experiments. Statistical analyses were performed by unpaired Student’s t-test. * P < 0.05, ** P < 0.01, *** P < 0.001, **** P < 0.0001, ns indicates no significance. Each error bar represents the mean ± SD of three independent experiments. [file 12943_2023_1917_MOESM10_ESM.docx]

**Table 1. Clinicopathological characteristics and expression of circPLPP4 in ovarian cancer.**

| **Characteristic** | **Cases, n (%)** |
| --- | --- |
| **Age (years)** |  |
| ≤52 | 92(55.4) |
| >52 | 74(44.6) |
| **FIGO stage** |  |
| I | 8(6.9) |
| II | 30(18.1) |
| III | 115(69.3) |
| IV | 13(7.8) |
| **Histological type** |  |
| Serous adenocarcinoma | 91(54.8) |
| Mucoid adenocarcinoma | 67(40.4) |
| Endometrial adenocarcinoma | 7(4.2) |
| Clear cell carcinoma | 1(0.6) |
| **Neo-adjuvant chemotherapies** |  |
| No | 113 (68.1) |
| Yes | 53 (31.9) |
| **Intraperitoneal metastasis** |  |
| No | 66(39.8) |
| Yes | 100(60.2) |
| **Intestinal metastasis** |  |
| No  Yes | 54(32.5)  112(67.5) |
| **CircPLPP4 Expression** |  |
| Low  High | 73(44.0)  93(56.0) |
| **Vital status at last follow-up** |  |
| Alive | 75(45.2) |
| Dead | 91(54.8) |
| **Tumor recurrence** |  |
| No | 57(34.3) |
| Yes | 109(65.7) |
|  |  |
| **Drug resistance** |  |
| No  Yes  **Ascites with tumor cells**  No  Yes  **Lymph node metastasis**  No  Yes | 94(56.6)  72(43.4)  47(28.3)  119(71.7)  54(32.5)  112(67.5) |
