# Supplementary material for: N6-methyladenosine-modified circPLPP4 sustains cisplatin resistance in ovarian cancer cells via PIK3R1 upregulation
Source: Mol Cancer. 2024 Jan 6;23:5. doi: 10.1186/s12943-023-01917-5 (PMC10770956; doi:10.1186/s12943-023-01917-5)
Supplement: Supplementary file 11 — Additional file 11: Supplemental Figure 6. circPLPP4 enhanced PIK3R1 expression by sponging miR-136 in OC cells. (A) The expression levels of PIK3R1 were examined using qRT-PCR in the indicated cells. (B) The expression levels of PIK3R1 were analyzed using qRT-PCR. SKOV3 cells were transfected with the indicated vectors and miR-136 mimics. (C) The expression levels of PIK3R1 were analyzed using qRT-PCR in the indicated cells. (D, E). The proteins levels of PIK3R1, apoptosis markers, γH2AX, BRCA1 were detected using western blotting in A2780 and SKOV3 cells transfected with the indicated vectors and miR-136 mimics after CDDP treatment (5μM). (F) The IC50 was detected by the MTT assay. SKOV3 CDDP cells were transfected with miR-136 mimic alone or co-transfected with the indicated vectors upon CDDP exposure (5 μM) for 48 h. (G) The apoptosis rates of SKOV3CDDP cells transfected with miR-136 mimic alone or co-transfected with the indicated vectors upon CDDP treatment (5 μM) for 48 h. The results are presented as the mean ± SEM. * P < 0.05, ** P < 0.01, *** P < 0.001, **** P < 0.0001, ns indicates no significance. Each error bar represents the mean ± SD of three independent experiments. [file 12943_2023_1917_MOESM11_ESM.docx]

**Table 2. Correlation between circPLPP4 expression and the clinicopathological features of ovarian cancer.**

| **Characteristic** | | **Total** | **CircPLPP4 Expression** | | **Chi-square**  **Test**  ***p*-value** | **Fisher’s Exact Test**  ***p*-value** |
| --- | --- | --- | --- | --- | --- | --- |
|  |  |  | **Low, n (%)** | **High, n (%)** |  |  |
| **Age (years)** | ≤52 | 92 | 48(52.2) | 44(47.8) | 0.187 | 0.213 |
|  | >52 | 74 | 31(41.9) | 43(58.1) |  |  |
| **Histological type** | Serous adenocarcinoma | 91 | 45(49.5) | 46(50.5) | 0.515 | - |
|  | Mucoid adenocarcinoma  Endometrial adenocarcinoma  Clear cell carcinoma | 67  7  1 | 31(46.3)  2(28.6)  1(100.0) | 36(53.7)  5(71.4)  0(0.00) |  |  |
| **FIGO stage** | I | 8 | 3(37.5) | 5(62.5) | 0.421 | - |
|  | II | 30 | 17(56.7) | 13(43.3) |  |  |
|  | III | 115 | 51(44.3) | 64(55.7) |  |  |
|  | IV | 13 | 8(61.5) | 5(38.5) |  |  |
|  |  |  |  |  |  |  |
| **Ascites with tumor cells** | No  Yes | 47  119 | 21(44.7)  58(43.7) | 26(55.3)  61(56.3) | 0.637 | 0.731 |
|  |  |  |  |  |  |  |
| **Intraperitoneal metastasis** | No | 66 | 44(66.7) | 22(33.3) | <0.001 | <0.001 |
|  | Yes | 100 | 35(35.0) | 65(65.0) |  |  |
|  |  |  |  |  |  |  |
| **Intestinal metastasis** | No  Yes | 54  112 | 27(50.0)  52(46.4) | 27(50.0)  60(53.6) | 0.666 | 0.741 |
|  |  |  |  |  |  |  |
| **Vital status at last follow-up** | Alive  Dead | 91  75 | 60(52.2)  19(25.3) | 31(47.8)  56(74.7) | <0.001 | <0.001 |
|  |  |  |  |  |  |  |
| **Tumor** | No | 79 | 41(51.9) | 38(48.1) | 0.290 | 0.351 |
| **recurrence** | Yes | 87 | 38(43.7) | 49(56.3) |  |  |
| **Lymph node metastasis** | No  Yes | 54  112 | 37(68.5)  42(37.5) | 17(31.5)  70(62.5) | <0.001 | <0.001 |
| **Drug resistance** | No  Yes | 80  86 | 50(62.5)  29(33.7) | 30(37.5)  57(66.3) | <0.001 | <0.001 |
