# Supplementary material for: N6-methyladenosine-modified circPLPP4 sustains cisplatin resistance in ovarian cancer cells via PIK3R1 upregulation
Source: Mol Cancer. 2024 Jan 6;23:5. doi: 10.1186/s12943-023-01917-5 (PMC10770956; doi:10.1186/s12943-023-01917-5)
Supplement: Supplementary file 12 — Additional file 12: Supplemental Figure 7. m6A modification contributes to the upregulation of circPLPP4 in OC. (A) qRT-PCR analysis of circPLPP4 expression in the indicated cells with or without treatment of 5-zaz-dC. (B) RT-qPCR analysis of circPLPP4 expression in the indicated cells with or without treatment of SAHA or NaB. (C) qRT-PCR analysis of mRNA expression in 20 OC tissues and 20 normal ovary tissues. (D) Western blotting of ALKBH5 expression in the indicated cells. GAPDH served as the loading control. (E). qRT-PCR analysis of circPLPP4 in the indicated cells. (F) RIP analysis showing the enrichment of circPLPP4 on several proteins in the indicated cells. (G) qRT-PCR analysis of circPLPP4 expression in the indicated cells. (H) Nuclear–cytoplasmic fractionation assays revealing circPLPP4 expression in cytoplasm and nucleus of control or METTL3-knockdown the indicated OC cells. U3 and GAPDH were used as positive controls in the nucleus and cytoplasm, respectively. * P < 0.05,** P < 0.01, *** P < 0.001, **** P < 0.0001, ns indicates no significance. Each error bar represents the mean ± SD of three independent experiments. [file 12943_2023_1917_MOESM12_ESM.docx]

|  | **Univariate Analysis** | | | | **Multivariate Analysis** | | |
| --- | --- | --- | --- | --- | --- | --- | --- |
| **Variable** | **Patients (n)** | ***p*** | **Regression Coefficient (SE)** | ***p*** | | **Relative Risk** | **95% Confidence Interval** |
| **CircPLPP4** |  |  |  |  | |  |  |
| Low expression | 73 | <0.001 | 1.200(0.266) | 0.002 | | 2.415 | 1.378-4.232 |
| High expression | 93 |  |  |  |  |  |  |
| **Intraperitoneal metastasis** |  |  |  |  | |  |  |
| No | 66 | <0.001 | 1.275(0.290) | 0.002 | | 2.538 | 1.393-4.618 |
| Yes | 100 |  |  |  |  |  |  |
| **Lymph node metastasis** |  |  |  |  |  |  |  |
| No | 54 | 0.008 | 0.735(0.276) | 0.904 | | 0.965 | 0.537-1.732 |
| Yes | 112 |  |  |  |  |  |  |
| **Drug resistance**  No | 94 | <0.001 | 1.030(0.249) | <0.001 | | 2.740 | 1.634-4.593 |
| Yes | 72 |  |  |  | |  |  |
| **Tumor recurrence** |  |  |  |  | |  |  |
| No | 57 | 0.002 | 0.775(0.245) | 0.019 | | 1.836 | 1.104-3.052 |
| Yes | 109 |  |  |  |  |  |  |
| **FIGO stage** |  |  |  |  | |  |  |
| I | 8 |  |  |  | |  |  |
|  |  |  |  |  | |  |  |
| II | 30 |  |  |  | |  |  |
|  |  | 0.010 | 0.487(0.189) | 0.031 | | 1.640 | 1.046-2.571 |
| III | 115 |  |  |  | |  |  |
|  |  |  |  |  | |  |  |
| IV | 13 |  |  |  | |  |  |
|  |  |  |  |  | |  |  |

**Table 3. Cox regression univariate and multivariate analyses of prognostic factors in ovarian cancer**
