# Supplementary material for: N6-methyladenosine-modified circPLPP4 sustains cisplatin resistance in ovarian cancer cells via PIK3R1 upregulation
Source: Mol Cancer. 2024 Jan 6;23:5. doi: 10.1186/s12943-023-01917-5 (PMC10770956; doi:10.1186/s12943-023-01917-5)
Supplement: Supplementary file 13 — Additional file 13: Supplemental Figure 8. (A) Western blotting of PIK3R1, γ-H2AX, caspase 3, cleaved caspase 3 and BRCA1 expression in the indicated A2780-CDDP tumor. GAPDH served as the loading control. [file 12943_2023_1917_MOESM13_ESM.docx]

**Table S4 qRT-PCR primers**

circPLPP4 divergent primer (forward: 5’--3’: GCATCCATTCCTCCTGCAA, reverse: 5’--3’: GCAGACTCCATTCAAAGCAAG.);

circPLPP4 convergent primer (forward: 5’--3’: GCCTTCTTAGCGGTGTCCTT, reverse: 5’--3’: AAAAGAAATCGGGGCGAGGT.);

METTL3 (forward: 5’--3’: TTGTCTCCAACCTTCCGTAGT, reverse: 5’--3’: CCAGATCAGAGAGGTGGTGTAG.);

METTL14 (forward: 5’--3’: GAGTGTGTTTACGAAAATGGGGT, reverse: 5’--3’: CCGTCTGTGCTACGCTTCA.);

WTAP (forward: 5’--3’: ACTGGCCTAAGAGAGTCTGAAG, reverse: 5’--3’: GTTGCTAGTCGCATTACAAGGA.);

ALKBH5 (forward: 5’--3’: CGGCGAAGGCTACACTTACG, reverse: 5’--3’: CCACCAGCTTTTGGATCACCA.);

FTO (forward: 5’--3’: GCTGCTTATTTCGGGACCTG, reverse: 5’--3’: AGCCTGGATTACCAATGAGGA.);

YTHDF1 (forward: 5’--3’: ACCTGTCCAGCTATTACCCG, reverse: 5’--3’: TGGTGAGGTATGGAATCGGAG.);

YTHDF2 (forward: 5’--3’: CCTTAGGTGGAGCCATGATTG, reverse: 5’--3’: TCTGTGCTACCCAACTTCAGT.);

YTHDF3 (forward: 5’--3’: GGTGTATTTAGTCAACCTGGGG, reverse: 5’--3’: AAGAGAACTAGGTGGATAGCCAT.);

IGF2BP1 (forward: 5’--3’: GCTCTTTGGGGACAGGAAGC, reverse: 5’--3’: GGAGCTCACCTCTTCATCCG.);

IGF2BP2 (forward: 5’--3’: AGCTAAGCGGGCATCAGTTTG, reverse: 5’--3’: CCGCAGCGGGAAATCAATCT.);

IGF2BP3 (forward: 5’--3’: TATATCGGAAACCTCAGCGAGA, reverse: 5’--3’: GGACCGAGTGCTCAACTTCT.);

YTHDC1 (forward: 5’--3’: TCAGGAGTTCGCCGAGATGTGT, reverse: 5’--3’: AGGATGGTGTGGAGGTTGTTCC.);

YTHDC2 (forward: 5’--3’: GAAAGCTCCTGAACCTCCACCA, reverse: 5’--3’:

GGTTCTACTGGCAAGTCAGCCA.);

PLPP4 (forward: 5’--3’: TGCCCTTGTACTGCGCCATGAT, reverse: 5’--3’:

GGAGGATAGTGCTGTCTGTAGC.);

PIK3R1(forward: 5’--3’: CGCCTCTTCTTATCAAGCTCGTG, reverse: 5’--3’:

GAAGCTGTCGTAATTCTGCCAGG.);

GAPDH (forward: 5’--3’: GTCTCCTCTGACTTCAACAGCG, reverse: 5’--3’:

ACCACCCTGTTGCTGTAGCCAA.)

U3 (forward: 5’--3’: TTCTCTGAGCGTGTAGAGCACCGA, reverse: 5’--3’:

GATCATCAATGGCTGACGGCAGTT.)
