# Supplementary material for: N6-methyladenosine-modified circPLPP4 sustains cisplatin resistance in ovarian cancer cells via PIK3R1 upregulation
Source: Mol Cancer. 2024 Jan 6;23:5. doi: 10.1186/s12943-023-01917-5 (PMC10770956; doi:10.1186/s12943-023-01917-5)
Supplement: Supplementary file 14 — Additional file 14: Supplemental Figure 9. (A) representative images of tumors with different treatment. (B, C)Tumor weight and tumor volume of A2780 CDX model treated with different dose of ASOs or PBS control. n = 5 for each group. Tumor volume (mm3)= (L × W2)/2, where L is the long axis and W the short axis. (D) representative images of tumors with different treatment. (E, F)Tumor weight and tumor volume of SKOV3 CDX model treated with different dose of ASOs or PBS control. n = 5 for each group. Tumor volume (mm3) = (L × W2)/2, where L is the long axis and W the short axis. * P < 0.05, ** P < 0.01, *** P < 0.001, **** P < 0.0001, ns indicates no significance. [file 12943_2023_1917_MOESM14_ESM.docx]

**Supplementary Table 5**

**siRNA, ASO & shRNA sequence**

Name Sequence (5’-3’)

si-IGF2BP2 GGAAAGGAGGGCTTGACCATA

si-METTL3 GCACTTGGATCTACGGAAT

si-IGF2BP1 CCGGGAAAGTAGAATTACAAGGAAA

ASO -circPLPP4#1: AGCATCCATTCCTCCTGCAA

ASO -circ PLPP4#2: CATCCATTCCTCCTGCAATT
