# Supplementary material for: N6-methyladenosine-modified circPLPP4 sustains cisplatin resistance in ovarian cancer cells via PIK3R1 upregulation
Source: Mol Cancer. 2024 Jan 6;23:5. doi: 10.1186/s12943-023-01917-5 (PMC10770956; doi:10.1186/s12943-023-01917-5)

# Figure 2M

Cleaved caspase 3

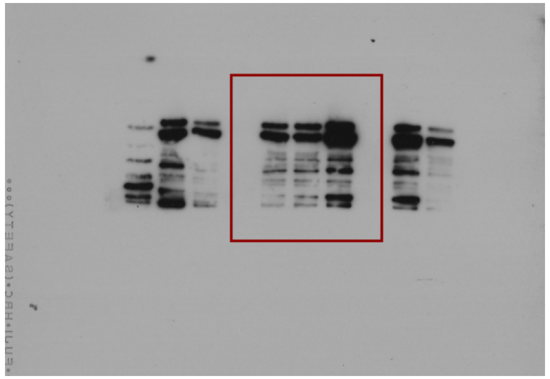

PARP

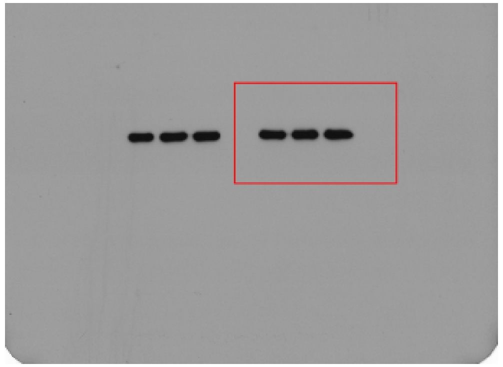

Cleaved PARP

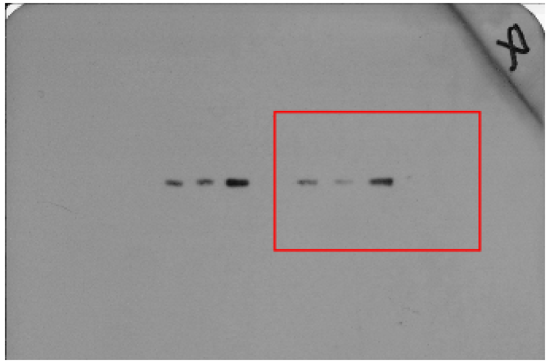

GAPDH

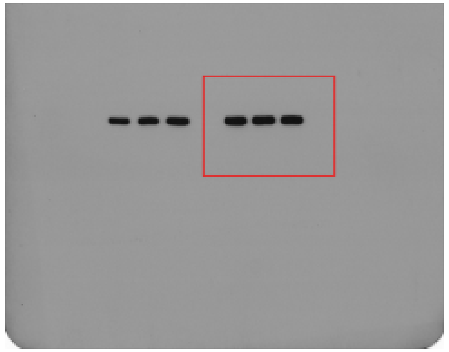

# Figure 2N

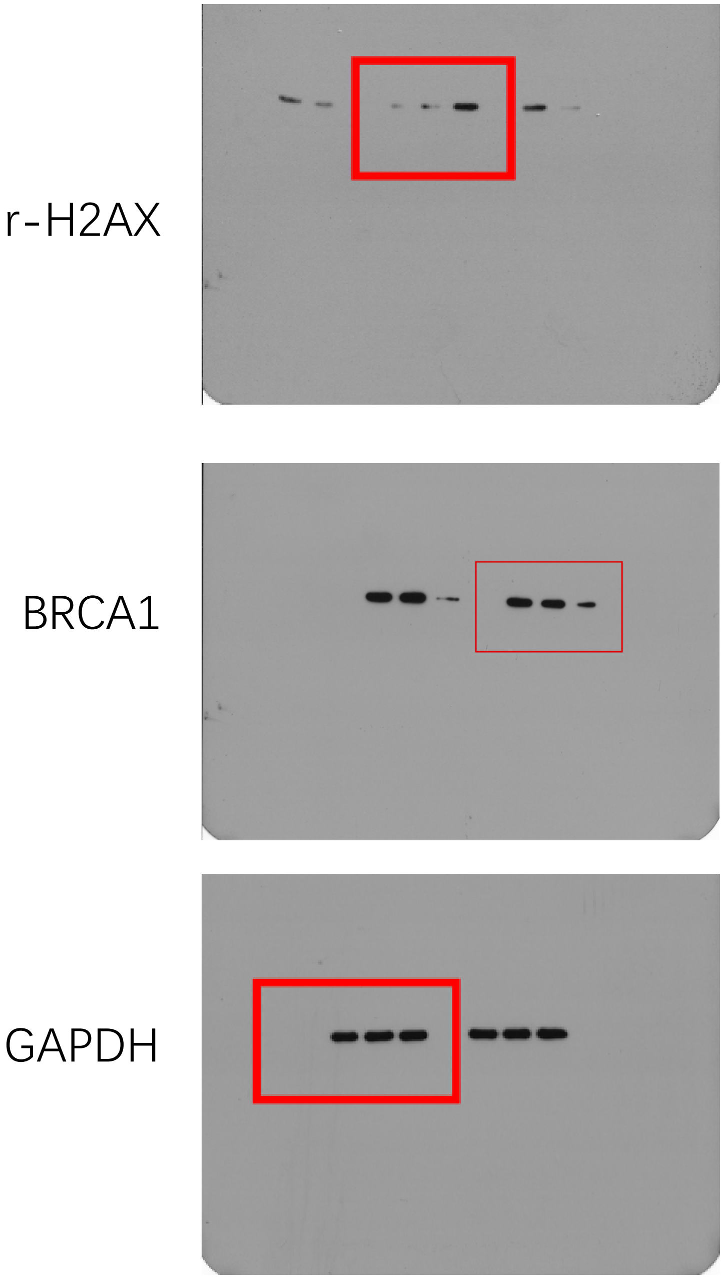

**Figure 4D**

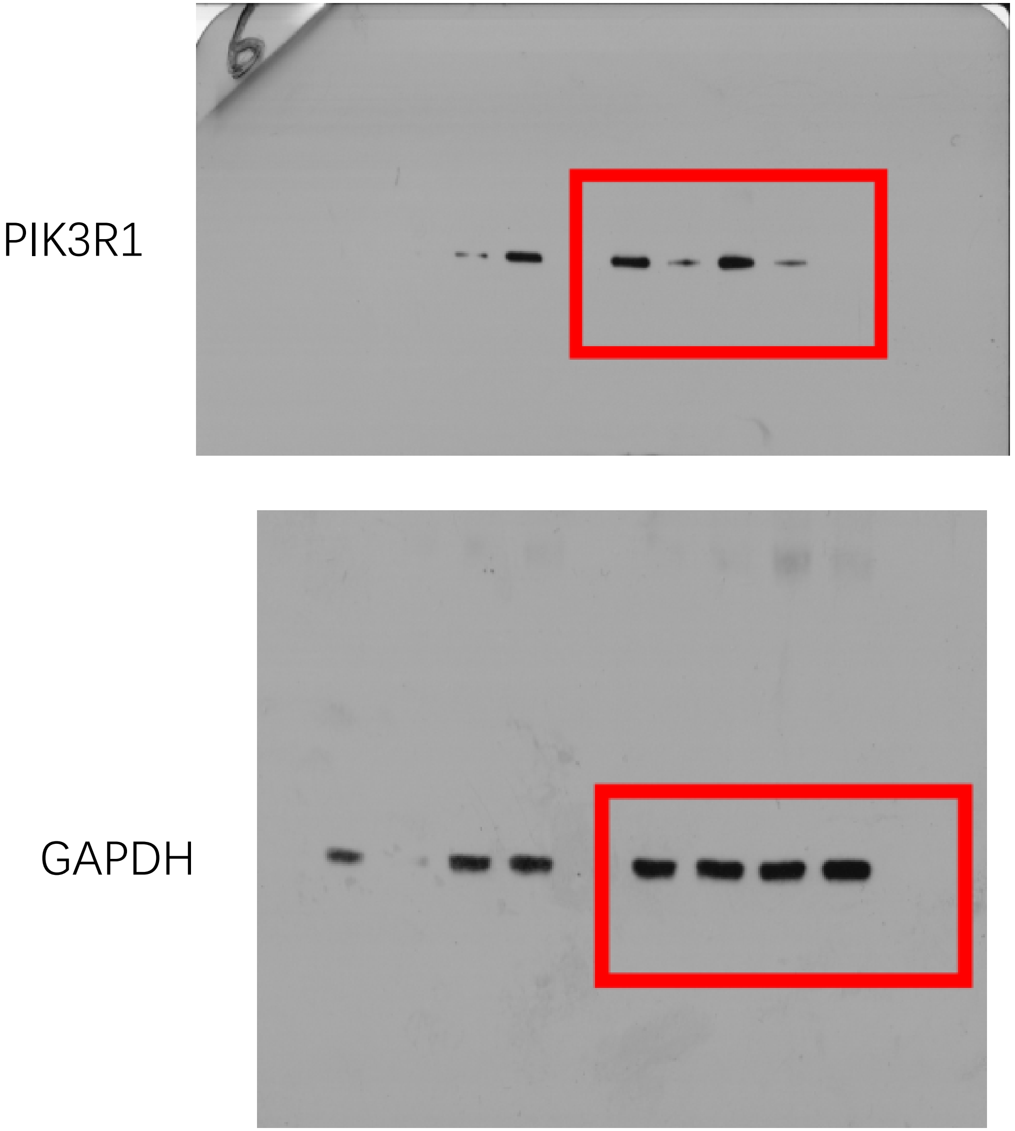

**Figure 4J** **A2780-CDDP**

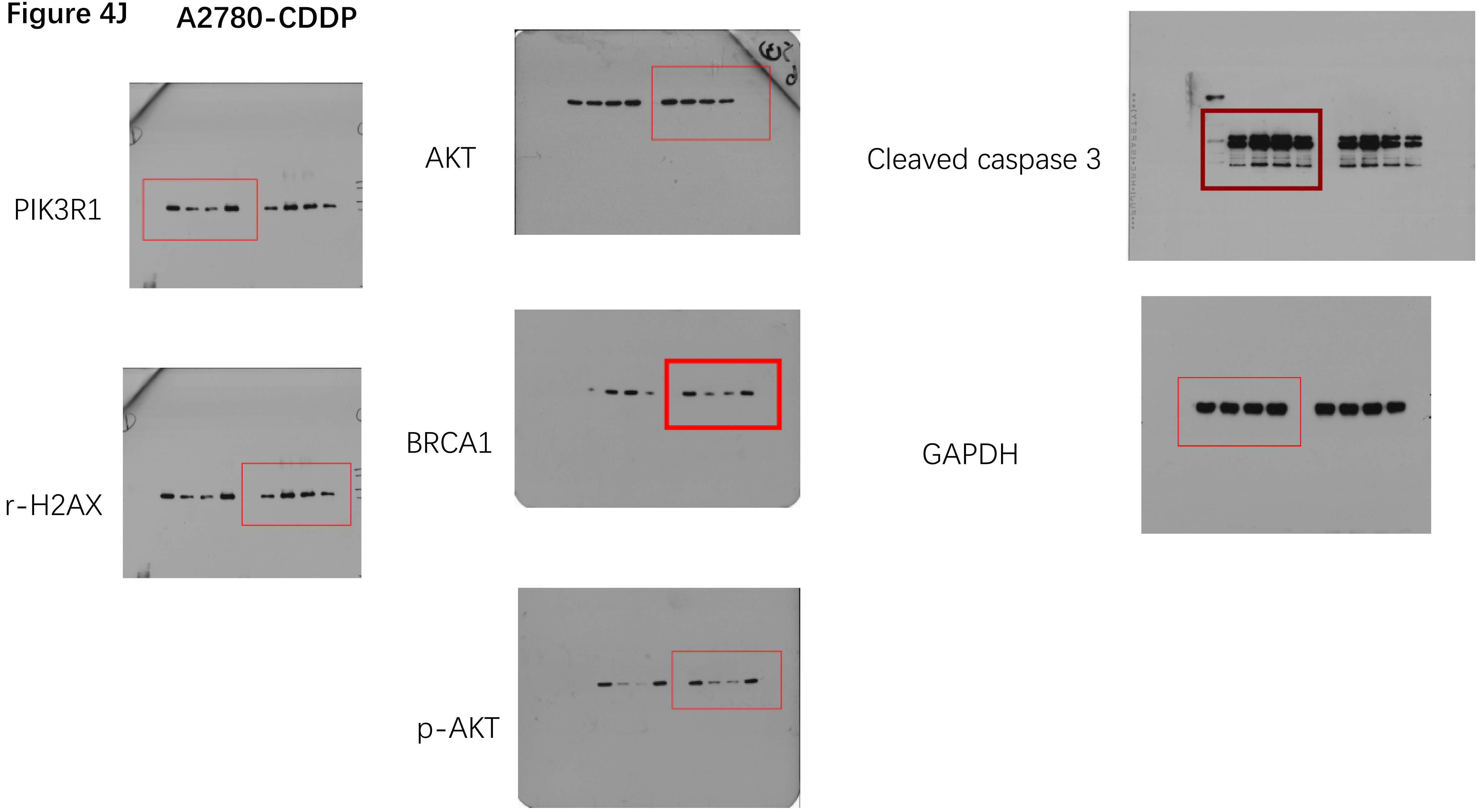

# Figure 4J SKOV3-CDDP

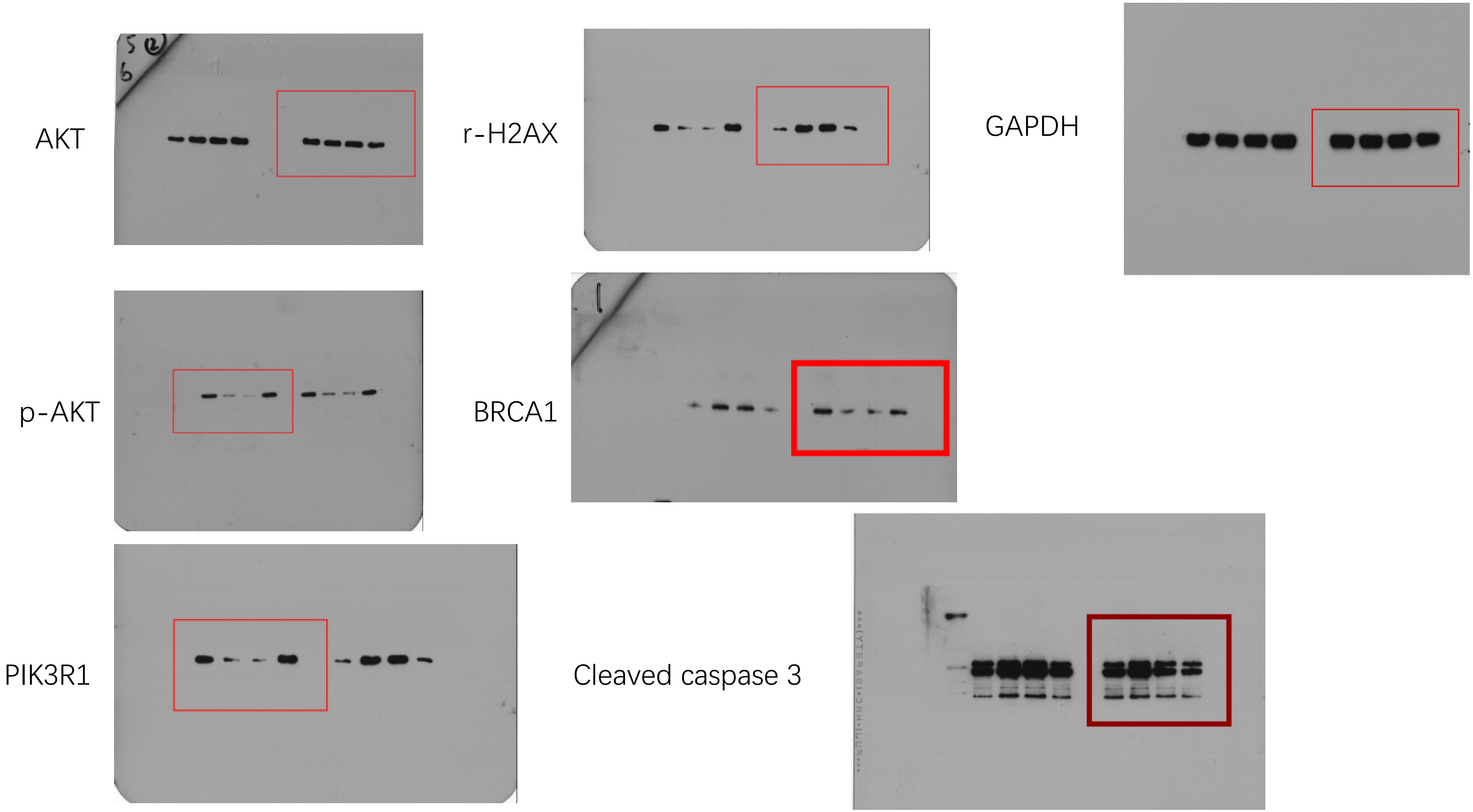

**Figure 4M A2780-CDDP**

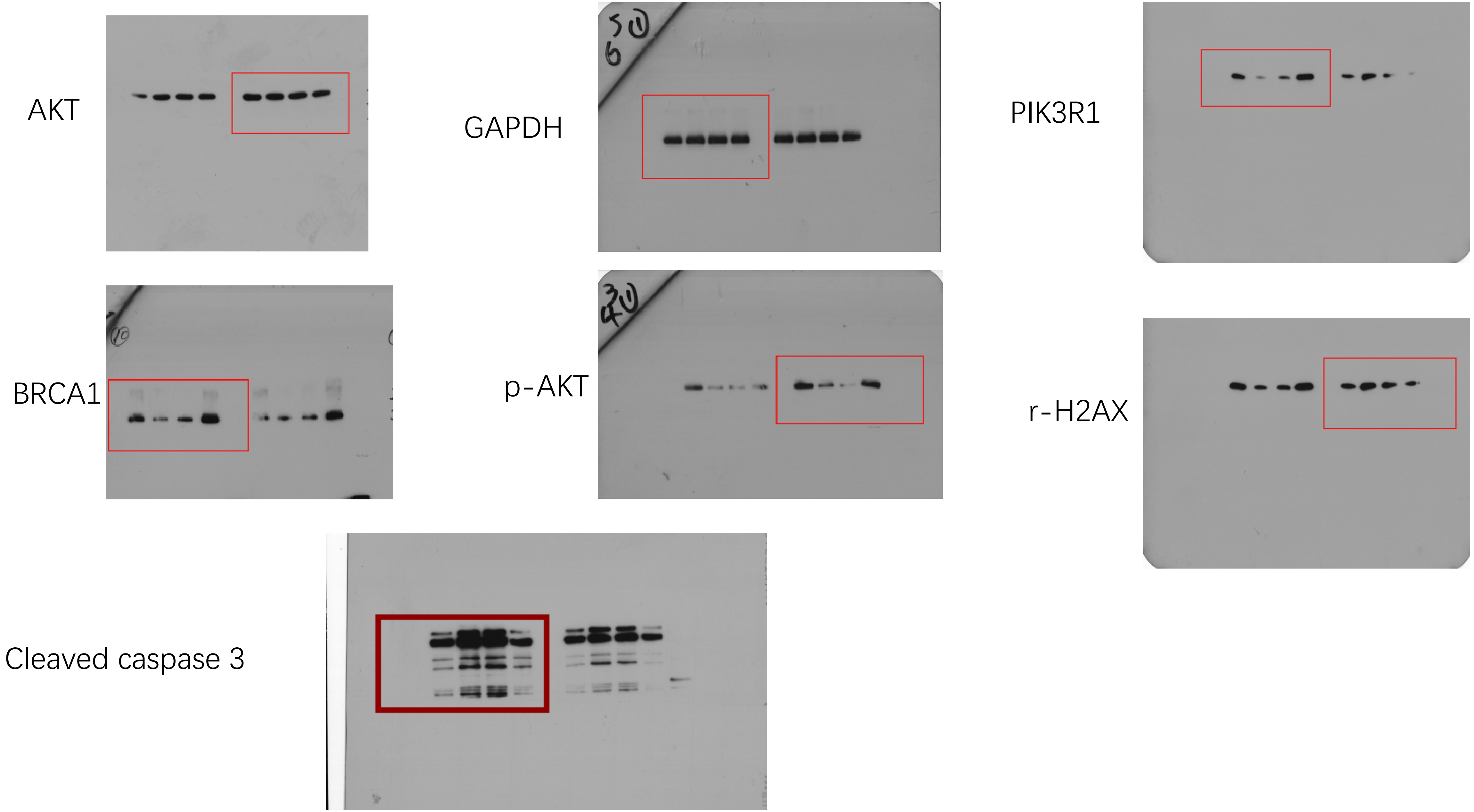

**Figure 4M SKOV3-CDDP**

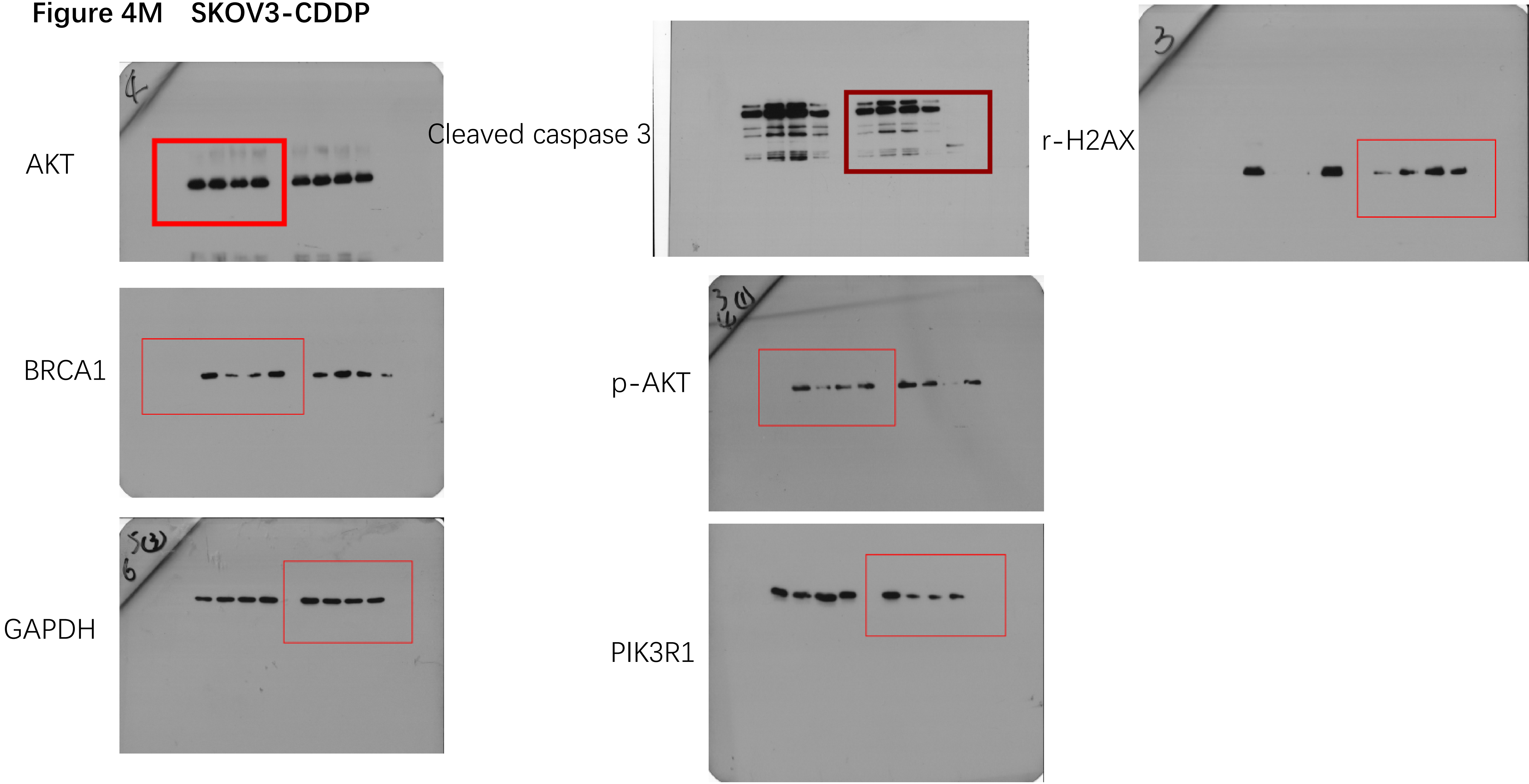

**Figure 5F**

A2780-CDDP

METTL3

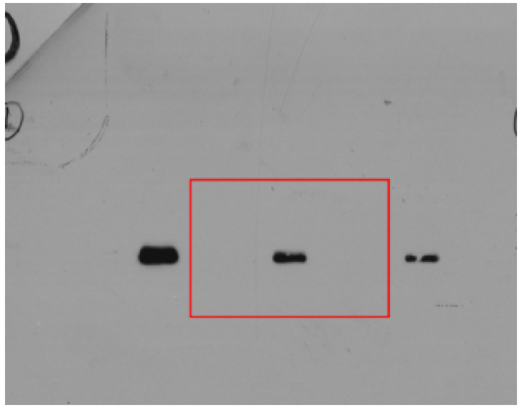

SKOV3-CDDP

METTL3

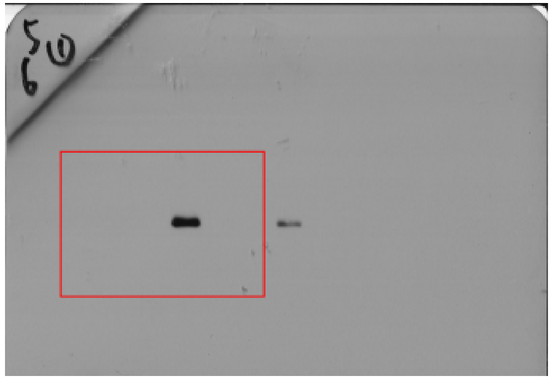

**Figure 5G**

A2780-CDDP

IGF2BP1

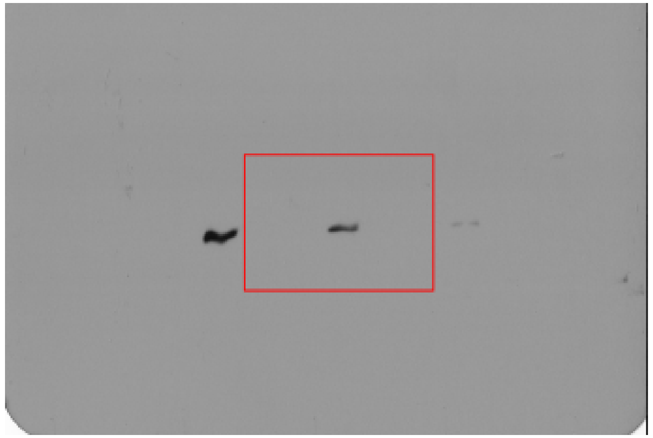

SKOV3-CDDP

IGF2BP1

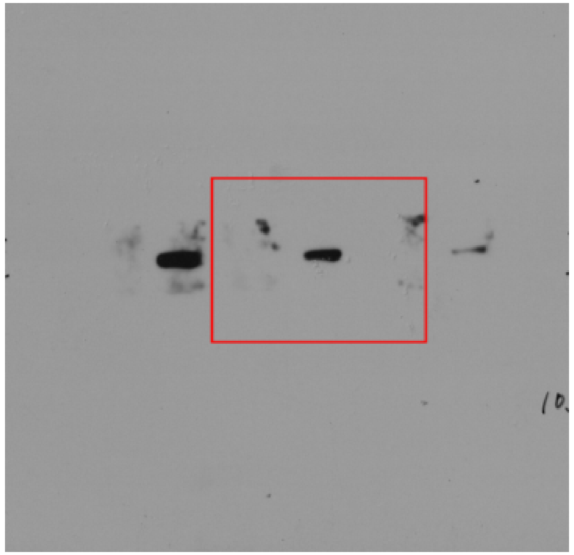

## Figure 5H

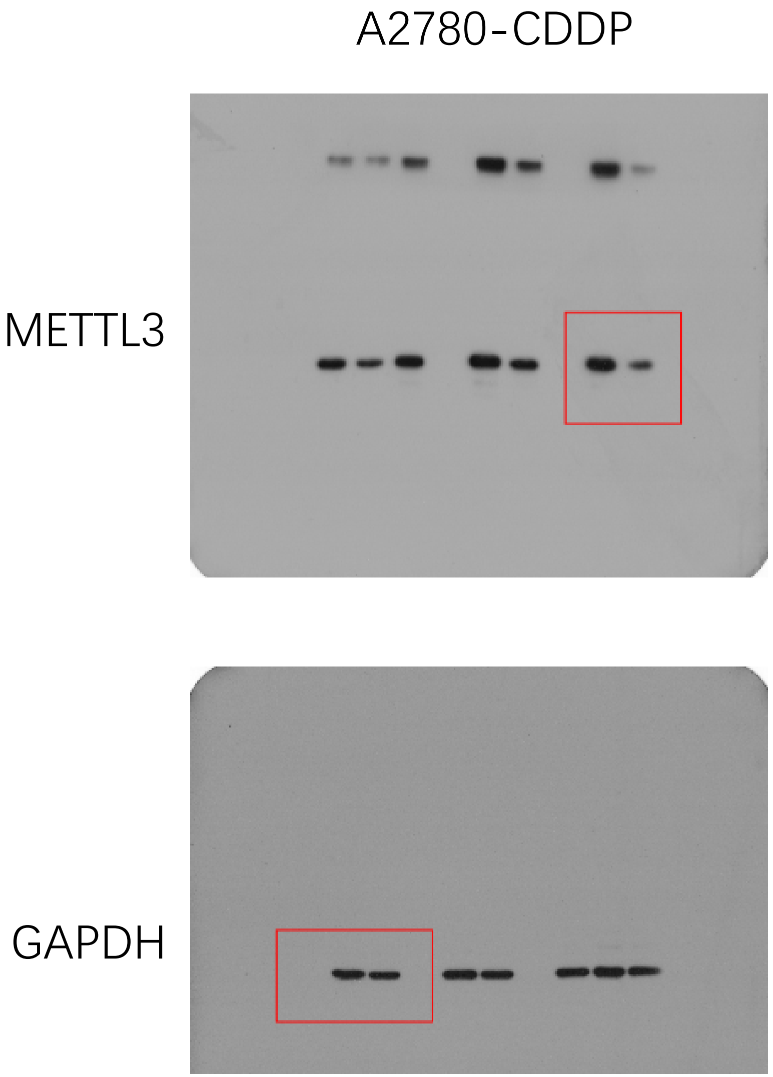

**Figure 5H**

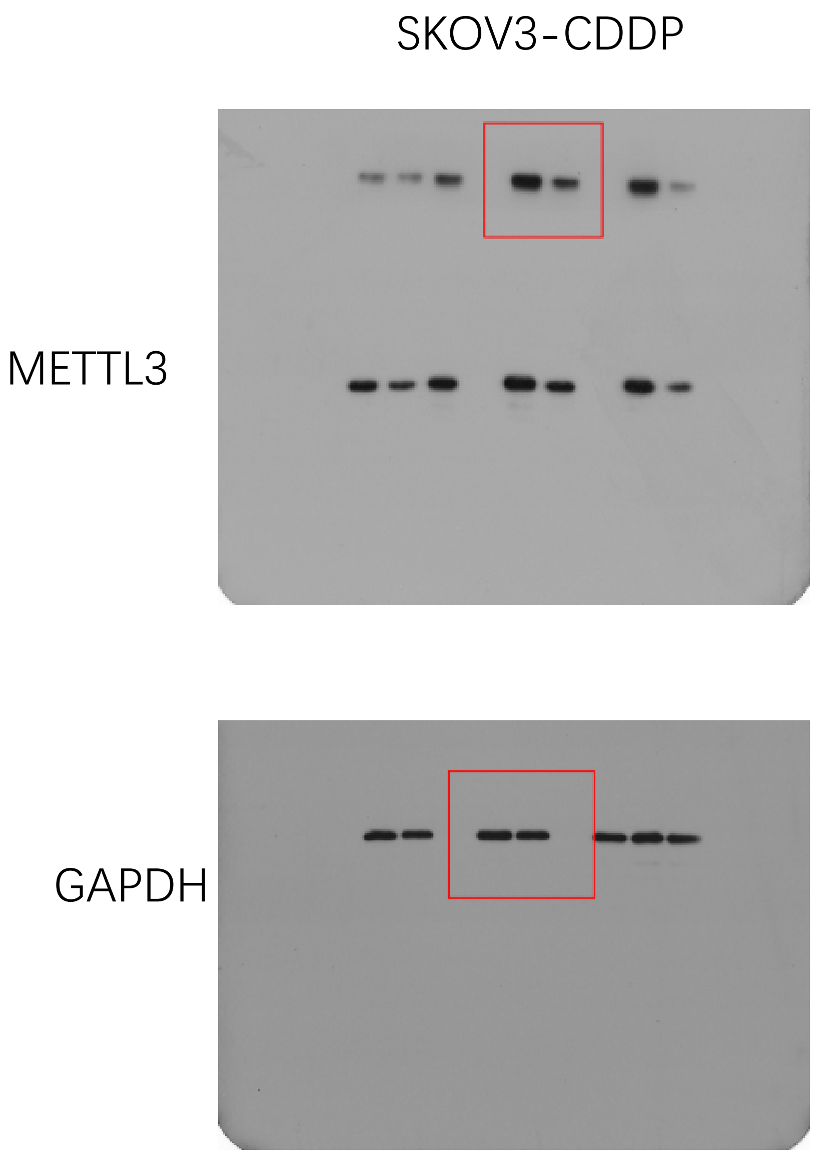

**Figure 7P**

GAPDH

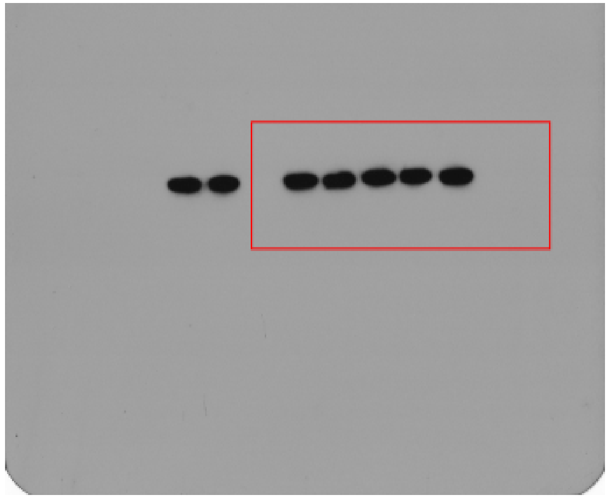

METTL3

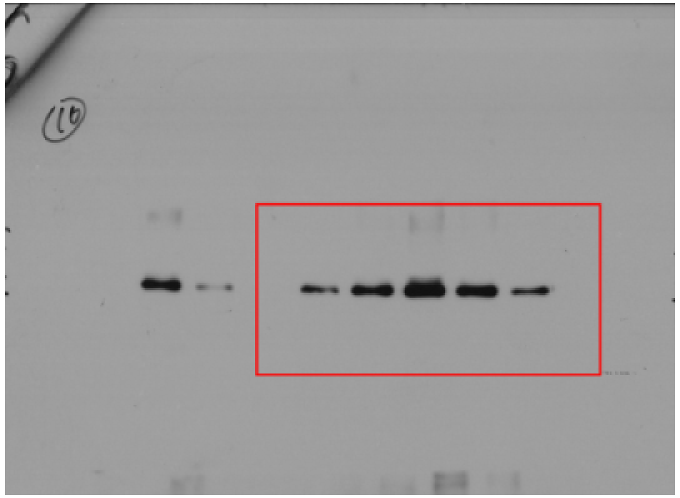

IGF2BP1

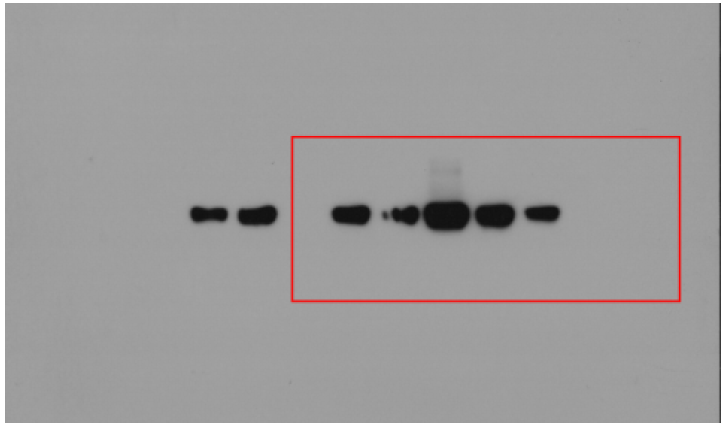

PIK3R1

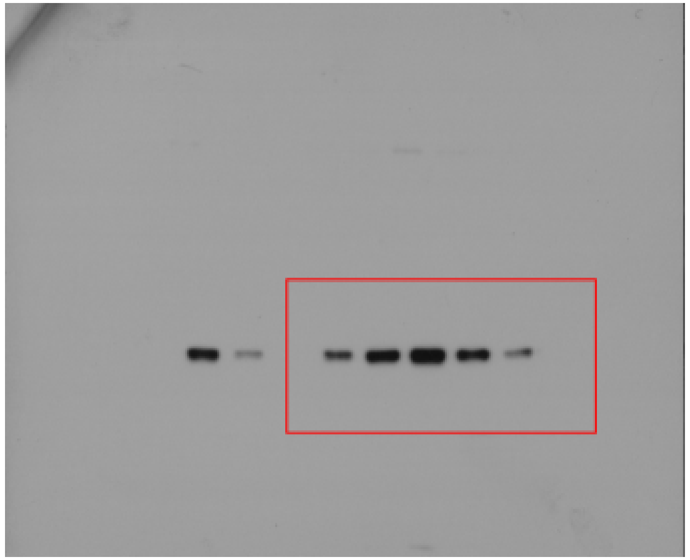

## Supplemental Figure 3 F

Cleaved caspase 3

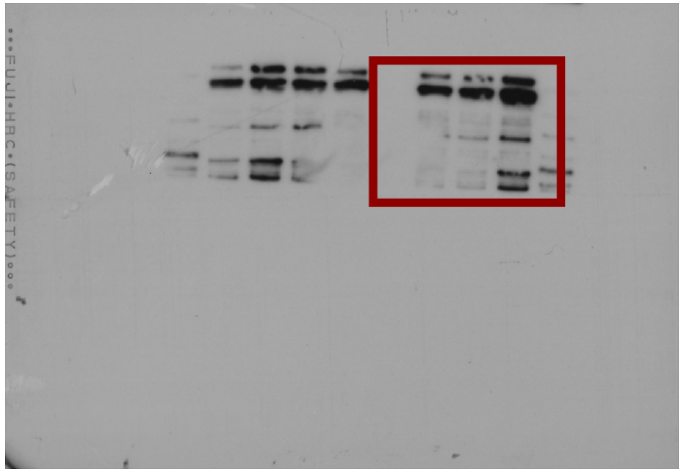

GAPDH

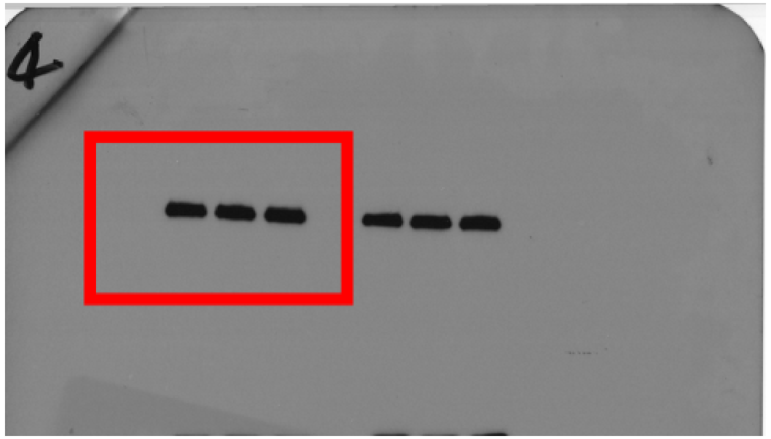

Cleaved PARP

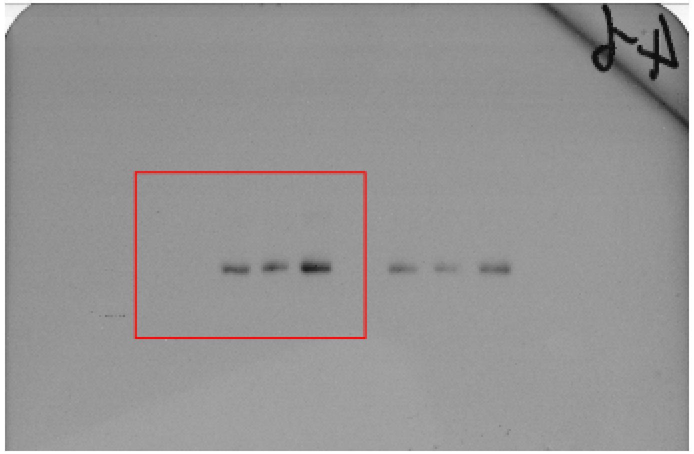

PARP

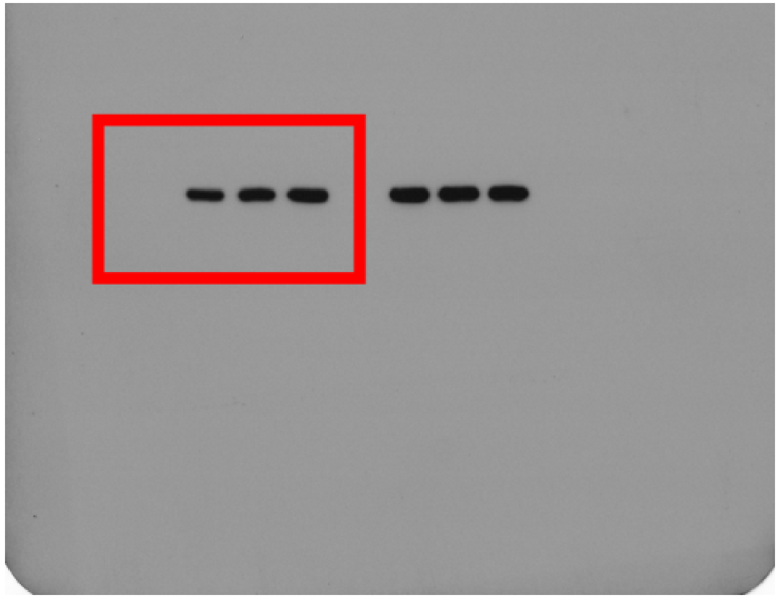

**Supplemental Figure 3 G**

BRCA1

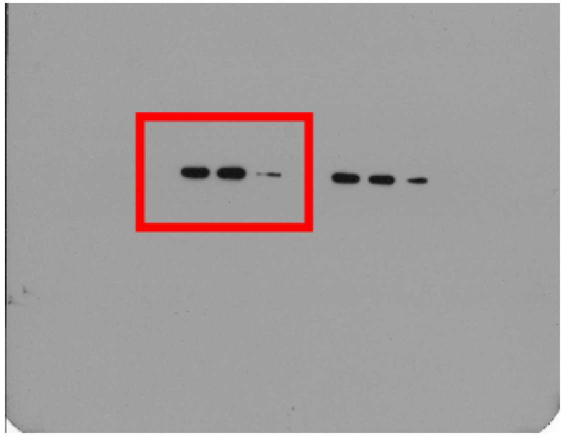

r-H2AX

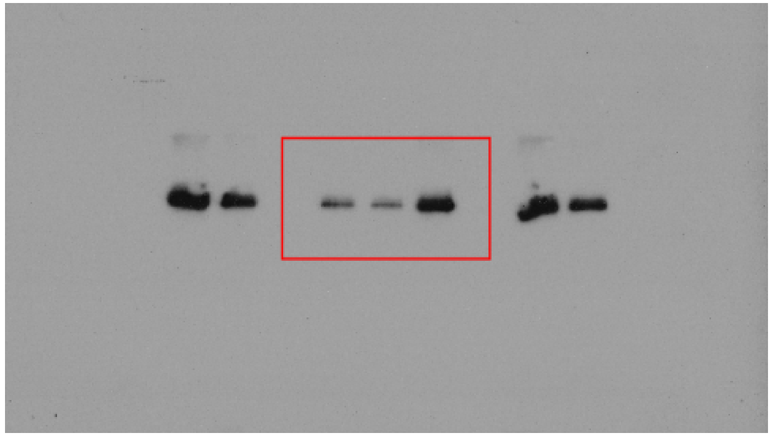

GAPDH

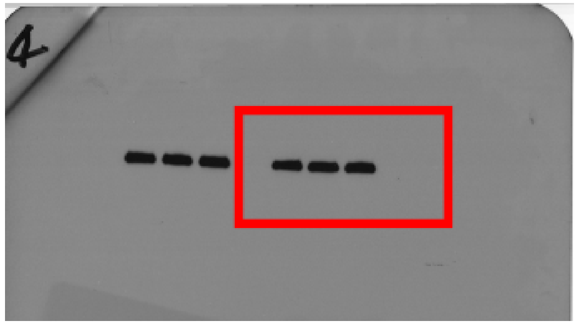

## Supplemental Figure 4 F

Cleaved caspase 3

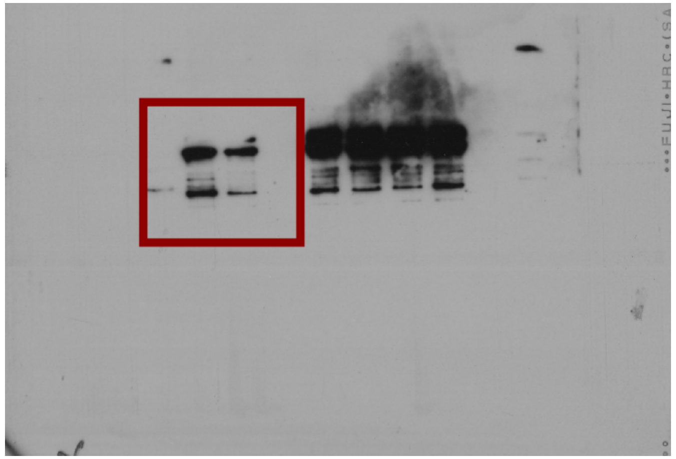

GAPDH

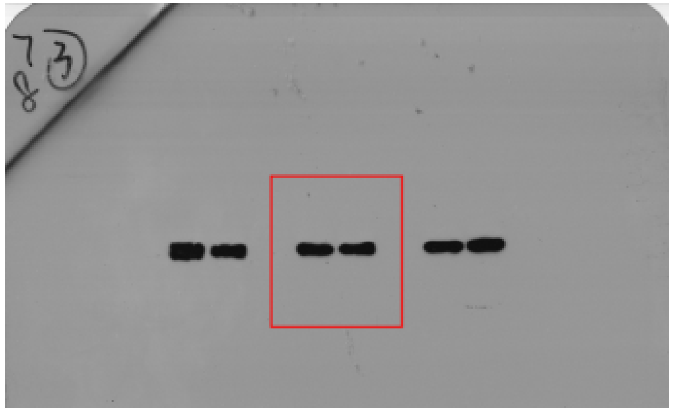

Cleaved-PARP

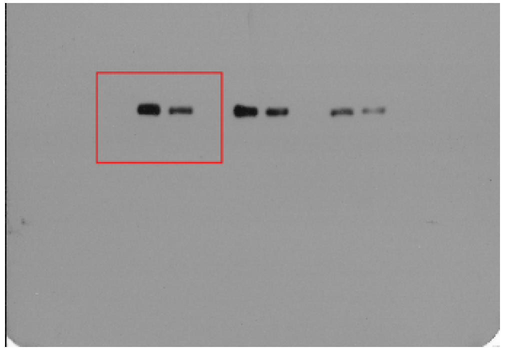

PARP

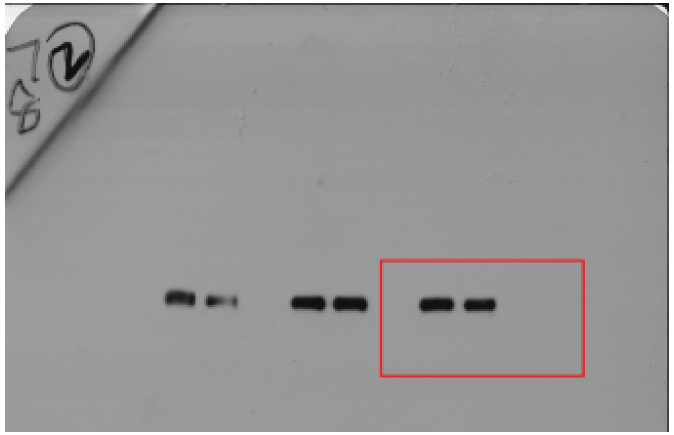

# Supplemental Figure 4 G

BRCA1

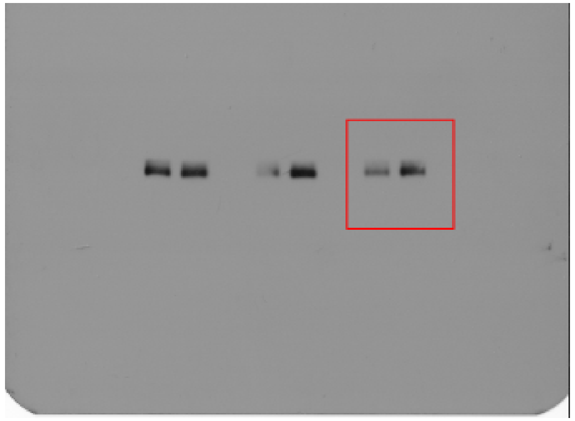

r-H2AX

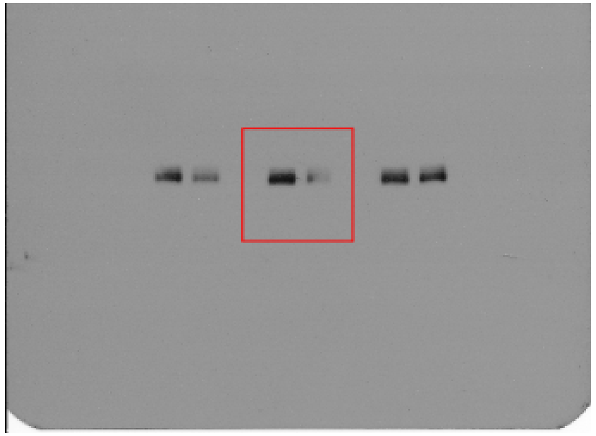

GAPDH

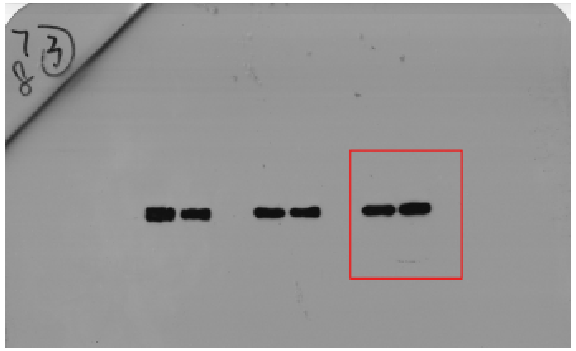

**Supplemental Figure 5D**

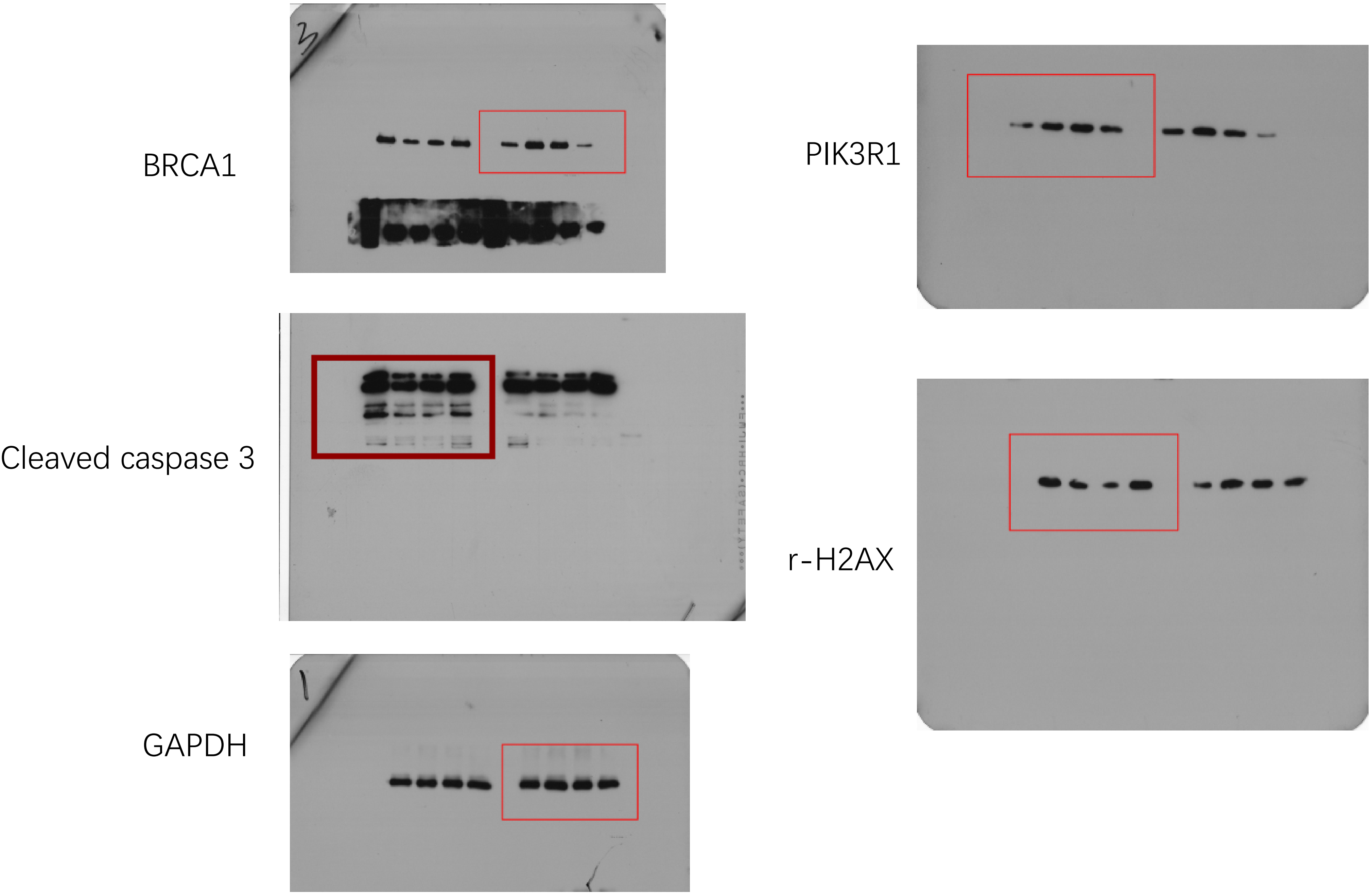

## Supplemental Figure 5E

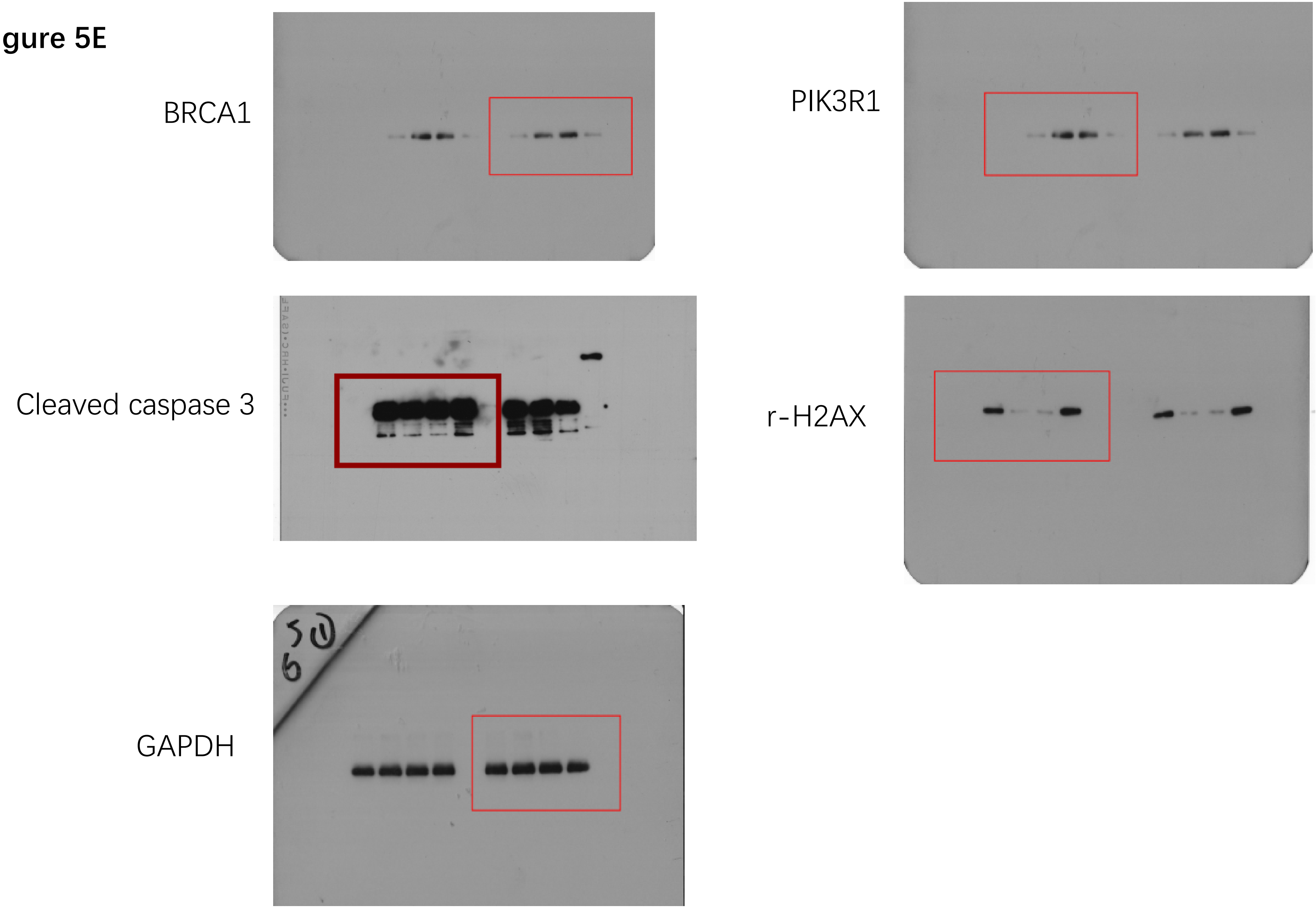

## Supplemental Figure 7 D

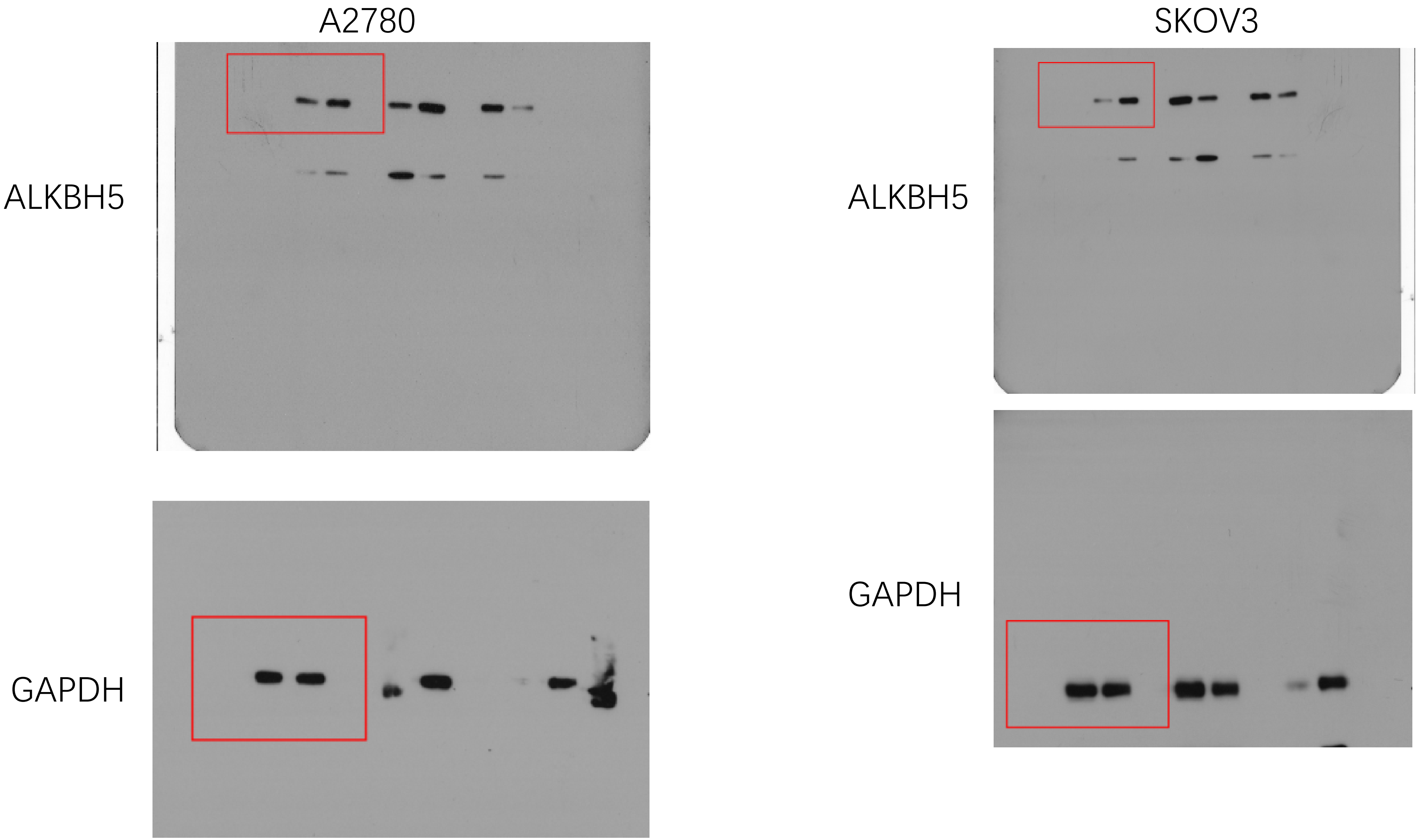

# Supplemental Figure 8A

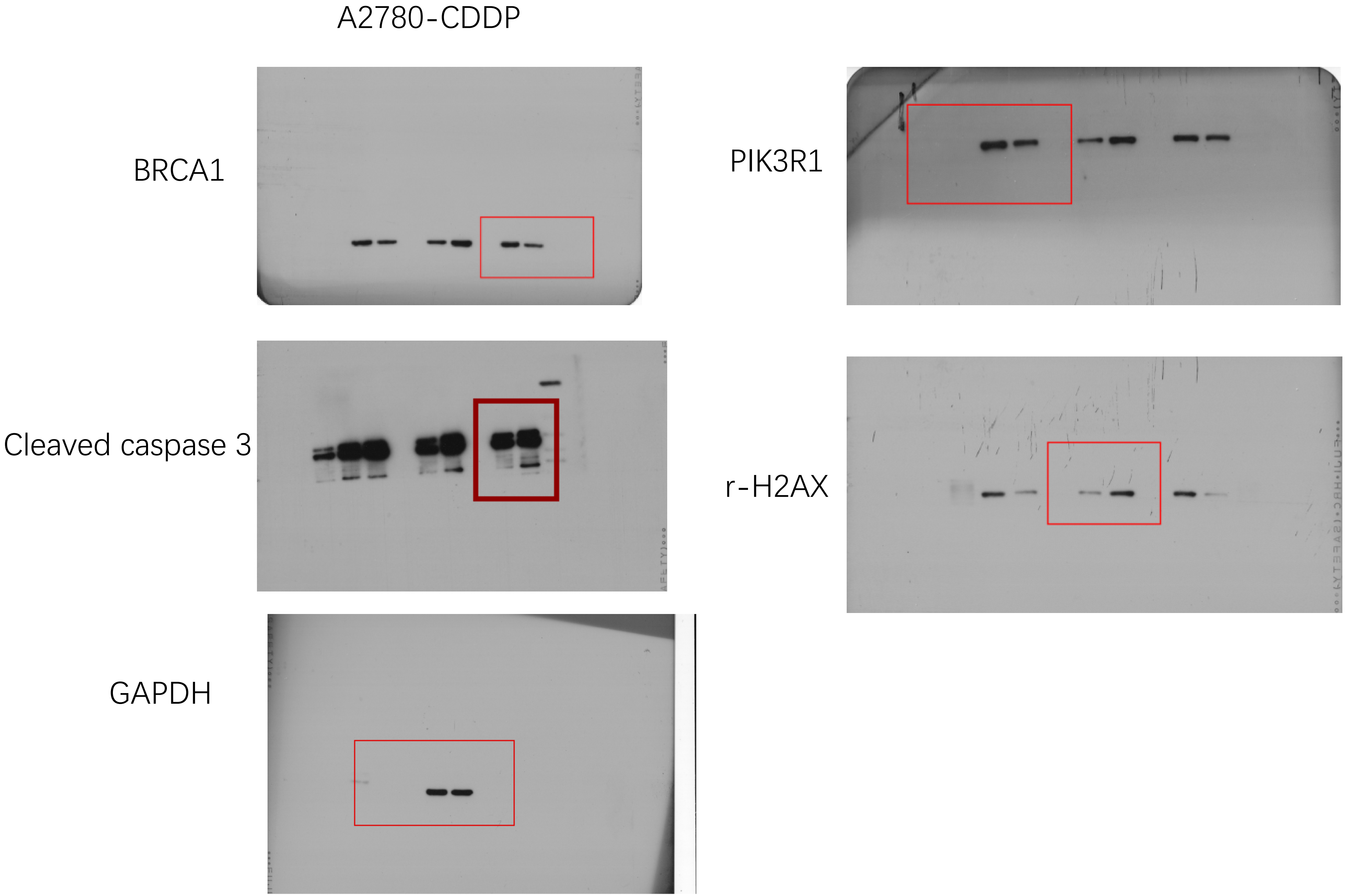

Supplement: Supplementary file 9 — Additional file 9: Supplemental Figure 4. circPLPP4 confers CDDP resisitance of CDDP in OC cells. (A) qRT-PCR analysis of circPLPP4 and PLPP4 expression in the indicated OC cells. (B, C) MTT cell viability assay of CDDP in the indicated cells. (D, E) Quantification of colony number of the indicated cells. (F) Western blotting analysis of level of caspased 3, cleaved-caspased 3, PARP and cleaved-PARP in the indicated cells. (G) Western blotting analysis of level of γ-H2AX and BRCA1 in the indicated cells. (H, I) FACS analysis of Annexin V/PI staining of indicated cells with vehicle or CDDP (5μM) treatment after 24 hours. (J, K) Quantification of γ-H2AX in the indicated OC cells with CDDP (5μM) treatment after 24 hours. * P < 0.05, ** P < 0.01, *** P < 0.001, **** P < 0.0001, ns indicates no significance. Each error bar represents the mean ± SD of three independent experiments. [file Only for reviewer 3-WB raw data.pdf › Only for reviewer 3-WB raw data.pdf]
